# Current Progress on the Influence Human Genetics Has on the Efficacy of Tyrosine Kinase Inhibitors Used to Treat Chronic Myeloid Leukemia

**DOI:** 10.7759/cureus.56545

**Published:** 2024-03-20

**Authors:** Tara C Prakash, Steven Enkemann

**Affiliations:** 1 Department of Cell Biology and Physiology, Edward Via College of Osteopathic Medicine, Spartanburg, USA

**Keywords:** dasatinib, bosutinib, nilotinib, imatinib therapy, pharmacogenetics, tyrosine kinase inhibitors, chronic myeloid leukemia (cml)

## Abstract

The use of tyrosine kinase inhibitors (TKIs) has become the mainstay of treatment in patients suffering from chronic myeloid leukemia (CML), an adult leukemia caused by a reciprocal translocation between chromosomes 9 and 22, which creates an oncogene resulting in a myeloproliferative neoplasm. These drugs function by inhibiting the ATP-binding site on the fusion oncoprotein and subsequently halting proliferative activity. The goal of this work is to investigate the current state of research into genetic factors that influence the efficacy of four FDA-approved TKIs used to treat CML. This overview attempts to identify genetic criteria that could be considered when choosing one drug over the others and to identify where more research is needed. Our results suggest that the usual liver enzymes impacting patient response may not be a major factor affecting the efficacy of imatinib, nilotinib, and bosutinib, and yet, that is where most of the past research has focused. More research is warranted on the impact that human polymorphisms of the CYP enzymes have on dasatinib. The impact of polymorphisms in UGT1A1 should be investigated thoroughly in other TKIs, not only nilotinib. The role of influx and efflux transporters has been inconsistent thus far, possibly due to failures to account for the multiple proteins that can transport TKIs and the impact that tumors have on transporter expression. Because physicians cannot currently use a patient’s genetic profile to better target their treatment with TKIs, it is critical that more research be conducted on auxiliary pathways or off-target binding effects to generate new leads for further study. Hopefully, new avenues of research will help explain treatment failures and improve patient outcomes.

## Introduction and background

Chronic myeloid leukemia (CML) accounts for approximately 15% of all leukemias in adults [1]. CML develops when hematopoietic stem cells acquire a reciprocal translocation between chromosomes 9 and 22 that creates a fusion between the breakpoint cluster region (BCR) on chromosome 22 and the Abelson (ABL) tyrosine kinase on chromosome 9. This fusion creates an oncogene that constitutively phosphorylates multiple targets of the ABL-tyrosine kinase, ultimately resulting in a myeloproliferative neoplasm [2].

Prior to the 21st century, CML was managed through radiation, stem cell transplantation, and interferon-based therapy. Despite mild upward trends in survival rates with the use of interferon therapy or bone marrow transplants, CML was still deemed fatal with a five-year survival rate of 31% in the 1990s [3,4]. It was in 2001, when the first generation of tyrosine-kinase inhibitors (TKIs) was introduced, that scientists truly saw breakthrough improvements in patient outcomes and survival. With TKIs as first-line treatment, five-year survival rates for patients with CML went from 31% in the 1990s to 70.6% in patients diagnosed from 2013 to 2019 [5].

This drastic improvement in patient outcomes is due to the highly effective mechanism of action of the TKIs. Protein tyrosine kinases (PTK) mediate many signaling pathways including those that influence cell growth, proliferation, and differentiation. Normally, these proteins are tightly regulated. However, in cancers such as CML, unregulated PTK activity leads to malignant transformation of the cell [6]. The BCR/ABL fusion proteins in CML are unregulated kinases that phosphorylate Gab2, Myc, CrkL, and STAT5 along with many other proteins within the cell [7]. These specific substrates lie on well-documented pathways toward growth, replication, and transformation. TKIs, as homologs of adenosine triphosphate (ATP), function as specific competitive inhibitors of the ATP-binding sites on protein tyrosine kinases. By inhibiting ATP-binding and thus, phosphorylation of targets, TKIs prevent any subsequent downstream activities such as cell growth and proliferation. The TKIs designed to compete for the ATP binding site of the BCR-ABL fusion protein have been shown to be useful in not only CML but also a variety of other cancers including some non-small-cell lung carcinomas (NSCLC), renal cell carcinomas (RCC), and gastrointestinal stromal tumors (GIST) [6].

The way in which the TKIs bind to the ABL kinase domain can impact the activity and the opportunity for secondary effects. Two of the TKIs discussed, imatinib and nilotinib, are Type II kinase inhibitors, meaning they bind to the inactive conformation of protein kinases. Dasatinib, a second-generation TKI, is both a Type I and a Type II kinase inhibitor, meaning it binds to both the active and inactive conformation of the ABL1 kinase [6]. Bosutinib, a fourth drug designed against the BCR-ABL fusion protein is neither a Type I nor Type II inhibitor as it binds to the protein while it is somewhere between an active and inactive transitional conformation [7]. In general, it has been assumed that Type 1 inhibitors are less specific than Type 2 inhibitors because the active conformation is very similar in most kinases [8]. Yet surprisingly, these ATP mimics are relatively precise in their binding specificity, exhibiting few off-target binding sites [9]. Each off-target enzyme provides an opportunity for side effects and variation in how individuals respond to treatment.

As cancer treatment moves toward personalized therapy, these TKIs used in CML serve as distinguished examples of targeted therapy due to their high efficacy along with low side effects. However, imatinib, the first TKI to achieve FDA approval, has not been the panacea initially expected for CML. Use of imatinib as first-line therapy does not result in complete cytogenetic responses in up to 35% of patients with CML [10, 11]. Due to resistance mechanisms and other tumor characteristics, second-generation TKIs have been developed to try to close this gap. However, tumors persist when second-generation TKIs are used and therefore third-generation TKIs exist. These may not yet be realistic options due to severe side effects and affordability [12]. The question remains as to what can be done to improve patient results with the first- and second-generation TKIs.

This review aims to discuss genetic factors that may influence the efficacy of four FDA-approved TKIs used in the treatment of CML, imatinib, nilotinib, dasatinib, and bosutinib, with the goal of establishing criteria about patients that might improve efficacy and minimize adverse reactions during treatment. The most common mechanism of CML resistance occurs because of mutations to the ATP binding site of the BCR-ABL fusion protein within the tumor. This subject is left to others as many overviews are available, focusing on the mutations themselves or the drugs that can overcome certain resistance mutations [13-17]. Research has now progressed from discovering mutations to using that knowledge to improve the use of the first-, second-, and now third-generation TKIs. There are also numerous reviews about the metabolism of BCR-ABL TKIs [2,18-20]. While these reviews summarize current knowledge of the usual liver enzymes involved in pharmacogenetics, they do little to push research closer to clinical solutions. The current review attempts to highlight gaps in our understanding of why certain patients do not respond to treatment by illustrating where more research is needed and describing novel research that is emerging.

## Review

Imatinib

The first TKI developed for the inhibition of the BCR-ABL gene was imatinib. Imatinib mesylate (trade names Glivec, Gleevec) was approved by the FDA in 2001 and was the drug of choice for patients with CML for decades. Imatinib functions as an ATP-analog that targets the phosphate-binding domain of BCR-ABL [2] and also has activity against the c-KIT and PDGFR tyrosine kinases [21]. Importantly, imatinib has a relatively low toxicity profile when compared to alternative TKIs. A systematic review by Vener et al. found that imatinib is often preferred in patients with comorbidities since most of its adverse effects, such as weight gain from fluid retention, muscle pain, and rashes, are, in most instances, easily manageable and temporary [22]. However, as many as 35% of CML patients will discontinue imatinib due to lack of efficacy, intolerance, or developed resistance by the tumor, usually by mutations occurring in the BCR-ABL kinase domain [10]. 

CYP Enzymes

Imatinib is cleared from the blood by the liver more so than by renal excretion. Liver enzymes N-demethylate the compound to N-desmethyl imatinib (NDIM), which is 3-10-fold less potent than imatinib, but only about 20% of the administered dose is so modified at any time [23]. Liver metabolism was originally thought to occur mainly through the cytochrome P450 enzyme CYP3A4; however, current thinking is that CYP2C8 and possibly CYP3A5 carry out most of the metabolism while CYP3A4 and possibly CYP2C9, CYP2C19, and CYP2D6 playing minor roles [24]. Furthermore, imatinib has also been shown to have inhibitory activity against CYP3A4, CYP3A5, and UGT1A1 [25,26]. The interactions between imatinib and respective liver enzymes are summarized in Table 1.

**Table 1:** Interactions between BCR-ABL tyrosine kinase inhibitors and metabolizing liver enzymes Blank spaces in the table indicate that the interaction was either not tested or not reported. * indicates the primary interaction. CYP: cytochrome P-450; UGT: uridine diphosphate-glucuronyltransferase; BCR: breakpoint cluster region; ABL: Abelson

Liver Enzymes	Imatinib	Nilotinib	Dasatinib	Bosutinib
CYP 3A4	Substrate*, Inhibitor	Substrate*	Substrate*	Substrate*
CYP 3A5	Substrate*, Inhibitor	Inhibitor		
CYP2B6		Inducer		
CYP 2C8	Substrate*	Inhibitor, Inducer		
CYP 2C9	Substrate	Inhibitor, Inducer		
CYP 2C19	Substrate			
CYP 2D6	Substrate	Inhibitor		
CYP 2D8		Inhibitor		
UGT1A1	Inhibitor	Inhibitor		
Drug Half-life	18-40 hrs	16 hrs	3-5 hrs	18-40 hrs

The low amount of metabolism, the small sample sizes in the studies, and the fact that perhaps the wrong enzyme(s) were evaluated may have all contributed to the current belief that pharmacogenetics may not influence imatinib efficacy. This should be more thoroughly investigated. A minor metabolizer, by itself, may have little effect on the net concentration of active compounds within the tumor. This can make it difficult to see a difference in individuals with a defective enzyme. But, when combined with a second minor contributing enzyme, a measurable difference may emerge. The identification of such a situation only happens if all the contributors are identified, are investigated together, and can be combined to ask if two or more defective alleles have more impact than just one. This also includes the enzymes and transporters described below.

Transporters

Imatinib is the only TKI discussed in which both influx (SLC22A1 (OCT1) and OATP1A2) and efflux (ABCB1, ABCC4, and ABCG2) transporters have been shown to interact with the drug [2]. Though influx transporters are consistently cited as having some role in imatinib activity, its exact role has yet to be agreed upon. Some studies state that imatinib has a purely inhibitory role on the OCT-1 transporter [27] while others state it has no role [28]. Similarly, some studies claim that no uptake transporters investigated to date (SLCO1A2, SLCO1B1, SLCO1B3, SLC22A2-8, SLC47A1) have been shown to significantly influence imatinib intracellular accumulation [23] while review articles imply that some variants of these transporters may influence activity [18]. The lack of agreement here suggests that further work is required.

Tumor resistance is the leading cause of treatment failure, most commonly through point mutations in the BCR-ABL kinase domain, with T315I being the most well-studied and frequent mutation. However, overexpression of ABCB1 (also known as P-glycoprotein or multi-drug resistance protein 1) is another well-established mechanism of tumor resistance against TKIs. ABCB1 is highly polymorphic in the human population, with over 50 existing single nucleotide polymorphisms (SNPs) recognized, with more yet to be identified [29]. Although this seems like a promising avenue for research, results from studies examining variants of the ABCB1 transporter have been unclear thus far; the three most common SNPs in the ABCB1 gene (1236C>T, rs1128503; 2677G>T, rs2032582; and 3435C>T, rs1045642) have been repeatedly investigated and conclusions have been contradictory [29,30]. A review by Barratt et al. discussed the role of genetic variability in transporters, suggesting that they have no reproducible effect on plasma imatinib concentrations or clearance [23]. Plasma concentrations may reflect the role of transporters in clearing the drug from the bloodstream, but this is only part of the story about antitumor efficacy. Transporters play a role in the concentration of the drug inside tumor cells as well. It is well known that tumors often overexpress transporters as a mechanism of resistance and overexpression can offset low activity. Therefore, tumor expression should be assessed along with allele functionality. This is true for imatinib and for many other drugs investigated for the role polymorphisms in the ABC transporters play in treatment failures [31].

Compared to the ABCB1 transporter, ABCG2 and SLC22A1 transporters have been less investigated. In their meta-analysis, Barratt et al. found that while the ABCG2 421C>A (Q141K, rs2231142) variant appears to be associated with improved treatment outcomes in Asian CML patients, the mechanism of this association is unknown. Study limitations make it difficult to know if the genetic variability could be related to the imatinib distribution within the liver, within the bloodstream, or within the cytosol of CML cells in vivo [23]. It was shown that homozygotes for the major allele (G/G in ABCG2, rs2231137) were repeatedly associated with poor response to imatinib in individuals with advanced-phase CML [32]. Similarly, homozygosity for the major allele (C/C in ABCG2, rs2231142) was also found to be an independent predictor of a more frequent need for imatinib dose escalation [32]. These findings suggest that lower-activity transporters may have a higher antitumor efficacy. However, future studies should include more transporters to determine if all reduced function alleles produce the same trends. Kim et al. found that the minor allele at SLC22A1(rs683369) along with an advanced tumor phase correlated with a high rate of loss of response or treatment failure. They also found that homozygotes for the minor allele (G/G) and advanced stage, correlated with a high rate of loss of response or treatment failure to IM therapy [32]. This finding was supported in a meta-analysis done by Cargnin et al., although this study pointed out the need for further corroboration [19]. These relatively consistent findings in the lesser-studied enzyme (SLC22A1) suggest that most studies may be looking at the wrong transporters. However, there may be other reasons for marginal, inconsistent, or non-significant outcomes. The inconsistent results for the role of genetic differences in transporters could be due to the ubiquity of their expression in multiple tissues, the ability of many tumors to overexpress these proteins, or the fact that multiple transporters could be acting in concert so that no single variant strongly impacts the overall transport capacity for a cell. There is enough evidence to warrant further research into how each TKI is transported into and out of cells so that the right combination of transporters, and their variants, are considered in future studies assessing antitumor efficacy.

Additional Considerations Impacting Patient Response

Once the drug has been administered, it becomes available in plasma, where it is bound primarily to albumin and alpha1-acid glycoproteins (AAG) [2]. Plasma AAG levels vary substantially between individuals which could affect imatinib clearance rates and availability to tumor cells. There are also three main variants distributed in the human population which could influence how much drug is bound to protein or free to enter tumors. Studies to date have not investigated how genetics impacts this or influences further drug metabolism [33,34]. AAG also binds to many different xenobiotic compounds and thus polypharmacy becomes an issue. It is known that certain drugs can displace others on AAG, but nothing is known about how other drugs may impact the carrying capacity for TKIs [35].

In 2021, Cereja et al. investigated 32 polymorphisms in 24 genes and found that individuals homozygous for the minor allele at rs2372536 (a missense variant in the ATIC gene) were approximately three times more likely to experience treatment failure with imatinib and that individuals who were homozygous for the major TT allele at rs10821936 (an intronic variant in the ARID5B gene) were also at a higher risk for treatment failure with imatinib [36]. It is not clear what role these genes play in imatinib metabolism, tumor cell growth, or some other aspect of treatment. However, the study illustrates the fact that the lack of effectiveness for imatinib in some patients could be explained by aspects of drug metabolism that have not yet been considered [36]. ATIC catalyzes the last two steps of the de novo purine biosynthesis and may influence how much ATP is available to compete with TKIs in tumors while ARID5B is a DNA-binding protein that participates in the epigenetic regulation of the genome. ARID5B variants may play a role in the development of acute lymphoblastic leukemia (ALL) [37]. Studies in ALL reveal a number of human variants that should be tested in CMLs treated with TKIs.

The platelet-derived growth factor receptors (PDGFR) are tyrosine kinases that are secondary targets of imatinib and nilotinib [9]. PDGFRs are cell surface receptors that when stimulated induce cellular growth associated with wound healing, angiogenesis, and the development of certain structures within the body. Variants in either the alpha or beta PDGFR might be an important area to investigate relative to electrolyte imbalances that occur in some patients, as a review by Prenggono et al. concluded [38]. They observed that blood calcium and potassium levels tend to decrease in some patients treated with imatinib and postulated that, for potassium, this could be due to the role played by the PDGFR in kidney physiology, where it is needed for renal tubular regeneration. When imatinib inhibits the PDGFR, it inhibits the tubulogenic process reducing the number of tubular cells. With fewer tubular cells, potassium can no longer be reabsorbed properly. The story of hypocalcemia is less clear. Calcium levels in patients taking imatinib range from the lower normal values to hypocalcemia but no clear explanation for this has been established [38]. These trends may be of particular importance in patients who already suffer from renal and/or cardiac issues. But still undefined is the role genetics may play in these side effects.

Imatinib treatment is now being considered for other disorders that result from activating variants in the beta PDGFR [39]. This is further evidence that while treating tumors, imatinib also interacts with the PDGFR and that people sometimes possess variants of that receptor. By itself, this should be evidence that genetic variation in the PDGFR should be considered when treating with imatinib. However, there is additional evidence, in mice, that the inhibitory effect of imatinib on tumor cells correlates better with its activity against the PDGFR than its activity against KIT or Abl [40]. The results from this study suggest that the anti-tumor efficacy of imatinib may be partially dependent on the PDGFR. 

Another important area of future genetic research comes from work with leukemic stem cells (LSCs). TKIs often fail to eliminate LSCs, allowing persistence and further development of resistant disease [41,42]. A review by Arrigoni et al. suggested that LSCs may activate the efflux pumps (i.e., ABC transporters), which might explain their resistance to TKIs, and that low-activity uptake transporters (i.e., hOCT1) may be associated with a poor response to therapy [43]. This further underscores the idea that genetic variants in transporters may play a role in the overall efficacy of TKIs, but researchers have to better understand which transporters are responsible for this effect and where they are when they influence activity.

Nilotinib

Nilotinib is a second-generation TKI that is most often the first alternative choice to imatinib. Nilotinib is also an ATP analog but with the methylpiperazine group found on imatinib substituted with a trifluoromethyl and a methylimidazole group. By altering the structure of imatinib, nilotinib becomes 30-fold more selective for the binding site on the BCR-ABL target [2]. While this increased potency allows for efficacy against some imatinib-resistant point mutations, its identical mechanism of action allows for some common resistance liabilities. These include certain mutations of the fusion protein (Y253, E255, T315, and F359) that provide resistance to both imatinib and nilotinib [44]. Moreover, nilotinib also has activity against the PDGFR, c-KIT, and the discoidin domain receptors -1 and -2 (DDR1 and DDR2) [45].

Importantly, nilotinib reduces the expression of the alpha subunit of the potassium ion channel (KCNH2) which is critical for regulating the human ventricle and effectively controlling the QT interval. This ultimately leads to a proarrhythmic effect of nilotinib [46]. Carracedo et al. showed that nilotinib increased murine aortic valve thickness, promoted calcification and osteogenic activation, and decreased autophagy in human valvular interstitial cells suggesting that nilotinib may have additional cardiotoxicity in some patients [47]. A meta-analysis from 2022 concluded that nilotinib should not be recommended to patients with advanced age, previous cardiovascular disease, high-risk factors, or a previous cardiovascular adverse event, and that nilotinib should be completely avoided as a first-line drug because of its cardiotoxicity [48]. As this does not affect all patients, this would be a good place to understand the role of genetics in this process, and possibly return nilotinib to frontline use in some patients.

Liver Enzymes

Nilotinib is primarily metabolized by CYP3A4. The drug also competitively inhibits the hepatic enzymes CYP3A5, CYP2C8, CYP2C9, CYP2D6, and UGT1A1 [2] and is an inducer of CYP2C8, CYP2C9, and CYP2B6 [1]. This suggests that nilotinib could alter the metabolism of other drugs taken at the same time. The drug itself has a half-life of 16 hours and is excreted mainly through feces with 69% of the drug excreted unchanged, indicating that the liver plays a major role in excretion but a minor role in its metabolism [1].

It has been well established that variations in the UGT1A1 gene have major implications on the levels of toxicity of nilotinib. Abumiya et al. found that the patients who developed hyperbilirubinemia were more often those who possessed one of the two main deficient alleles of UGT1A1 [49]. The *6 variant is a glycine-71 substitution with arginine and the *28 variant is the presence of an additional TA repeat in the TATA sequence of the UGT1A1 promoter. Both variants are associated with a poor ability to metabolize many drugs [49]. The *6 variation is common in Japanese patients and the correlation between Japanese patients and nilotinib-induced hyperbilirubinemia has been well-established [49]. A study by Shibata et al. provided further evidence for this phenomenon, showing that patients with two defective alleles had even more severe side effects [50]. Because the allele frequency of *6 is 15.7% in the Japanese population, as compared with only 0.7% in the Caucasian population, they recommend that genetic screening should be done in all Japanese patients for both UGT1A1*28 and UGT1A1*6. In addition to Japanese patients, patients who have been diagnosed with Gilbert’s syndrome should avoid nilotinib [51]. Bykova et al. found that patients who had the homozygous genotype of the (TA)7/(TA)7 (*28/*28) for UGT1A1 were more likely to have grade 3 hyperbilirubinemia (mostly of the indirect bilirubin fraction) when treated with nilotinib [51]. Pharmacogenetic analysis of patients who will be taking nilotinib should begin with these variants of UGT1A1.

Transporters

Unlike imatinib, the uptake of nilotinib is primarily passive as influx transporters have not demonstrated any influence in efficacy or metabolism. Instead, nilotinib’s high hydrophobic nature plays a role. Taking the drug with a meal containing fat increases its bioavailability from 30% to 50%. Like imatinib, nilotinib interacts with both ABCB1 and ABCG2 efflux transporters, though it is thought to have a higher affinity for ABCB1 [2]. Eadie et al. found that nilotinib functions as a substrate of ABCB1 at lower nanomolar concentrations and an inhibitor of this transporter at high micromolar concentrations (>500 nmol/l), both of which are within the therapeutic range [52]. The apparent concentration dependence of the nilotinib-ABCB1 relationship was confirmed by in vitro experimentation using K562 cells transduced with ABCB1 [52]. The authors also established that some evidence exists for nilotinib acting as an ABCG2 substrate as well.

Similar to imatinib, the most common variants of ABCB1 transporters have been investigated with nilotinib, and it is important to emphasize that the results from these studies do not address the various ways that tumors may affect transporter expression. While more studies are needed, the data thus far has shown that the most common variants of the ABCB1 transporter have little impact on nilotinib blood levels or activity. Gamliberti et al. investigated the possible correlation between the three most frequent SNPs of ABCB1 (rs1128503 (c.1236C>T), rs2032582 (c.2677G>T/A), rs1045642 (c.3435C>T)) on nilotinib efficacy and toxicity [53]. They observed no difference between variant carriers and individuals with the major allele in a cohort of 78 patients [53]. This was largely corroborated by Dessilly et al., though this study was conducted in vitro [29]. Here, scientists found only minor effects of ABCB1 polymorphisms 1236T, 2677T, and 3435T when examining the impact of these polymorphisms on the anti-proliferative activity and the intracellular accumulation of TKIs in transfected HEK293 and K562 cells [29]. In a separate report by Dessilly et al., the antiproliferative effects of imatinib, nilotinib, and dasatinib were investigated in a cell line transfected with the ABCB1 1199A variant allele [54]. The study found that imatinib, nilotinib, and dasatinib are transported more efficiently by the ABCB1 variant (Asn400) protein compared with the wild-type (Ser400) protein [54]. Presumably, individuals with this variant would experience reduced efficacy of nilotinib against tumor cells.

Additional Considerations Impacting Patient Response

Nilotinib also interacts with the PDGFR, with stronger effects against PDGFR-β. This is in contrast to imatinib, which inhibits both PDGFR-α and PDGFR-β. This difference is thought to be why potassium levels tend to decrease by a lesser degree in patients taking nilotinib, than in those taking imatinib [38]. Since imatinib has been shown to have functional efficacy correlated with its activity against PDGFR-B, it is reasonable to suspect that there are similar effects with nilotinib [39]. This should be investigated. There are other off-target proteins affected by nilotinib, including the inhibition of DDR-1 and DDR-2. Nilotinib inhibition of DDR-1 may play a protective role in neurodegenerative diseases, including Alzheimer’s, Parkinson’s, and Lewy body dementia [55,56]. None of these, however, have been investigated for whether human variants affect the efficacy or tolerability of nilotinib.

Dasatinib

In 2010, another second-generation TKI named dasatinib (trade name Sprycel), was approved by the FDA for use in patients who have resistance or intolerance to imatinib and newly diagnosed CML patients who are in chronic phase [57]. The thiazole-carboxamide structure of dasatinib is unique in that it binds to both the active and inactive conformations of BCR-ABL whereas imatinib and nilotinib only bind to the inactive form of BCR-ABL [47]. Various investigations have demonstrated that this dual-binding activity makes dasatinib more efficacious in resistant and intolerant patients [57]. However, the trade-off is that dasatinib inhibits many SRC-family kinases including SRC, LCK, HCK, YES, FYN, FGR, BLK, LYN, and FRK, as well as c-KIT, PDGFR-α and -β, and ephrin receptor kinases. One or more of these additional kinases may be responsible for its increased rates of pulmonary side effects [58]. Proteomic analysis of dasatinib interactions revealed that the drug bound over 30 tyrosine and serine/threonine kinases, which is substantially more than imatinib and nilotinib [59].

CYP Enzymes

Dasatinib is also more highly metabolized by liver enzymes. Unlike the other TKIs discussed, dasatinib is usually dosed twice daily at 70 mg [60]. The half-life of dasatinib is three to five hours and only a minority of the drug (4-19%) is excreted unchanged. Most of the metabolism occurs by CYP3A4, though FMO3 and UGT enzymes play a role [2]. Five active metabolites of dasatinib have been identified: designated M4, M5, M6, M20, and M24. M4, M20, and M24 are mainly generated by CYP3A4, M5 is generated by FMO3, and M6 is generated by a cytosolic NAD-dependent oxidoreductase. M4, the primary active compound, is unlikely to play a major role in the observed pharmacology of dasatinib as the drug is well-absorbed and only a minority of it is excreted unchanged. M5 and M6 are more than 10 times less active than dasatinib and are considered minor circulating metabolites [61]. Far more research is required to establish the role of known variants in these phase I and phase II enzymes in the efficacy and toxicity of dasatinib [62]. 

Transporters

Like nilotinib, the absorption of dasatinib is mainly passive; it has been shown that the OCT-1, OCT-2, and OCT-3 transporters do not affect dasatinib uptake [2]. Most of its tumor efficacy is influenced by efflux transporters; specifically, ABCB1, ABCG2, ABCC4 (MDR4), and ABCC6 [2]. A review by Eadie et al. revealed that ABCB1 is of more relevance than ABCG2, although ABCG2 does transport dasatinib [52]. Moreover, it has been shown that the inhibition of ABCC6 enhanced the efficacy of dasatinib [2]. Other TKI interactions with ABCC6 have yet to be investigated. It is clear from findings such as this that most of the uncertainty about transporters and TKIs is because not all transporters have been tested. Furthermore, as suggested above, it is not clear where the transporters are acting to create the most impact on tumor cell viability. The interactions between dasatinib and respective transporter proteins are summarized in Table 2.

**Table 2:** Interactions between BCR-ABL tyrosine kinase inhibitors and transporter proteins 'Substrate' indicates that the drug is transported by the protein, 'No interaction' indicates that the drug has been explicitly studied and research has not found there to be any interaction, 'Inhibitor' indicates that the drug inhibits the transport of other drugs. An empty space signifies that the transporter has not been studied or the results have not been reported. ABC: ATP-binding cassette protein; OCT: octamer-binding protein; SLCO1A2: solute carrier organic anion transporter family member 1A2; BCR: breakpoint cluster region; ABL: Abelson

Transporter	Imatinib	Nilotinib	Dasatinib	Bosutinib
ABCB1	Substrate	Substrate	Substrate	Substrate
ABCG2	Inhibitor	Substrate	Substrate	No interaction
ABCC4	Substrate		Substrate	
ABCC6			Substrate	
OCT-1	Substrate	No interaction	No interaction	No interaction
OCT-2		No interaction	No interaction	
SLCO1A2	Unclear			

Additional Considerations Impacting Patient Response

Dasatinib interacts with more kinases, and this may underlie the higher occurrence of adverse events. For example, SRC kinases are important regulators of hematopoiesis, which may explain the higher rates of cytopenias in patients treated with dasatinib compared to imatinib [9]. Dasatinib also inhibits PDGFR-β, but there is little information on how this affects potassium and calcium levels. Dasatinib has been shown to decrease the QT interval [63], but with no clear explanation so far. Instead, most of the research regarding dasatinib toxicity has been focused on the pulmonary system. It was originally thought that SRC inhibition led to pulmonary vascular dysregulation and pulmonary hypertension [59]. However, a review by El-Dabh et al. suggested otherwise, with the authors noting that most patients diagnosed with pulmonary hypertension following dasatinib treatment received other chemotherapeutic agents which may have influenced the results [59]. The mechanism of pulmonary hypertension is unknown but may involve an immune-mediated pathway or off-target inhibition of PDGFR-β. The review by El-Dabh et al. also cited a study by Guignabert et al. which demonstrated that, in rats, pretreatment by dasatinib alone led to an exaggerated response to monocrotaline and chronic hypoxia [59]. Dasatinib increased markers of endothelial dysfunction including soluble ICAM-1, soluble VCAM-1, and soluble E selectin. It also attenuated hypoxic vasoconstriction; and induced pathways implicated in endoplasmic reticulum stress in rats. This may have implications for patients who have variants of these proteins [64]. But more important is the need to resolve the mechanisms of the secondary effects of dasatinib so that genetic studies can be guided by what is already known about imatinib and nilotinib. A further concern for dasatinib is uptake. Due to its pH-dependent solubility, any agents that increase gastric pH may have a major impact on the oral bioavailability of dasatinib, including proton pump inhibitors (PPIs), H2-receptor antagonists (H2RAs), antacids, pentagastrin, and betaine HCl [62]. Very little is known about how genetics may impact this process but, once again, drug interactions also need to be further researched.

Bosutinib

Bosutinib hydrate (trade name Bosulif) is another second-generation TKI. This drug binds to a conformation of ABL1 that is transitional between the active and inactive conformations, making the drug approximately 25-fold more potent than imatinib [65]. The drug also binds to the Src and Abl tyrosine kinases with minimal activity against c-KIT or platelet-derived growth factor receptors [66]. In 2012, bosutinib first received FDA approval for CML therapy in combination with prior TKIs. Then, in 2017, the drug became approved for use as a first-line treatment for newly diagnosed chronic phase CML patients in the United States [59]. As the most recent addition to the arsenal against CML, it is far behind the other TKIs in the research on drug metabolism.

CYP Enzymes

Bosutinib is metabolized by CYP3A4, which breaks down the compound into the two major metabolites,oxidized bosutinib (M2) and N-desmethyl bosutinib (M5), and one minor metabolite, bosutinib N-oxide (M6) [67,68]. Although these metabolites are inactive, the metabolism is slow as bosutinib has a long half-life (32-41 hours), indicating that liver metabolism may not be a major factor in the overall activity of bosutinib [69]. Nonetheless, more research is required on whether other cytochromes, glucuronosyltransferases, or monooxygenases modify bosutinib and influence the overall activity.

Transporters

It has been established that ABCB1 is the primary enzyme responsible for bosutinib transport [69]. ABCG2 and OCT-1 have also been investigated but do not appear to have a major impact on bosutinib metabolism [69]. In Japanese patients, polymorphisms of ABCB1, ABCG2, and CYP3A4 did not appear to influence the circulating concentration of bosutinib while polymorphisms of NR1I2 (Nuclear Receptor Subfamily 1 Group I member 2) did [70]. It was observed that individuals with NR1I2 7635G/G or 8055 T/T genotypes had lower Cmin [70]. Information on the NR1I2 genotype may be useful to achieve optimal systemic exposure to bosutinib. NR112 is one of two transcription factors that regulate CYP expression, which begs the question of why this transcription factor influences bosutinib concentration when the CYP enzymes that are regulated by it have minimal impact. Given that this study was conducted in Japanese patients, it may be related to the UGT1A1 enzyme. More research regarding the role of UGT1A1 enzymes is needed to address this.

Additional Considerations Impacting Patient Response

Bosutinib has only minimal off-target effects against PDGFR and c-Kit receptors which likely accounts for its relatively low toxicity profile. It has been established that diarrhea is the major toxicity associated with bosutinib as it is seen in approximately 84% of patients [71]. However, patients very rarely discontinue the drug due to this side effect and it is usually managed with supportive care [7]. Instead, hypertension may be a more relevant toxicity worth investigating further. Cui et al. found that a clinical dose of bosutinib (100 mg/kg) increased systolic blood pressure and impaired endothelium-dependent vasodilation independent of c-Abl [72]. They demonstrated that CYP soluble epoxide hydrolase (CYP-sEH), the enzyme that breaks down arachidonic acid into epoxyeicosatrienoic acid (EET), is what plays a critical role in regulating the bosutinib-impaired vasodilation and increased blood pressure. The authors also found that the sEH inhibitor 1-Trifluoromethoxyphenyl-3-(1-propionylpiperidin-4-yl) urea (TPPU) reversed the effects of bosutinib on hypertension, demonstrating a novel therapeutic approach for handling this side effect. This pathway should be investigated to further quantify how this drug impacts hypertensive patients as its limited off-target effects make it an otherwise excellent choice [72].

Discussion

Today, clinicians have the option to choose from several TKIs due to their wide availability. Thus, an understanding of which pharmacogenetic factors contribute to TKI efficacy is crucial for optimizing treatment for individual patients. Variations in CYP enzymes are a natural pharmacogenetic consideration as they have been shown to influence other classes of drugs [73]. We have summarized what the cumulative research has found regarding which CYP enzymes interact with each of the first- and second-generation TKIs (Table 1). However, the literature is leaning towards the idea that CYP metabolism may not be a major factor in explaining differences between individuals related to the efficacy of imatinib, nilotinib, and bosutinib due to the slow metabolism of these drugs and the fact that most of their administered dose is excreted unchanged [1,2,23]. For the most part, the accumulated data indicates that research should be focused elsewhere to understand how human genetic variation influences the activity of TKIs. However, it should be noted that the study of liver metabolism of TKIs has focused primarily on variants of CYP3A4 with the belief that other enzymes are minor players and no expectation that a person could have more than one defect in metabolism. It would be helpful for laboratories to establish all enzymes that can contribute to the life cycle of TKIs from peak concentrations to trough concentrations and from the initial dose to the ones provided five years later. Then, future studies could examine all those enzymes in each patient to determine how they collectively contribute to the success or failure of treatment. We have provided a list of all of the human variants that are known or could be reasonably predicted to impact the activity of any of the genes discussed in this review (See Appendices).

Another notable finding is the significant effects of UGT1A1 on nilotinib. As it stands, the impact of UGT1A1 polymorphisms on nilotinib efficacy is perhaps the only verified example of a significant genetic effect by liver enzymes. This phase II enzyme should clearly be investigated for other TKIs. UGT1A1 has been shown to play a role in many chemotherapeutic agents [49,50] and it would be useful to understand whether all TKIs are affected or just a few. Other phase I and phase II enzymes should be investigated to rule in or out their roles. A surprising observation is the impact of NR1I2 on bosutinib metabolism in Japanese patients [70]. This occurs despite the fact that NR1I2 regulates the expression of CYP enzymes that supposedly have minimal impact on TKI metabolism. It would be interesting to know if NR1I2 has a similar impact on imatinib, nilotinib, and dasatinib as well as how such effects are propagated. The observation that variants of NR1I2 influenced bosutinib metabolism in Japanese patients could also be further support for a wider investigation of the role of variants of UGT1A1, CYP enzymes, and NR1I2 in this patient population. Those studying the impact of human genetics on TKI metabolism need to cast a wider net.

Dasatinib seems to be an exception to this class of drugs in that it has a short half-life and only the minority of the drug is excreted unchanged [2]. Therefore, further investigation regarding CYP enzymes in the context of dasatinib should be considered. Dasatinib is mainly metabolized by CYP3A4, which brings into play the fact that many other drugs a patient might be taking could influence metabolism. Drug interactions are quite common with CYP3A4 [74]. Do all patients metabolize dasatinib at the same speed or do some co-administered drugs slow the process? Furthermore, additional enzymes, such as CYP3A5, FMO3, and UGT family members, should be investigated more thoroughly to establish how they affect efficacy and toxicity both individually and in combination.

Variation in human transporter proteins has been another major focus in the study of TKI metabolism. The most frequent variants of well-known transporters have been tested with inconsistent results [23]. Better studies are required to establish the role transporters play in setting an effective concentration inside tumor cells. It is perhaps possible to conclude that genetic variation in individual transporters plays a small role in establishing a desired concentration within the bloodstream (Table 2). Since CML is a bloodborne disease, one could conclude that higher blood levels should correlate with greater efficacy. But almost nothing is defined about how each TKI gets into and out of tumor cells. There are hints that this is different for each TKI and that lesser-known transporters may be involved [23,52,54]. More precise study designs are needed. Existing studies do not account for the ubiquity of the expression of these transporters, the ability of many tumors to overexpress these proteins, or the fact that multiple transporters could be acting in concert [23]. Studies conducted in transfected cells in vitro should do a better job of defining what transporters are already operating before adding the manipulated transporter to the equation. Furthermore, most of the studied transporters require ATP for activity and it is not known how much the TKIs, which are ATP analogs, inhibit the activity of the transporters to begin with. The mechanisms by which a mutation might influence transport activity will be quite different for TKIs than for other chemotherapeutic agents. Those studies looking at efficacy within the first few weeks of treatment may be the most informative as the cancer has not had time to adapt to the treatment. Studies should also take into account the possibility that multiple transporters may contribute to the overall flow of TKIs into or out of tumor cells and thus one altered allele makes a small change to the overall flux of the drug. Again, the data collected so far suggests that more comprehensive studies are required, to ask what the combination of transporters accomplishes rather than measuring one, within a system that contains many others that are ignored.

The key to establishing whether genetic variation plays a role in treatment success with TKIs may require further work on auxiliary pathways and off-target binding effects. Established off-target binding sites are summarized in Table 3. Studies looking at the PDGFR or other cellular tyrosine kinases can help explain some of the differences between imatinib, nilotinib, dasatinib, and bosutinib with respect to the side effects. The off-target proteins provide additional targets to investigate the impact of human variation as not all patients experience adverse events. If the adverse events are better understood and can be predicted, this may prove useful in choosing which drug may be best tolerated by the patient in front of you. However, the more useful work is in identifying new targets for future research. Secondary metabolizers like sulfotransferases, methylases, and glutathione S transferases should be evaluated and any protein that might bind ATP should be considered. The study by Cereja et al. identified ATIC, a protein involved in purine biosynthesis, and ARID5B, a protein involved in hematopoietic cell growth and differentiation, as significantly influencing treatment [36]. This opens the door to other genes that might be indicators of response but are not directly involved in the metabolism of TKIs or their mechanism of action. Likewise, the study by Cui et al. on the role of bosutinib in hypertension suggests that prostaglandins may mediate some of the activities associated with the TKIs [72]. Interestingly, prostaglandins play a role in stem cell proliferation and lymphangiogenesis and NR1I2 induces the expression of cytochrome enzymes that can modify prostaglandins [70]. These studies represent completely new places to look for the impact of human variation. Much of the research on TKIs has focused on tumor resistance mechanisms. Mostly, this involves mutations to the BCR-ABL fusion protein, but other mechanisms of resistance are beginning to be considered. Nath et al. suggested that alternative oncogenic pathways may compensate for the loss of BCR-ABL stimulation in TKI-treated patients [18]. Ultimately, this argues for a thorough analysis of the tumor prior to selecting therapy as do studies now involving analysis of BCR-ABL resistance mutations [13,15,17]. The data presented here argues for a thorough analysis of the patient as well, since it is well known that some individuals do not benefit from TKIs upon their initial exposure before resistance can emerge [10]. There are likely to be additional properties of the tumor and the patient that require further investigation. A thorough analysis of a patient with CML requires that all genes be considered, and all variants of each gene be identified to best inform the oncologist considering the use of a TKI.

**Table 3:** Off-target binding sites known for the BCR-ABL tyrosine kinase inhibitors PDGFR: platelet-derived growth factors; c-KIT: receptor tyrosine kinase; DDR-1,-2: discoidin domain receptors -1, -2; Src kinase: non-receptor tyrosine kinase; EPHX2: epoxide hydrolase 2; TKI: tyrosine kinase inhibitor; BCR: breakpoint cluster region; ABL: Abelson

Drug	Non-targeted proteins that bind the TKIs
Imatinib	PDGFR-α, PDGFR-β, c-KIT
Nilotinib	PDGFR-α, PDGFR-β, c-KIT, DDR-1, DDR-2
Dasatinib	PDGFR, c-KIT, SRC kinases (Src, Fyn, Yes, Lck, Hck, Blk, Fgr, Lyn, and Yrk), Ephrin
Bosutinib	SRC kinases (Src, Fyn, Yes, Lck, Hck, Blk, Fgr, Lyn, and Yrk), EPHX2

## Conclusions

While TKIs work for many people with CML, they do not work for everyone. Some failures occur because of tumor-derived mutations in the BCR-ABL target, which has spurred the development of second-generation TKIs. However, other reasons for treatment failure are likely related to the patient. Research into genetic factors that influence the impact of TKI treatment has a long way to go. Although most TKIs have long half-lives and are mostly excreted unchanged, research on the liver metabolism of TKIs seems incomplete. One example is that nilotinib is known to be modified by UGT1A1, but other TKIs have not been similarly examined. Research into the transporters that influence the intracellular concentration of the TKIs is also incomplete. This is also true for secondary targets that may underlie potential toxicities. Research has focused on the most normally relevant liver enzymes with little progress toward explaining why many patients do not benefit from treatment with TKIs. It is time to investigate other mechanisms that impact the patient experience.

## Appendices

**Table 4:** List of all of the human variants that could reasonably be predicted to impact the activity of any of the genes discussed in this review

Gene	Chromosome	Position	rsIDs	Protein Consequence	Transcript Consequence
ABCB1	7	87229501	rs2214102	intronic	c.-1A>G
ABCB1	7	87168659	rs1156870776	p.Gly774ValfsTer28	c.2321delG
ABCB1	7	87173518	rs576829142	p.Val713LeufsTer8	c.2137delG
ABCB1	7	87196174	rs757098854	p.His153SerfsTer12	c.456dupT
ABCB1	7	87196165	rs756530200	p.Met156Ter	c.464_465dupTA
ABCB1	7	87170673	rs750826229	p.Gln773ArgfsTer29	c.2318delA
ABCB1	7	87160696	rs1348569323	p.Ile867SerfsTer4	c.2598delC
ABCB1	7	87214844	rs756121403	p.Ser90Ter	c.269delC
ABCB1	7	87173487	rs1183996933	p.Gly723AlafsTer8	c.2168delG
ABCB1	7	87148698	rs267601612	p.Phe957SerfsTer13	c.2870delT
ABCB1	7	87170745	rs950456921	p.Arg749HisfsTer12	c.2246delG
ABCB1	7	87133738	rs1188387982	p.Arg1222GlufsTer8	c.3663delC
ABCB1	7	87150095	rs148718120	p.Tyr928CysfsTer9	c.2772_2782dupGCAGGTACCAT
ABCB1	7	87133721	rs1237962780	p.Cys1227HisfsTer62	c.3679_3680delTG
ABCB1	7	87229456	rs781599319	p.Lys14del	c.42_44delGAA
ABCB1	7	87160656	rs1292437074	p.Leu879del	c.2636_2638delTGT
ABCB1	7	87170759	rs756214997	p.Asp744del	c.2230_2232delGAT
ABCB1	7	87183081	rs766673892	p.Gln330_Val331del	c.989_994delAAGTAC
ABCB1	7	87183191	rs772281490	p.Ile293_Ala295dup	c.876_884dupTATTACAGC
ABCB1	7	87174066	rs2235046	intronic	c.2064+73A>G
ABCB1	7	87180198	rs10276036	intronic	c.1000-44G>A
ABCB1	7	87179143	rs2235033	intronic	c.1554+24T>C
ABCB1	7	87178626	rs2235013	intronic	c.1725+38G>A
ABCB1	7	87160561	rs2032583	intronic	c.2685+49T>C
ABCB1	7	87179443	rs2032588	intronic	c.1350+44C>T
ABCB1	7	87174390	rs28381920	intronic	c.1888-75C>T
ABCB1	7	87225046	rs2235074	intronic	c.117+36C>T
ABCB1	7	87138847	rs61040122	intronic	c.3283-50C>T
ABCB1	7	87225173	rs192365005	intronic	c.69-43T>C
ABCB1	7	87179457	rs377260637	intronic	c.1350+30C>T
ABCB1	7	87174327	rs547093769	intronic	c.1888-12T>C
ABCB1	7	87138858	rs949343048	intronic	c.3283-61T>C
ABCB1	7	87196066	rs752103700	intronic	c.530+35G>A
ABCB1	7	87174109	rs756910860	intronic	c.2064+30A>T
ABCB1	7	87229395	rs773338757	intronic	c.68+38G>A
ABCB1	7	87144515	rs141755720	intronic	c.3282+32G>T
ABCB1	7	87173427	rs1291526534	intronic	c.2211+18T>A
ABCB1	7	87215021	rs754592414	intronic	c.118-25G>A
ABCB1	7	87190530	rs780878256	intronic	c.827+49C>T
ABCB1	7	87229408	rs762925936	intronic	c.68+25A>G
ABCB1	7	87180183	rs766402999	intronic	c.1000-29G>A
ABCB1	7	87199462	rs1370607856	intronic	c.338+26G>T
ABCB1	7	87138573	rs762374613	intronic	c.3489+18T>C
ABCB1	7	87509195	rs2235048	intronic	c.3489+80C>T
ABCB1	7	87532204	rs11983225	intronic	c.2482-707A>G
ABCB1	7	87540040	rs12720067	intronic	c.2482-695G>A
ABCB1	7	87160618	rs2032582	p.Ser893Ala	c.2677T>G
ABCB1	7	87229440	rs9282564	p.Asn21Asp	c.61A>G
ABCB1	16	16173232	rs45511401	p.Gly671Val	c.2012G>T
ABCB1	7	87160618	rs2032582	p.Ser893Thr	c.2677T>A
ABCB1	7	87179809	rs2229109	p.Ser400Asn	c.1199G>A
ABCB1	16	16177275	rs4148356	p.Arg723Gln	c.2168G>A
ABCB1	7	87138760	rs55852620	p.Gln1107Pro	c.3320A>C
ABCB1	7	87138659	rs2229107	p.Ser1141Thr	c.3421T>A
ABCB1	16	16208683	rs13337489	p.Cys1047Ser	c.3140G>C
ABCB1	7	87133651	rs28364274	p.Val1251Ile	c.3751G>A
ABCB1	7	87190625	rs36008564	p.Ile261Val	c.781A>G
ABCB1	7	87195540	rs60419673	p.Asn183Ser	c.548A>G
ABCB1	16	16165572	rs112282109	p.Arg633Gln	c.1898G>A
ABCB1	16	16215877	rs28706727	p.Val1146Ile	c.3436G>A
ABCB1	16	16208889	rs200039403	p.Ala1116Thr	c.3346G>A
ABCB1	16	16208928	rs74985930	p.Val1129Ile	c.3385G>A
ABCB1	16	16130339	rs200485505	p.Arg230Trp	c.688C>T
ABCB1	16	16208739	rs199773531	p.Arg1066Trp	c.3196C>T
ABCB1	16	16208851	rs182118381	p.Gly1103Asp	c.3308G>A
ABCB1	16	16200716	rs201798499	p.Ala953Pro	c.2857G>C
ABCB1	16	16130340	rs8187848	p.Arg230Gln	c.689G>A
ABCB1	7	87174198	rs35023033	p.Arg669Cys	c.2005C>T
ABCB1	7	87196129	rs61122623	p.Val168Ile	c.502G>A
ABCB1	16	16230363	rs183032276	p.Arg1385Gln	c.4154G>A
ABCB1	16	16232369	rs199815778	p.Val1481Ile	c.4441G>A
ABCB1	16	16225727	rs201533167	p.Arg1301Cys	c.3901C>T
ABCB1	16	16205379	rs200760311	p.Gly1007Arg	c.3019G>A
ABCB1	16	16101842	rs41494447	p.Thr73Ile	c.218C>T
ABCB1	16	16101809	rs187769078	p.Arg62Gln	c.185G>A
ABCB1	7	87196200	rs61607171	p.Ile144Thr	c.431T>C
ABCB1	16	16103682	rs8187844	p.Ser92Phe	c.275C>T
ABCB1	7	87174218	rs35657960	p.Leu662Arg	c.1985T>G
ABCB1	7	87214848	rs35810889	p.Met89Thr	c.266T>C
ABCB1	7	87160789	rs28381967	p.Ile836Val	c.2506A>G
ABCB1	16	16205325	rs35529209	p.Ala989Thr	c.2965G>A
ABCB1	7	87135347	rs59241388	p.Lys1168Glu	c.3502A>G
ABCB1	7	87179256	rs142600685	p.Arg489Cys	c.1465C>T
ABCB1	16	16170185	rs539166124	p.Gly639Trp	c.1915G>T
ABCB1	7	87144567	rs57521326	p.Asp1088Asn	c.3262G>A
ABCB1	16	16208799	rs200199506	p.Met1086Val	c.3256A>G
ABCB1	16	16142015	rs77428024	p.Asn412Ser	c.1235A>G
ABCB1	16	16225713	rs201761081	p.Arg1296Gln	c.3887G>A
ABCB1	16	16138351	rs199675371	p.Pro285Leu	c.854C>T
ABCB1	16	16101826	rs201384888	p.Thr68Ala	c.202A>G
ABCB1	7	87138758	rs35730308	p.Trp1108Arg	c.3322T>C
ABCB1	16	16146666	rs148042527	p.Thr489Met	c.1466C>T
ABCB1	16	16230476	rs202167272	p.Cys1423Ser	c.4267T>A
ABCB1	7	87150102	rs201316099	p.Val926Ile	c.2776G>A
ABCB1	16	16130342	rs201704029	p.Gly231Cys	c.691G>T
ABCB1	7	87180049	rs199766539	p.Ile369Val	c.1105A>G
ABCB1	16	16200674	rs530286901	p.Ala939Pro	c.2815G>C
ABCB1	7	87175288	rs56107566	p.Arg593His	c.1778G>A
ABCB1	16	16230411	rs8057331	p.Thr1401Met	c.4202C>T
ABCB1	7	87190669	rs201722148	p.Ala246Val	c.737C>T
ABCB1	7	87148696	rs144369247	p.Arg958Gln	c.2873G>A
ABCB1	16	16173256	rs551578363	p.Gln679Pro	c.2036A>C
ABCB1	16	16215931	rs202134920	p.Val1164Ile	c.3490G>A
ABCB1	7	87214970	rs139583955	p.Lys48Asn	c.144G>T
ABCB1	7	87179789	rs140214314	p.Val407Phe	c.1219G>T
ABCB1	16	16142136	rs377285314	p.Ile452Met	c.1356C>G
ABCB1	16	16108480	rs201076225	p.Lys162Glu	c.484A>G
ABCB1	7	87144678	rs28401798	p.Pro1051Ala	c.3151C>G
ABCB1	16	16205236	rs768191257	p.Lys959Arg	c.2876A>G
ABCB1	7	87174266	rs528939709	p.Ser646Ile	c.1937G>T
ABCB1	16	16101793	rs199949505	p.Leu57Val	c.169C>G
ABCB1	16	16138377	rs199742367	p.Asn294His	c.880A>C
ABCB1	7	87174260	rs773525613	p.Ile648Thr	c.1943T>C
ABCB1	16	16138311	rs200194736	p.Pro272Ser	c.814C>T
ABCB1	16	16184331	rs186193767	p.Gly844Ser	c.2530G>A
ABCB1	16	16208860	rs199698664	p.Ile1106Thr	c.3317T>C
ABCB1	16	16218708	rs201585228	p.Ala1218Val	c.3653C>T
ABCB1	16	16225712	rs200922662	p.Arg1296Trp	c.3886C>T
ABCB1	7	87138764	rs148897157	p.Val1106Ile	c.3316G>A
ABCB1	16	16138312	rs376004761	p.Pro272Leu	c.815C>T
ABCB1	16	16225674	rs765498811	p.Pro1283Leu	c.3848C>T
ABCB1	7	87179815	rs144933300	p.His398Leu	c.1193A>T
ABCB1	7	87196110	rs201280497	p.Arg174Gln	c.521G>A
ABCB1	7	87144686	rs756736504	p.Pro1048Leu	c.3143C>T
ABCB1	7	87168600	rs201249149	p.Arg794Gln	c.2381G>A
ABCB1	7	87175304	rs201122883	p.Arg588Cys	c.1762C>T
ABCB1	7	87148655	rs770657790	p.Glu972Lys	c.2914G>A
ABCB1	7	87135302	rs199676098	p.Arg1183Cys	c.3547C>T
ABCB1	7	87225105	rs751270575	p.Pro32Thr	c.94C>A
ABCB1	7	87160648	rs147823195	p.Ala883Thr	c.2647G>A
ABCB1	7	87133714	rs757394498	p.Ile1230Val	c.3688A>G
ABCB1	7	87195430	rs758834776	p.Ala220Thr	c.658G>A
ABCB1	7	87178761	rs1374940464	p.Arg543His	c.1628G>A
ABCB1	7	87178678	rs755085099	p.Val571Met	c.1711G>A
ABCB1	7	87196263	rs759986853	p.Ala123Val	c.368C>T
ABCB1	7	87144552	rs776827328	p.Lys1093Glu	c.3277A>G
ABCB1	7	87144642	rs761914266	p.Gly1063Cys	c.3187G>T
ABCB1	7	87214864	rs764061195	p.Asn84His	c.250A>C
ABCB1	7	87135295	rs764463583	p.Ala1185Asp	c.3554C>A
ABCB1	7	87179517	rs756800740	p.Met440Ile	c.1320G>A
ABCB1	7	87195492	rs1403432068	p.Thr199Ile	c.596C>T
ABCB1	7	87196163	rs753065601	p.Met156Ile	c.468G>A
ABCB1	7	87195559	rs776202744	intronic	c.531-2A>G
ABCB1	7	87179371	rs1237771217	intronic	c.1351-1G>T
ABCB1	7	87225081	rs1306209813	intronic	c.117+1G>A
ABCB1	7	87168583	rs764778797	intronic	c.2397+1G>C
ABCB1	7	87148641	rs1027997182	intronic	c.2927+1G>A
ABCB1	7	87148641	rs1027997182	intronic	c.2927+1G>T
ABCB1	7	87196100		intronic	c.530+1G>C
ABCB1	7	87229504	rs34834348	intronic	c.-4C>T
ABCB1	7	87165766	rs34350171	intronic	c.2481+8C>G
ABCB1	7	87175175	rs201859423	intronic	c.1887+4T>G
ABCB1	7	87138591	rs766547835	p.Asn1163Asn	c.3489T>C
ABCB1	7	87168581	rs28381943	intronic	c.2397+3A>G
ABCB1	7	87168668	rs200323156	intronic	c.2320-7C>G
ABCB1	7	87133769	rs202003598	intronic	c.3637-4A>T
ABCB1	7	87145988	rs747827053	intronic	c.2928-7T>A
ABCB1	7	87145985	rs1302731388	intronic	c.2928-4C>T
ABCB1	7	87165855	rs201142514	p.Asp800Asp	c.2400T>C
ABCB1	7	87179617	rs1394086104	intronic	c.1225-5G>A
ABCB1	7	87175175	rs201859423	intronic	c.1887+4T>C
ABCB1	7	87179377	rs1276520445	intronic	c.1351-7A>T
ABCB1	7	87178660	rs751777121	intronic	c.1725+4A>T
ABCB1	7	87180157	rs375724186	intronic	c.1000-3C>G
ABCB1	7	87168579	rs1206141026	intronic	c.2397+5G>A
ABCB1	7	87173438	rs1249718120	intronic	c.2211+3_2211+6delAAGT
ABCB1	7	87178840	rs201728513	intronic	c.1555-6T>C
ABCB1	7	87174132	rs200120627	intronic	c.2064+7A>T
ABCB1	7	87150190	rs532094830	p.Ile896Ile	c.2688C>T
ABCB1	7	87145825	rs2235044	p.Pro1028Pro	c.3084G>A
ABCB1	7	87165862	rs763717036	intronic	c.2398-5C>T
ABCB1	7	87170667	rs756346795	intronic	c.2319+6T>C
ABCB1	7	87225074	rs761829302	intronic	c.117+8T>C
ABCB1	7	87168580	rs201202639	intronic	c.2397+4T>C
ABCB1	7	87135205	rs780099939	intronic	c.3636+8T>C
ABCB1	7	87178660	rs751777121	intronic	c.1725+4A>G
ABCB1	7	87168576	rs187910292	intronic	c.2397+8T>A
ABCB1	7	87168665	rs199577971	intronic	c.2320-4A>G
ABCB1	7	87229510	rs771041692	intronic	c.-6-4T>A
ABCB1	7	87170668	rs777842457	intronic	c.2319+5A>G
ABCB1	7	87190581	rs775665658	p.Glu275Glu	c.825A>G
ABCB1	7	87170783	rs1319884327	intronic	c.2212-3C>T
ABCB1	7	87173445	rs201810761	p.Gly737Gly	c.2211G>C
ABCB1	7	87165769	rs768893111	intronic	c.2481+5G>A
ABCB1	7	87199483	rs200469824	intronic	c.338+5T>C
ABCB1	7	87165861	rs1372092073	intronic	c.2398-4A>G
ABCB1	7	87215004	rs34455904	intronic	c.118-8C>T
ABCB1	7	87215004	rs34455904	intronic	c.118-8C>G
ABCB1	7	87160817	rs775305519	intronic	c.2482-5delT
ABCB1	7	87160818	rs1437747989	intronic	c.2482-5T>G
ABCB1	7	87174320	rs750154739	intronic	c.1888-5T>G
ABCB1	7	87160816	rs752216494	intronic	c.2482-8_2482-4dupTTTTG
ABCB1	7	87160816	rs752216494	intronic	c.2482-3C>T
ABCB1	7	87225134	rs905876915	intronic	c.69-4G>T
ABCB1	7	87179780	rs1358557531	intronic	c.1224+4C>T
ABCB1	7	87179779	rs376497843	intronic	c.1224+5A>T
ABCB1	7	87180160	rs1490800054	intronic	c.1000-6C>T
ABCB1	7	87179901	rs745886322	intronic	c.1114-7T>C
ABCB1	7	87173599	rs776875893	intronic	c.2065-8T>C
ABCB1	7	87214823		intronic	c.286+5G>C
ABCB1	7	87145989	rs1213211157	intronic	c.2928-8C>G
ABCB1	7	87214825	rs750426123	intronic	c.286+2dupT
ABCB1	7	87150200	rs750187241	intronic	c.2686-8T>C
ABCB1	7	87195561	rs761393555	intronic	c.531-4T>C
ABCB1	7	87196295	rs764536487	intronic	c.339-3C>A
ABCB1	7	87145985	rs1302731388	intronic	c.2928-4C>G
ABCB1	7	87180152	rs200049706	p.Val334Val	c.1002A>T
ABCB1	7	87168666	rs1317210928	intronic	c.2320-8_2320-6delGCC
ABCB1	7	87168667	rs1241797652	intronic	c.2320-6C>G
ABCB1	7	87148789	rs746958883	intronic	c.2787-8delC
ABCB1	7	87190573	rs534095506	intronic	c.827+6A>C
ABCB1	7	87196292	rs1313646996	p.Arg113Arg	c.339G>A
ABCB1	7	87170787	rs766394090	intronic	c.2212-8delT
ABCB1	7	87199490	rs1468733075	p.Thr112Thr	c.336C>G
ABCB1	7	87199490	rs1468733075	p.Thr112Thr	c.336C>T
ABCB1	7	87148635		intronic	c.2927+7A>T
ABCB1	7	87133771	rs1427356827	intronic	c.3637-6G>T
ABCB1	7	87229511	rs774399781	intronic	c.-6-5T>C
ABCB1	7	87183248	rs1373986918	p.Arg276Arg	c.828G>A
ABCB1	7	87173445	rs201810761	p.Gly737Gly	c.2211G>A
ABCB1	7	87133763	rs143356931	p.Val1213Val	c.3639T>C
ABCB1	7	87173440	rs1292350917	intronic	c.2211+5G>A
ABCB1	7	87144752	rs1297809094	intronic	c.3085-8C>T
ABCB1	7	87196094	rs1343904141	intronic	c.530+7A>G
ABCB1	7	87229504	rs34834348	intronic	c.-4C>G
ABCB1	7	87144749	rs1227228021	intronic	c.3085-5T>G
ABCB1	7	87145817	rs1262501726	intronic	c.3084+8T>A
ABCB1	7	87175179	rs1379886427	p.Gln629Gln	c.1887G>A
ABCB1	7	87183069	rs59787582	intronic	c.999+8G>T
ABCB1	7	87183069	rs59787582	intronic	c.999+8G>A
ABCB1	7	87196103	rs1223174556	p.Thr176Thr	c.528A>G
ABCB1	7	87165770	rs776548654	intronic	c.2481+4C>T
ABCB1	7	87135209	rs1003507978	intronic	c.3636+4A>G
ABCB1	7	87174139	rs1239682305	p.Leu688Leu	c.2064G>A
ABCB1	7	87179481	rs1237772858	intronic	c.1350+6A>G
ABCB1	7	87179368	rs1384523299	p.Val451Val	c.1353C>G
ABCB1	7	87179163	rs1225827272	intronic	c.1554+4A>C
ABCB1	7	87195380	rs1258992204	intronic	c.702+6T>C
ABCB1	7	87195386	rs753495660	p.Lys234Lys	c.702G>A
ABCB1	7	87196162	rs1363838319	p.Arg157Ter	c.469C>T
ABCB1	7	87183132	rs1189919755	p.Trp315Ter	c.944G>A
ABCB1	7	87150165	rs759603974	p.Arg905Ter	c.2713C>T
ABCB1	7	87168616	rs1373964332	p.Arg789Ter	c.2365C>T
ABCB1	7	87144690	rs751009298	p.Arg1047Ter	c.3139C>T
ABCB1	7	87190583	rs202097303	p.Glu275Ter	c.823G>T
ABCB1	7	87170747	rs1323297861	p.Arg749Ter	c.2245C>T
ABCB1	7	87138773	rs1470273650	p.Arg1103Ter	c.3307C>T
ABCB1	7	87160731	rs746152564	p.Trp855Ter	c.2564G>A
ABCB1	7	87183245	rs1297328873	p.Tyr277Ter	c.831C>A
ABCB1	7	87190589	rs1222604496	p.Glu273Ter	c.817G>T
ABCB1	7	87138680	rs767536622	p.Gly1134Ter	c.3400G>T
ABCB1	7	87144654	rs1377559224	p.Glu1059Ter	c.3175G>T
ABCB1	7	87196111	rs199808236	p.Arg174Ter	c.520C>T
ABCB1	7	87196223	rs1248102071	p.Trp136Ter	c.408G>A
ABCB1	7	87196237	rs769545558	p.Gln132Ter	c.394C>T
ABCB1	7	87179601	rs1128503	p.Gly412Gly	c.1236T>C
ABCB1	7	87138645	rs1045642	p.Ile1145Ile	c.3435T>C
ABCB1	7	87183095	rs200490161	p.Ser327Ser	c.981T>C
ABCB1	7	87214955	rs115493381	p.Val53Val	c.159G>T
ABCB1	7	87144634	rs368096241	p.Thr1065Thr	c.3195G>A
ABCC4	13	95768242	rs756989444	p.Asn823LysfsTer12	c.2468dupA
ABCC4	13	95899241	rs752281837	p.Phe98LeufsTer5	c.293_294insG
ABCC4	13	95815431	rs1329814822	p.Gly751GlufsTer5	c.2252delG
ABCC4	13	95735398	rs1218796050	p.Glu893ValfsTer49	c.2678_2681delAATC
ABCC4	13	95887038	rs759080265	p.Asn118IlefsTer13	c.353_356delATTA
ABCC4	13	95813552	rs1261526904	p.Arg782SerfsTer12	c.2342_2345dupCAAG
ABCC4	13	95815456	rs750325898	p.Gln742ValfsTer3	c.2224_2227delCAAA
ABCC4	13	95839011	rs771065737	p.Lys497ArgfsTer12	c.1488delG
ABCC4	13	95861796	rs769643713	p.Thr226LeufsTer8	c.676delA
ABCC4	13	95861798	rs773310016	p.Ala224GlyfsTer9	c.671_674delCAGT
ABCC4	13	95813579	rs776335536	p.Val773SerfsTer19	c.2317_2318delGT
ABCC4	13	95813479	rs1262754180	p.Ala808LeufsTer11	c.2418delG
ABCC4	13	95696668	rs1194785037	p.Glu1160GlyfsTer5	c.3479_3480delAA
ABCC4	13	95838965	rs1420551644	p.Ala512GlyfsTer13	c.1534dupG
ABCC4	13	95768211	rs772348869	p.Asp834IlefsTer96	c.2499_2500insATCC
ABCC4	13	95696633	rs1173263398	p.Glu1172IlefsTer6	c.3514_3515delGA
ABCC4	13	95818601	rs765763181	p.Thr616ArgfsTer8	c.1840_1844dupAAGGG
ABCC4	13	95886879	rs1366968924	p.His172LeufsTer2	c.515delA
ABCC4	13	95727755	rs763799346	p.Trp912Ter	c.2736delG
ABCC4	13	95735488	rs576333615	p.Ile864ThrfsTer26	c.2591delT
ABCC4	13	95847168	rs745933667	p.Glu394IlefsTer12	c.1180_1184delGAGAT
ABCC4	13	95886968	rs1231407756	p.Thr142PhefsTer22	c.423_426delGACT
ABCC4	13	95899922	rs1364490926	p.Gln54AlafsTer13	c.159_160insG
ABCC4	13	95840760	rs1158986994	p.Thr434ProfsTer10	c.1295_1299dupCCTTT
ABCC4	13	95899921	rs1184128129	p.Gln54ArgfsTer17	c.160_161insGG
ABCC4	13	95899923	rs779776078	p.Gln54TrpfsTer14	c.158_159insTTGG
ABCC4	13	95839071	rs1375949250	p.Tyr477MetfsTer13	c.1428delC
ABCC4	13	95830259	rs748577121	p.Asn544ProfsTer10	c.1630_1631delAA
ABCC4	13	95695981	rs1270981267	p.Ile1231AsnfsTer11	c.3686_3689dupTAAC
ABCC4	13	95899927	rs142182014	p.Leu48_Asp51del	c.143_154delTGCCAGAAGACC
ABCC4	13	95818540	rs542993841	p.Asn635del	c.1903_1905delAAT
ABCC4	13	95953541	rs1327101223	p.Glu7_Lys9del	c.19_27delGAGGTGAAG
ABCC4	13	95861696	rs1483592279	p.Phe258_Ser259delinsLeu	c.774_776delCTC
ABCC4	13	95953546	rs1275795217	p.Glu7dup	c.20_22dupAGG
ABCC4	13	95899919	rs1422111391	p.Gln54_His55insLeu	c.162_163insTTG
ABCC4	13	95859035	rs2274407	p.Lys304Asn	c.912G>T
ABCC4	13	95863008	rs11568658	p.Gly187Trp	c.559G>T
ABCC4	13	95815415	rs3765534	p.Glu757Lys	c.2269G>A
ABCC4	13	95953517	rs11568681	p.Leu18Ile	c.52C>A
ABCC4	13	95735520	rs11568694	p.Val854Phe	c.2560G>T
ABCC4	13	95705380	rs11568644	p.Thr1142Met	c.3425C>T
ABCC4	13	95735484	rs139970608	p.Ile866Val	c.2596A>G
ABCC4	13	95815454	rs9282570	p.Met744Val	c.2230A>G
ABCC4	13	95727794	rs45504892	p.Val900Leu	c.2698G>T
ABCC4	13	95839008	rs11568669	p.Lys498Glu	c.1492A>G
ABCC4	13	95768231	rs60532299	p.Lys827Arg	c.2480A>G
ABCC4	13	95899303	rs11568689	p.Pro78Ala	c.232C>G
ABCC4	13	95858806	rs139736278	p.Val381Ile	c.1141G>A
ABCC4	13	95715113	rs11568653	p.Val1071Ile	c.3211G>A
ABCC4	13	95863016	rs45454092	p.Met184Thr	c.551T>C
ABCC4	13	95847145	rs11568705	p.Pro403Leu	c.1208C>T
ABCC4	13	95673889	rs544510376	p.Met1306Ile	c.3918G>C
ABCC4	13	95735502	rs45477596	p.Val860Met	c.2578G>A
ABCC4	13	95830299	rs142211148	p.Arg531Gln	c.1592G>A
ABCC4	13	95863004	rs536107894	p.Lys188Thr	c.563A>C
ABCC4	13	95727686	rs148067777	p.Glu936Gln	c.2806G>C
ABCC4	13	95860088	rs11568684	p.Lys293Glu	c.877A>G
ABCC4	13	95899317	rs556194310	p.Asn73Ser	c.218A>G
ABCC4	13	95858953	rs377022090	p.Val332Met	c.994G>A
ABCC4	13	95724031	rs201038632	p.Pro1032Arg	c.3095C>G
ABCC4	13	95818571	rs11568699	p.Ile625Met	c.1875A>G
ABCC4	13	95839122	rs747675034	p.Gly460Arg	c.1378G>A
ABCC4	13	95839128	rs200858900	p.Val458Met	c.1372G>A
ABCC4	13	95813535	rs778266348	p.Tyr788Cys	c.2363A>G
ABCC4	13	95839002	rs145886106	p.Glu500Lys	c.1498G>A
ABCC4	13	95839040	rs11568668	p.Gly487Glu	c.1460G>A
ABCC4	13	95813572	rs146708960	p.Val776Ile	c.2326G>A
ABCC4	13	95860123	rs751301974	p.Thr281Ile	c.842C>T
ABCC4	13	95813560	rs201512856	p.Ile780Leu	c.2338A>C
ABCC4	13	95847161	rs141417140	p.Arg398Cys	c.1192C>T
ABCC4	13	95735396	rs751855343	p.Thr895Ile	c.2684C>T
ABCC4	13	95899972	rs767254775	p.Arg37Gln	c.110G>A
ABCC4	13	95686914	rs138512563	p.Met1272Thr	c.3815T>C
ABCC4	13	95861852	rs746407053	intronic	c.622-1G>A
ABCC4	13	95830048	rs1234801214	intronic	c.1641-1G>C
ABCC4	13	95839148	rs1389754713	intronic	c.1354-2A>G
ABCC4	13	95822884	rs747203143	intronic	c.1728-2A>C
ABCC4	13	95696694	rs757959172	intronic	c.3457-2A>G
ABCC4	13	95816774	rs143187305	intronic	c.2035-2A>G
ABCC4	13	95858784		intronic	c.1161+2T>G
ABCC4	13	95953494	rs1173992826	intronic	c.74+1G>C
ABCC4	13	95847088	rs1343196350	intronic	c.1263+2T>C
ABCC4	13	95886863	rs768195762	intronic	c.531+1G>A
ABCC4	13	95886862	rs762607666	intronic	c.531+2T>C
ABCC4	13	95899896	rs780917947	intronic	c.185+1G>T
ABCC4	13	95727685	rs778961775	intronic	c.2806+1G>A
ABCC4	13	95727684	rs1322253010	intronic	c.2806+2T>C
ABCC4	13	95705348	rs1219879367	intronic	c.3456+1G>T
ABCC4	13	95673939	rs9524765	intronic	c.3871-3T>C
ABCC4	13	95899354	rs4148437	intronic	c.186-5T>C
ABCC4	13	95858778	rs11568702	intronic	c.1161+8T>A
ABCC4	13	95899890	rs11568700	intronic	c.185+7C>T
ABCC4	13	95818628	rs11568696	intronic	c.1825-7A>G
ABCC4	13	95847194	rs2274404	intronic	c.1162-3T>C
ABCC4	13	95748440	rs767716189	intronic	c.2536-7T>G
ABCC4	13	95715119	rs568004891	intronic	c.3211-6C>T
ABCC4	13	95861854	rs564629159	intronic	c.622-3A>C
ABCC4	13	95815474	rs537579667	intronic	c.2214-4A>G
ABCC4	13	95899889	rs763815541	intronic	c.185+8G>A
ABCC4	13	95727808	rs775263883	intronic	c.2687-3C>T
ABCC4	13	95838955	rs149813736	p.Lys515Lys	c.1545G>A
ABCC4	13	95725562	rs775696518	intronic	c.2918-4G>A
ABCC4	13	95735552	rs200283979	intronic	c.2536-8T>G
ABCC4	13	95673940	rs752575637	intronic	c.3871-4C>T
ABCC4	13	95726460	rs774287710	intronic	c.2917+8C>T
ABCC4	13	95686853	rs375925660	intronic	c.3870+6C>T
ABCC4	13	95725563	rs201886144	intronic	c.2918-5C>T
ABCC4	13	95899892	rs374824405	intronic	c.185+5G>C
ABCC4	13	95748437	rs1235050812	intronic	c.2536-4A>G
ABCC4	13	95822886	rs1292201792	intronic	c.1728-4A>G
ABCC4	13	95862941	rs372813116	intronic	c.621+5G>A
ABCC4	13	95768254	rs1281109486	p.Gly819Gly	c.2457A>T
ABCC4	13	95829954	rs541296492	intronic	c.1727+7G>A
ABCC4	13	95863043	rs762577453	intronic	c.532-8T>C
ABCC4	13	95830351	rs773364470	intronic	c.1546-6A>G
ABCC4	13	95818624	rs377056175	intronic	c.1825-3T>C
ABCC4	13	95686996	rs775336450	intronic	c.3736-3C>T
ABCC4	13	95686852	rs754094423	intronic	c.3870+7G>A
ABCC4	13	95725564	rs764107411	intronic	c.2918-6A>G
ABCC4	13	95715119	rs568004891	intronic	c.3211-6C>G
ABCC4	13	95768259	rs758407958	intronic	c.2456-4G>A
ABCC4	13	95815856	rs779133056	intronic	c.2213+8A>G
ABCC4	13	95768170	rs746617615	intronic	c.2535+6G>A
ABCC4	13	95686998	rs748745765	intronic	c.3736-5A>C
ABCC4	13	95726586	rs748169626	intronic	c.2807-8A>C
ABCC4	13	95899353	rs766357757	intronic	c.186-4G>A
ABCC4	13	95768172	rs780841469	intronic	c.2535+4A>G
ABCC4	13	95686854	rs779070576	intronic	c.3870+5G>A
ABCC4	13	95899222	rs756239972	intronic	c.306+7G>T
ABCC4	13	95725452	rs771562574	intronic	c.3018+6A>G
ABCC4	13	95953489	rs760576861	intronic	c.74+6T>C
ABCC4	13	95899353	rs766357757	intronic	c.186-4G>T
ABCC4	13	95768171	rs757008496	intronic	c.2535+5C>T
ABCC4	13	95727811	rs1422067931	intronic	c.2687-6C>T
ABCC4	13	95735386	rs1359308934	intronic	c.2686+8T>C
ABCC4	13	95859041	rs752550546	intronic	c.912-6T>C
ABCC4	13	95816627	rs758581804	intronic	c.2175+5A>G
ABCC4	13	95830353	rs766662216	intronic	c.1546-8C>T
ABCC4	13	95830351	rs773364470	intronic	c.1546-6A>C
ABCC4	13	95830349	rs565835390	intronic	c.1546-4A>G
ABCC4	13	95839153	rs763069009	intronic	c.1354-7G>A
ABCC4	13	95862939	rs1182465176	intronic	c.621+7T>C
ABCC4	13	95723908	rs763827501	intronic	c.3210+8T>G
ABCC4	13	95723911	rs778754779	intronic	c.3210+5G>A
ABCC4	13	95815475	rs1425549502	intronic	c.2214-5C>A
ABCC4	13	95815476	rs759160545	intronic	c.2214-6C>T
ABCC4	13	95839152	rs1428102913	intronic	c.1354-6T>C
ABCC4	13	95839144	rs775989271	p.Ser452Ser	c.1356A>G
ABCC4	13	95861859	rs201752295	intronic	c.622-8C>A
ABCC4	13	95822886	rs1292201792	intronic	c.1728-4A>T
ABCC4	13	95899356	rs759326754	intronic	c.186-7A>G
ABCC4	13	95815473	rs765881798	intronic	c.2214-3T>C
ABCC4	13	95840804	rs1347100599	intronic	c.1264-8A>G
ABCC4	13	95899349	rs761045575	p.Gly62Gly	c.186G>T
ABCC4	13	95725565	rs761621325	intronic	c.2918-7T>C
ABCC4	13	95829956	rs1317226971	intronic	c.1727+5G>A
ABCC4	13	95768170	rs746617615	intronic	c.2535+2_2535+5dupTAAC
ABCC4	13	95726464	rs757709626	intronic	c.2917+4A>G
ABCC4	13	95726465	rs1236573472	intronic	c.2917+3A>G
ABCC4	13	95768168	rs937908780	intronic	c.2535+8T>C
ABCC4	13	95830050	rs781458039	intronic	c.1641-3T>C
ABCC4	13	95838947	rs1483250399	intronic	c.1545+8A>T
ABCC4	13	95838950	rs1199449581	intronic	c.1545+5G>A
ABCC4	13	95686856	rs1354916866	intronic	c.3870+3G>A
ABCC4	13	95816780	rs372203671	intronic	c.2035-8C>T
ABCC4	13	95816780	rs372203671	intronic	c.2035-8C>G
ABCC4	13	95860183	rs1254557119	intronic	c.786-4T>G
ABCC4	13	95714952	rs1440285344	intronic	c.3366+6C>G
ABCC4	13	95815377	rs1469098070	p.Ser769Ser	c.2307A>C
ABCC4	13	95861681	rs1347486214	intronic	c.785+7G>C
ABCC4	13	95714955	rs1183571004	intronic	c.3366+3A>G
ABCC4	13	95696046	rs755932697	intronic	c.3630-5T>C
ABCC4	13	95727813	rs749136061	intronic	c.2687-8T>C
ABCC4	13	95735387	rs1465245162	intronic	c.2686+7A>G
ABCC4	13	95725454	rs1442962196	intronic	c.3018+4A>G
ABCC4	13	95899893	rs1349449035	intronic	c.185+4A>C
ABCC4	13	95818407	rs749142224	intronic	c.2034+5G>A
ABCC4	13	95859043	rs758430364	intronic	c.912-8T>C
ABCC4	13	95727678	rs755372606	intronic	c.2806+8T>A
ABCC4	13	95727680	rs1387875647	intronic	c.2806+6T>A
ABCC4	13	95859040	rs1211772401	intronic	c.912-7_912-6delCT
ABCC4	13	95727687	rs1278418528	p.Ser935Ser	c.2805A>G
ABCC4	13	95830348	rs767919697	intronic	c.1546-3C>T
ABCC4	13	95705343	rs755408362	intronic	c.3456+6T>G
ABCC4	13	95840702	rs773348433	intronic	c.1353+5G>C
ABCC4	13	95840702	rs773348433	intronic	c.1353+5G>A
ABCC4	13	95748408	rs191454929	p.Trp854Ter	c.2561G>A
ABCC4	13	95886896	rs529536678	p.Arg167Ter	c.499C>T
ABCC4	13	95715028	rs1267280711	p.Trp1099Ter	c.3296G>A
ABCC4	13	95815875	rs1273279011	p.Trp734Ter	c.2202G>A
ABCC4	13	95673867	rs760302007	p.Gln1314Ter	c.3940C>T
ABCC4	13	95725484	rs149024948	p.Arg998Ter	c.2992C>T
ABCC4	13	95858797	rs1159726645	p.Arg384Ter	c.1150C>T
ABCC4	13	95887038	rs759080265	p.Tyr119Ter	c.356dupA
ABCC4	13	95705364	rs778104118	p.Trp1147Ter	c.3441G>A
ABCC4	13	95735442	rs1238097822	p.Arg880Ter	c.2638C>T
ABCC4	13	95899236	rs1217879718	p.Leu100Ter	c.299T>G
ABCC4	13	95727757	rs1163159139	p.Trp912Ter	c.2735G>A
ABCC4	13	95900007	rs1454956307	p.Trp25Ter	c.75G>A
ABCC4	13	95900004	rs765931057	p.Trp26Ter	c.78G>A
ABCC4	13	95886917	rs1261399221	p.Gln160Ter	c.478C>T
ABCC4	13	95818462	rs1325758333	p.Gln662Ter	c.1984C>T
ABCC4	13	95818466	rs1317110400	p.Trp660Ter	c.1980G>A
ABCC4	13	95673831	rs769943373	p.Ter1326ArgextTer15	c.3976T>C
ABCG2	4	89060950	rs1218526134	p.Ile63TyrfsTer54	c.187_197delATATTATCGAA
ABCG2	4	89039309	rs780593948	p.Leu264HisfsTer14	c.791_792delTT
ABCG2	4	89018685	rs745473062	p.Met523TrpfsTer5	c.1566delC
ABCG2	4	89080372	rs1433026292	p.Gln7SerfsTer2	c.19delC
ABCG2	4	89052270	rs150450599	p.Asn158LysfsTer5	c.473dupA
ABCG2	4	89052266	rs200727306	p.Glu159AspfsTer10	c.477delA
ABCG2	4	89034584	rs780334268	p.Gly355ValfsTer42	c.1064delG
ABCG2	4	89020571	rs769679787	p.Arg465SerfsTer12	c.1395_1396delAG
ABCG2	4	89022417	rs140681134	p.Ser441ArgfsTer14	c.1321_1331delAGTGTTTCAGC
ABCG2	4	89018723	rs780244322	p.Met510CysfsTer5	c.1528delA
ABCG2	4	89028359	rs759222919	p.Asn418LysfsTer2	c.1253dupA
ABCG2	4	89042912	rs3116439	p.Gly188GlufsTer9	c.563delG
ABCG2	4	89061099	rs1313475538	p.Asn16LysfsTer13	c.48delC
ABCG2	4	89034661	rs781465213	p.Ile329ArgfsTer19	c.986_987delTA
ABCG2	4	89034617	rs751659612	p.Glu344GlyfsTer53	c.1031delA
ABCG2	4	89022450	rs982103317	p.Phe432Ter	c.1295_1298delTCCT
ABCG2	4	89034536	rs387906869	p.Thr371LeufsTer20	c.1111_1112delAC
ABCG2	4	89015763	rs758560040	p.Asn596GlnfsTer9	c.1785dupC
ABCG2	4	89016754	rs1281244555	p.Ser552PhefsTer33	c.1654dupT
ABCG2	4	89052269	rs769012528	p.Asn158LysfsTer11	c.474delC
ABCG2	4	89013393	rs1232606613	p.Tyr654IlefsTer10	c.1960delT
ABCG2	4	89042932	rs1212832973	p.Gln181HisfsTer16	c.543delG
ABCG2	4	89061150	rs1429173245	p.Leu17ProfsTer6	c.50_51delTC
ABCG2	4	89042925	rs762937915	p.Arg184LeufsTer15	c.550_551insTAAAT
ABCG2	4	89020542	rs1274428653	p.Leu475PhefsTer7	c.1425delA
ABCG2	4	89036126	rs1381714945	p.Asn308GlufsTer11	c.922_925delAACA
ABCG2	4	89018736	rs958849526	p.Phe506SerfsTer4	c.1515delC
ABCG2	4	89052999	rs1185751722	p.Pro112ArgfsTer19	c.333delA
ABCG2	4	89061115	rs768166927	p.Pro11GlnfsTer18	c.32delC
ABCG2	4	89061088	rs1168443804	p.Ala22ArgfsTer6	c.59dupT
ABCG2	4	89036173		p.Phe293LeufsTer8	c.875_878dupACTT
ABCG2	4	89039297	rs34678167	p.Phe266SerfsTer10	c.797_804delTCCACGGG
ABCG2	4	89061059	rs947797678	p.Ala30HisfsTer8	c.88delG
ABCG2	4	89052323	rs2231142	p.Gln141ThrfsTer16	c.420dupA
ABCG2	4	89052320	rs1228455521	p.Gln141HisfsTer12	c.423delG
ABCG2	4	89042849	rs766988282	p.Leu209TrpfsTer8	c.626delT
ABCG2	4	89018646	rs759785146	p.Val536CysfsTer49	c.1605dupT
ABCG2	4	89034609	rs748641162	p.Ala347LeufsTer50	c.1039delG
ABCG2	4	89053747	rs1212687042	p.Thr82HisfsTer39	c.243dupC
ABCG2	4	89034505	rs1409635767	p.Lys382GlnfsTer10	c.1143dupC
ABCG2	4	89022439	rs747309767	p.Gln437SerfsTer14	c.1309delC
ABCG2	4	89034567	rs758928764	p.Lys360del	c.1079_1081delAGA
ABCG2	4	89034528	rs762248204	p.Phe373del	c.1118_1120delTCT
ABCG2	4	89036167	rs745365095	p.Phe294del	c.882_884delCTT
ABCG2	4	89039380	rs377457910	p.Phe240del	c.719_721delTCT
ABCG2	4	89052354	rs1052454710	p.Val130del	c.387_389delTGT
ABCG2	4	89034627	rs755318857	p.Ser340del	c.1019_1021delCCT
ABCG2	4	89053025	rs749074058	p.Leu102del	c.305_307delTAT
ABCG2	4	89018721	rs1258482042	p.Met510del	c.1528_1530delATG
ABCG2	4	89053049	rs757240516	p.Val92_Ala94del	c.275_283delTCTTAGCTG
ABCG2	4	89022389	rs1441480899	p.Lys453del	c.1357_1359delAAG
ABCG2	4	89034567	rs758928764	p.Lys360dup	c.1079_1081dupAGA
ABCG2	4	89052323	rs2231142	p.Gln141Lys	c.421C>A
ABCG2	4	89042946	rs531831004	intronic	c.532-2A>G
ABCG2	4	89015812	rs1488051122	intronic	c.1738-1G>A
ABCG2	4	89036211	rs1235110117	intronic	c.842-1G>A
ABCG2	4	89020602	rs756597117	intronic	c.1368-2A>C
ABCG2	4	89028412	rs1227132008	intronic	c.1195-24_1200delTTTTGCATTTTTCTTTTTGAAAAGATCATT
ABCG2	4	89013534	rs1353780966	intronic	c.1821-1G>A
ABCG2	4	89061167	rs4148151	intronic	c.-19-1G>T
ABCG2	4	89053071	rs772701413	intronic	c.264-2A>G
ABCG2	4	89053065	rs1480955544	intronic	c.264-1_267delGGTTA
ABCG2	4	89052367	rs1460346025	intronic	c.379-2A>C
ABCG2	4	89016753	rs750819307	intronic	c.1648-2A>G
ABCG2	4	89053789	rs953918059	intronic	c.204-2A>G
ABCG2	4	89015728	rs199897813	intronic	c.1820+1G>A
ABCG2	4	89080336	rs965723952	intronic	c.35+1_35+20delGTACTGATCAGCCCAATGAG
ABCG2	4	89053727	rs767710822	intronic	c.263+1G>A
ABCG2	4	89042786	rs757389397	intronic	c.689+1G>A
ABCG2	4	89053727	rs767710822	intronic	c.263+1G>T
ABCG2	4	89020475	rs753491419	intronic	c.1492+1G>A
ABCG2	4	89039260	rs773259715	intronic	c.841+1G>T
ABCG2	4	89060943	rs1282888435	intronic	c.203+2T>C
ABCG2	4	89052212	rs778595878	intronic	c.531+1G>A
ABCG2	4	89028335	rs751694038	intronic	c.1277+1G>T
ABCG2	4	89034454	rs934095463	intronic	c.1194+1G>T
ABCG2	4	89042785	rs1233865024	intronic	c.689+2T>C
ABCG2	4	89036108	rs1268719212	intronic	c.943+1G>A
ABCG2	4	89018603	rs762177463	intronic	c.1647+2T>C
ABCG2	4	89016671	rs1309363470	intronic	c.1737+1G>T
ABCG2	4	89053727	rs767710822	intronic	c.263+1G>C
ABCG2	4	89080351	rs2231134	intronic	c.35+6G>C
ABCG2	4	89022478	rs201991245	intronic	c.1278-7A>G
ABCG2	4	89039258	rs771907414	intronic	c.841+3A>G
ABCG2	4	89018762	rs754275588	intronic	c.1493-7_1493-4delGACT
ABCG2	4	89053721	rs550828327	intronic	c.263+7A>G
ABCG2	4	89039419	rs774971443	intronic	c.690-7A>C
ABCG2	4	89015819	rs576614314	intronic	c.1738-8T>C
ABCG2	4	89039256	rs201451986	intronic	c.841+5G>A
ABCG2	4	89034455	rs545664738	p.Gln398Gln	c.1194G>A
ABCG2	4	89061174	rs201830568	intronic	c.-19-8G>C
ABCG2	4	89020470	rs186806914	intronic	c.1492+6T>C
ABCG2	4	89060941	rs758456432	intronic	c.203+4T>C
ABCG2	4	89015815	rs756124936	intronic	c.1738-4A>G
ABCG2	4	89042942	rs772863640	p.Val178Val	c.534T>C
ABCG2	4	89036104	rs1480329733	intronic	c.943+5A>T
ABCG2	4	89053725	rs762391128	intronic	c.263+3G>A
ABCG2	4	89016669	rs199691084	intronic	c.1737+3A>G
ABCG2	4	89036101	rs147683935	intronic	c.943+8A>G
ABCG2	4	89079722	rs1056317920	intronic	c.95+7T>C
ABCG2	4	89036102	rs749273012	intronic	c.943+5_943+6delAT
ABCG2	4	89034449	rs770526960	intronic	c.1194+6C>G
ABCG2	4	89016769	rs760825584	intronic	c.1648-8T>A
ABCG2	4	89053725	rs762391128	intronic	c.263+3G>C
ABCG2	4	89020471	rs766129240	intronic	c.1492+5G>A
ABCG2	4	89060942	rs777654253	intronic	c.203+3A>C
ABCG2	4	89052369	rs533974194	intronic	c.379-4T>G
ABCG2	4	89015814	rs200341158	intronic	c.1738-3T>C
ABCG2	4	89052947	rs1162514827	intronic	c.378+4_378+7delAGTA
ABCG2	4	89052371	rs751179663	intronic	c.379-6C>G
ABCG2	4	89016665	rs1405725632	intronic	c.1737+7C>T
ABCG2	4	89022374	rs769535420	intronic	c.1367+8G>A
ABCG2	4	89022379	rs775283702	intronic	c.1367+3G>A
ABCG2	4	89053723	rs751958008	intronic	c.263+5G>A
ABCG2	4	89053794	rs1233837497	intronic	c.204-7T>A
ABCG2	4	89015818	rs1455095316	intronic	c.1738-7G>A
ABCG2	4	89080353	rs1305669987	intronic	c.35+4C>T
ABCG2	4	89080351	rs2231134	intronic	c.35+6G>A
ABCG2	4	89028425	rs763549571	intronic	c.1195-7T>C
ABCG2	4	89013536	rs1436734043	intronic	c.1821-3C>A
ABCG2	4	89061174	rs201830568	intronic	c.-19-8G>T
ABCG2	4	89034711	rs780882958	intronic	c.944-6G>A
ABCG2	4	89034710	rs1297179999	intronic	c.944-5C>A
ABCG2	4	89039256	rs201451986	intronic	c.841+5G>C
ABCG2	4	89036213	rs372350996	intronic	c.842-3T>C
ABCG2	4	89036214	rs1343843980	intronic	c.842-4A>G
ABCG2	4	89053077	rs760342918	intronic	c.264-8T>C
ABCG2	4	89042779	rs1420848556	intronic	c.689+8C>T
ABCG2	4	89052950	rs527531410	intronic	c.378+5G>A
ABCG2	4	89052949	rs1393462164	intronic	c.378+6T>C
ABCG2	4	89028338	rs772045758	p.Asn425Asn	c.1275C>T
ABCG2	4	89052955	rs1382386031	p.Gln126Gln	c.378A>G
ABCG2	4	89052370	rs1462801614	intronic	c.379-5T>C
ABCG2	4	89052368	rs762807841	intronic	c.379-3A>T
ABCG2	4	89022474	rs749077410	intronic	c.1278-3C>T
ABCG2	4	89022476	rs1180788441	intronic	c.1278-5G>A
ABCG2	4	89022377	rs1461561728	intronic	c.1367+5G>A
ABCG2	4	89053720	rs45536031	intronic	c.263+8T>C
ABCG2	4	89022471	rs918840244	p.Arg426Arg	c.1278A>G
ABCG2	4	89052957	rs72552713	p.Gln126Ter	c.376C>T
ABCG2	4	89039396	rs140207606	p.Arg236Ter	c.706C>T
ABCG2	4	89039366	rs200190472	p.Arg246Ter	c.736C>T
ABCG2	4	89016686	rs548254708	p.Arg575Ter	c.1723C>T
ABCG2	4	89052996	rs201121511	p.Arg113Ter	c.337C>T
ABCG2	4	89039318	rs200473953	p.Gly262Ter	c.784G>T
ABCG2	4	89016733	rs1374765124	p.Gln556Ter	c.1666C>T
ABCG2	4	89013483	rs755469457	p.Trp624Ter	c.1871G>A
ABCG2	4	89013401	rs754196551	p.Lys652ProfsTer3	c.1930_1952dupGCCTACCTGAAATTGTTATTTCT
ABCG2	4	89060982	rs201034377	p.Arg56Ter	c.166C>T
ABCG2	4	89020579	rs759166018	p.Tyr463Ter	c.1389C>A
ABCG2	4	89020576	rs753589503	p.Tyr464Ter	c.1392C>G
ABCG2	4	89016681	rs200579274	p.Tyr576Ter	c.1728T>A
ABCG2	4	89016681	rs200579274	p.Tyr576Ter	c.1728T>G
ABCG2	4	89016709	rs1481136442	p.Trp567Ter	c.1700G>A
ABCG2	4	89020574	rs773204384	p.Tyr464Ter	c.1392_1393delCA
ABCG2	4	89020486	rs1251401043	p.Tyr494Ter	c.1482C>A
ABCG2	4	89020584	rs752408502	p.Gly462Ter	c.1384G>T
ABCG2	4	89013520	rs1480156055	p.Glu612Ter	c.1834G>T
ABCG2	4	89020579	rs759166018	p.Tyr463Ter	c.1389C>G
ABCG2	4	89013512	rs1027869544	p.Trp611Ter	c.1832G>A
ABCG2	4	89053003	rs777663201	p.Ala111ArgfsTer4	c.329_330insCAGATTTATCTGATAAATTGGCAGG
ABCG2	4	89039291	rs752212395	p.Gln271Ter	c.811C>T
ABCG2	4	89034472	rs942676490	p.Gln393Ter	c.1177C>T
ABCG2	4	89061015	rs761288842	p.Arg45Ter	c.133C>T
ABCG2	4	89016718	rs1429228744	p.Trp564Ter	c.1691G>A
ABCG2	4	89034583		p.Glu356Ter	c.1066G>T
ABCG2	4	89013388	rs773361378	p.Ter656GlnextTer4	c.1966T>C
CYP2B6	19	41510282	rs12721655	p.Lys139Glu	c.415A>G
CYP2B6	19	41512841	rs3745274	p.Gln172His	c.516G>T
CYP2B6	19	41512841	rs3745274	p.Gln172Gln	c.516G>A
CYP2B6	19	41522715	rs3211371	p.Arg487Cys	c.1459C>T
CYP2B6	19	41522715	rs3211371	p.Arg487Ser	c.1459C>A
CYP2B6	19	41515263	rs2279343	p.Lys262Arg	c.785A>G
CYP2B6	19	41510311	rs145884402	p.Glu148Asp	c.444G>T
CYP2B6	19	41515911	rs139029625	p.Ala279Pro	c.835G>C
CYP2B6	19	41510208	rs139801276	p.Ile114Thr	c.341T>C
CYP2B6	19	41510063	rs186335453	p.Gly110Val	c.329G>T
CYP2B6	19	41510311	rs145884402	p.Glu148Asp	c.444G>C
CYP2B6	19	41515911	rs139029625	p.Ala279Thr	c.835G>A
CYP2B6	19	41515911	rs139029625	p.Ala279Ser	c.835G>T
CYP2B6	19	41510063	rs186335453	p.Gly110Ala	c.329G>C
CYP2B6	19	41510063	rs186335453	intronic	c.330_334+20delATATGGTGAGAGCCTCAGAGGCACT
CYP2B6	19	41510311	rs145884402	p.Glu148Glu	c.444G>A
CYP2B6	19	41518645	rs193922918	p.Phe408SerfsTer13	c.1221delC
CYP2B6	19	41518645	rs193922918	p.Ala407Thr	c.1219G>A
CYP2B6	19	41516013	rs193922917	p.Leu313Ile	c.937C>A
CYP2B6	19	41516013	rs193922917	p.Leu313Val	c.937C>G
CYP2B6	19	41515255	rs45482602	p.Ser259Arg	c.777C>A
CYP2B6	19	41515255	rs45482602	p.Ser259Ser	c.777C>T
CYP2B6	19	41515993	rs34698757	p.Thr306Ser	c.917C>G
CYP2B6	19	41515993	rs34698757	p.Thr306Ile	c.917C>T
CYP2B6	19	41518370	rs34097093	p.Arg378Ter	c.1132C>T
CYP2B6	19	41512918	rs36079186	p.Met198Thr	c.593T>C
CYP2B6	19	41512824	rs3826711	p.Pro167Ala	c.499C>G
CYP2B6	19	41522683	rs564083989	p.Gly476Asp	c.1427G>A
CYP2B6	19	41522683	rs564083989	p.Gly476Ala	c.1427G>C
CYP2B6	19	41522631	rs3211369	p.Met459Val	c.1375A>G
CYP2B6	19	41518708	rs35010098	p.Pro428Thr	c.1282C>A
CYP2B6	19	41512828	rs36056539	p.Thr168Ile	c.503C>T
CYP2B6	19	41497274	rs8192709	p.Arg22Cys	c.64C>T
CYP2B6	19	41518244	rs34826503	p.Arg336Cys	c.1006C>T
CYP2B6	19	41497286	rs33973337	p.Thr26Ser	c.76A>T
CYP2B6	19	41497295	rs33926104	p.Arg29Cys	c.85C>T
CYP2B6	19	41497296	rs34284776	p.Arg29His	c.86G>A
CYP2B6	19	41518221	rs28399499	p.Ile328Thr	c.983T>C
CYP2B6	19	41518598	rs35979566	p.Ile391Asn	c.1172T>A
CYP2B6	19	41510286	rs35773040	p.Arg140Gln	c.419G>A
CYP2B6	19	41510286	rs35773040	p.Arg140Pro	c.419G>C
CYP2B6	19	41510030	rs36060847	p.Gly99Glu	c.296G>A
CYP2B6	19	41497346	rs35303484	p.Met46Val	c.136A>G
CYP2B6	19	41497272	rs34883432	p.Gln21Leu	c.62A>T
CYP2B6	19	41497294	rs750679089	p.Arg29ProfsTer19	c.86_87delGC
CYP2B6	19	41497292	rs781463375	p.Asp28GlyfsTer30	c.82_83insGC
CYP2B6	19	41522723	rs144728904	p.Arg491AlafsTer26	c.1471delC
CYP2B6	19	41515126	rs1310137942	p.Phe217LeufsTer8	c.651delT
CYP2B6	19	41497281	rs751315964	p.Asn25Ter	c.72dupT
CYP2B6	19	41522701	rs767509932	p.Thr483AsnfsTer162	c.1447dupA
CYP2B6	19	41497214	rs762817768	p.Glu2AspfsTer55	c.6delA
CYP2B6	19	41509884	rs765912799	p.Trp11GlyfsTer31	c.31delT
CYP2B6	19	41509943	rs577681802	p.Gly71AspfsTer11	c.212delG
CYP2B6	19	41497355	rs779942397	p.Arg49LysfsTer8	c.146delG
CYP2B6	19	41518299	rs780991919	p.Ile356Ter	c.1062_1065dupTGAG
CYP2B6	19	41522696	rs761596792	p.Pro482GlnfsTer35	c.1445delC
CYP2B6	19	41512944	rs558163100	p.Leu208ThrfsTer11	c.620dupC
CYP2B6	19	41509907	rs755505792	p.Arg59GlufsTer23	c.175delC
CYP2B6	19	41518372	rs1329894263	p.Tyr380ThrfsTer13	c.1137delG
CYP2B6	19	41518210	rs769291349	p.Tyr325GlnfsTer4	c.973_974delTA
CYP2B6	19	41510026	rs183427203	p.Gly99GlufsTer15	c.296delG
CYP2B6	19	41497309	rs200768609	p.Arg35AlafsTer22	c.103delC
CYP2B6	19	41510014	rs775262892	p.Ala94ValfsTer18	c.281_287delCCTTCTC
CYP2B6	19	41512938	rs767152539	p.Ser207PhefsTer12	c.614dupC
CYP2B6	19	41518677	rs748783536	p.Ala419GlyfsTer6	c.1255dupG
CYP2B6	19	41512925	rs1256805248	p.Leu201CysfsTer24	c.602delT
CYP2B6	19	41518636	rs1363429909	p.Pro405SerfsTer2	c.1213_1226delCCAGACGCCTTCAA
CYP2B6	19	41512848	rs140582290	p.Ala176del	c.526_528delGCC
CYP2B6	19	41497261	rs764040514	p.Leu19del	c.54_56delCCT
CYP2B6	19	41516004	rs1399326269	p.Gly310_Leu315delinsVal	c.929_943delGCTTCCTGCTCATGC
CYP2B6	19	41518712	rs768220817	p.Ser430del	c.1289_1291delCCT
CYP2B6	19	41497287	rs757051573	p.His27del	c.78_80delCCA
CYP2B6	19	41522632	rs201028567	p.Ala460_Pro462dup	c.1377_1385dupGGCCAGCCC
CYP2B6	19	41512872	rs58871670	p.Val183Ile	c.547G>A
CYP2B6	19	41512843	rs148377536	p.Ser173Cys	c.518C>G
CYP2B6	19	41509934	rs138264188	p.Thr67Met	c.200C>T
CYP2B6	19	41515247	rs34646544	p.Asp257Asn	c.769G>A
CYP2B6	19	41518259	rs138030127	p.His341Asp	c.1021C>G
CYP2B6	19	41518254	rs565104467	p.Glu339Ala	c.1016A>C
CYP2B6	19	41518204	rs187378204	p.Glu322Asp	c.966G>C
CYP2B6	19	41518616	rs150072531	p.His397Arg	c.1190A>G
CYP2B6	19	41510340	rs141666881	p.Arg158Gln	c.473G>A
CYP2B6	19	41518376	rs201500445	p.Tyr380His	c.1138T>C
CYP2B6	19	41512950	rs144518874	p.Ile209Val	c.625A>G
CYP2B6	19	41518288	rs377135658	p.Glu350Asp	c.1050G>T
CYP2B6	19	41518383	rs200238771	p.Ile382Asn	c.1145T>A
CYP2B6	19	41518594	rs530338921	p.Leu390Ile	c.1168C>A
CYP2B6	19	41510007	rs772100005	p.Lys91Asn	c.273G>C
CYP2B6	19	41515266	rs1365679077	p.Asp263Gly	c.788A>G
CYP2B6	19	41522587	rs368451099	p.Ala444Val	c.1331C>T
CYP2B6	19	41518371	rs200458614	p.Arg378Gln	c.1133G>A
CYP2B6	19	41518718	rs763021653	p.Leu431Ser	c.1292T>C
CYP2B6	19	41510060	rs145946484	p.Arg109Gln	c.326G>A
CYP2B6	19	41522580	rs541481501	p.Ala442Pro	c.1324G>C
CYP2B6	19	41510059	rs142421637	p.Arg109Trp	c.325C>T
CYP2B6	19	41512962	rs371424910	p.Phe213Leu	c.637T>C
CYP2B6	19	41515898	rs74869835	intronic	c.823-1G>A
CYP2B6	19	41522549	rs767669267	intronic	c.1295-2A>C
CYP2B6	19	41522549	rs767669267	p.Lys433SerfsTer38	c.1296delG
CYP2B6	19	41509905	rs1389789429	intronic	c.172-1G>A
CYP2B6	19	41518578	rs1450196532	intronic	c.1153-1G>A
CYP2B6	19	41510070	rs373926269	intronic	c.334+2T>C
CYP2B6	19	41515301	rs1420783650	intronic	c.822+1G>T
CYP2B6	19	41510069	rs778023673	intronic	c.334+1G>C
CYP2B6	19	41518573	rs35449271	intronic	c.1153-6C>T
CYP2B6	19	41512975	rs750248652	intronic	c.645+11_645+16delCGGAGA
CYP2B6	19	41518727	rs35010579	intronic	c.1294+7T>C
CYP2B6	19	41518398	rs148605404	intronic	c.1152+8C>T
CYP2B6	19	41510194	rs764743856	intronic	c.335-8C>A
CYP2B6	19	41512811	rs150862336	p.Gly162Gly	c.486G>A
CYP2B6	19	41510203	rs577597311	p.Gly112Gly	c.336T>C
CYP2B6	19	41518398	rs148605404	intronic	c.1152+8C>A
CYP2B6	19	41522544	rs759346857	intronic	c.1295-7T>C
CYP2B6	19	41518195	rs374460130	intronic	c.965-8C>T
CYP2B6	19	41497389	rs767790930	intronic	c.171+8A>G
CYP2B6	19	41515895	rs754094512	intronic	c.823-4G>A
CYP2B6	19	41515894	rs374642809	intronic	c.823-5C>T
CYP2B6	19	41510194	rs764743856	intronic	c.335-8C>T
CYP2B6	19	41515894	rs374642809	intronic	c.823-5C>A
CYP2B6	19	41510357	rs1456870978	intronic	c.484+6T>A
CYP2B6	19	41516039	rs760684934	p.Ala321Ala	c.963A>C
CYP2B6	19	41510074	rs768573334	intronic	c.334+6A>G
CYP2B6	19	41518397	rs777016580	intronic	c.1152+7C>T
CYP2B6	19	41497385	rs759578379	intronic	c.171+4A>C
CYP2B6	19	41518576	rs757061955	intronic	c.1153-3C>G
CYP2B6	19	41515121	rs1443965936	intronic	c.646-3C>A
CYP2B6	19	41510359	rs780235731	intronic	c.484+8C>T
CYP2B6	19	41510072	rs1437566240	intronic	c.334+4A>G
CYP2B6	19	41510358	rs758644523	intronic	c.484+7C>T
CYP2B6	19	41518726	rs1410001667	intronic	c.1294+6C>G
CYP2B6	19	41512805	rs1482070693	intronic	c.485-5C>T
CYP2B6	19	41518397	rs777016580	intronic	c.1152+7C>A
CYP2B6	19	41510195	rs762547410	intronic	c.335-7A>G
CYP2B6	19	41510203	rs577597311	p.Gly112Gly	c.336T>G
CYP2B6	19	41512976	rs775562811	intronic	c.645+6G>T
CYP2B6	19	41518204	rs187378204	p.Glu322Glu	c.966G>A
CYP2B6	19	41497381	rs751682823	p.Arg57Arg	c.171G>A
CYP2B6	19	41515891	rs1328130793	intronic	c.823-8G>A
CYP2B6	19	41515893	rs1243622091	intronic	c.823-6A>G
CYP2B6	19	41515901	rs535281317	p.Glu275Glu	c.825G>A
CYP2B6	19	41509857	rs767594759	p.Met1?	c.3G>A
CYP2B6	19	41497212	rs1245049222	p.Met1?	c.2T>C
CYP2B6	19	41518265	rs201134384	p.Arg343Ter	c.1027C>T
CYP2B6	19	41515932	rs190463703	p.Gln286Ter	c.856C>T
CYP2B6	19	41510297	rs117323987	p.Glu144Ter	c.430G>T
CYP2B6	19	41512884	rs201793417	p.Arg187Ter	c.559C>T
CYP2B6	19	41509909	rs371545330	p.Arg59Ter	c.175C>T
CYP2B6	19	41510243	rs370958436	p.Arg126Ter	c.376C>T
CYP2B6	19	41515156	rs745800275	p.Tyr226Ter	c.678C>G
CYP2B6	19	41516027	rs745458475	p.Tyr317Ter	c.951C>A
CYP2B6	19	41516022	rs1296272368	p.Lys316Ter	c.946A>T
CYP2B6	19	41518279	rs1201845423	p.Tyr348Ter	c.1043_1044insATTA
CYP2C19	10	96535246	rs17884712	p.Arg144His	c.431G>A
CYP2C19	10	96535173	rs41291556	p.Trp120Arg	c.358T>C
CYP2C19	10	96541756	rs72558186	intronic	c.819+2T>C
CYP2C19	10	96612495	rs56337013	p.Arg433Trp	c.1297C>T
CYP2C19	10	96522463	rs28399504	p.Met1?	c.1A>G
CYP2C19	10	96522463	rs28399504	p.Met1?	c.1A>T
CYP2C19	10	96535124	rs12769205	intronic	c.332-23A>G
CYP2C19	10	96522450	rs367543001	intronic	c.-13G>A
CYP2C19	10	96522469	rs367543002	p.Pro3Ser	c.7C>T
CYP2C19	10	96522472	rs367543003	p.Phe4Leu	c.10T>C
CYP2C19	10	96540336	rs370803989	p.Asp188Asn	c.562G>A
CYP2C19	10	96534863	rs145328984	p.Arg73Cys	c.217C>T
CYP2C19	10	96534863	rs145328984	p.Arg73Ser	c.217C>A
CYP2C19	10	96540410	rs4986893	p.Trp212Ter	c.636G>A
CYP2C19	10	96609775	rs17886522	p.Gly417Gly	c.1251A>C
CYP2C19	10	96612542	rs118203759	p.Phe448Leu	c.1344C>A
CYP2C19	10	96612542	rs118203759	p.Phe448Leu	c.1344C>G
CYP2C19	10	96602636	rs118203757	p.Arg335Gln	c.1004G>A
CYP2C19	10	96534917	rs118203756	p.Gly91Arg	c.271G>C
CYP2C19	10	96540331	rs140278421	p.Arg186Pro	c.557G>C
CYP2C19	10	96540331	rs140278421	p.Arg186His	c.557G>A
CYP2C19	10	96540331	rs140278421	p.Arg186Leu	c.557G>T
CYP2C19	10	96541616	rs4244285	p.Pro227Pro	c.681G>A
CYP2C19	10	96612522	rs192154563	p.Arg442Cys	c.1324C>T
CYP2C19	10	96612522	rs192154563	p.Arg442Gly	c.1324C>G
CYP2C19	10	96612671	rs55640102	p.Ter491CysextTer26	c.1473A>C
CYP2C19	10	96541615	rs6413438	p.Pro227Leu	c.680C>T
CYP2C19	10	96522557	rs773529734	p.Pro35LeufsTer6	c.97_101dupCCTAC
CYP2C19	10	96540402	rs749601543	p.Trp212GlyfsTer37	c.633delC
CYP2C19	10	96535294	rs757986182	intronic	c.481+1delG
CYP2C19	10	96609715	rs765112687	p.Asn398ThrfsTer52	c.1193delA
CYP2C19	10	96522592	rs747911797	p.Asp46LeufsTer8	c.136_139delGATA
CYP2C19	10	96535285	rs752571206	p.Thr159ProfsTer19	c.475delA
CYP2C19	10	96602598	rs1438877591	p.Val323SerfsTer48	c.967delG
CYP2C19	10	96522526	rs1182559923	p.Ser23LeufsTer25	c.67_68delAG
CYP2C19	10	96602726	rs776570369	p.His368CysfsTer5	c.1097_1101dupTGCCC
CYP2C19	10	96580259	rs772230916	p.Asn277LysfsTer5	c.830dupA
CYP2C19	10	96534867	rs28399505	p.Val75TrpfsTer9	c.223delG
CYP2C19	10	96580370	rs773121944	p.Leu313ArgfsTer2	c.938delT
CYP2C19	10	96534939	rs749613282	p.His99PhefsTer5	c.294_295delCC
CYP2C19	10	96580329	rs199597498	p.Glu300AspfsTer22	c.900_901delGA
CYP2C19	10	96534942	rs765198346	p.His99GlnfsTer32	c.296_297insAG
CYP2C19	10	96534958	rs1215299915	p.Arg105SerfsTer2	c.315_316delAG
CYP2C19	10	96580363	rs1170344545	p.Leu314del	c.933_935delTCT
CYP2C19	10	96540385		p.Ile205del	c.614_616delTCA
CYP2C19	10	96535243	rs1222393260	p.Val145_Arg150del	c.432_449delTGTTCAAGAGGAAGCCCG
CYP2C19	10	96602623	rs3758581	p.Val331Ile	c.991G>A
CYP2C19	10	96534922	rs17878459	p.Glu92Asp	c.276G>C
CYP2C19	10	96535296	rs181297724	p.Ala161Pro	c.481G>C
CYP2C19	10	96540292	rs61311738	p.Ala173Val	c.518C>T
CYP2C19	10	96535264	rs58973490	p.Arg150His	c.449G>A
CYP2C19	10	96522517	rs17882687	p.Ile19Leu	c.55A>C
CYP2C19	10	96609752	rs17879685	p.Arg410Cys	c.1228C>T
CYP2C19	10	96535180	rs17885179	p.Glu122Ala	c.365A>C
CYP2C19	10	96541719	rs577255883	p.Asp262Asn	c.784G>A
CYP2C19	10	96535263	rs142974781	p.Arg150Cys	c.448C>T
CYP2C19	10	96535209	rs149590953	p.Arg132Trp	c.394C>T
CYP2C19	10	96534887	rs149072229	p.Glu81Lys	c.241G>A
CYP2C19	10	96535189	rs141774245	p.Arg125His	c.374G>A
CYP2C19	10	96602617	rs59734894	p.Arg329Cys	c.985C>T
CYP2C19	10	96535210	rs72552267	p.Arg132Gln	c.395G>A
CYP2C19	10	96612493	rs146991374	p.Lys432Ile	c.1295A>T
CYP2C19	10	96535152	rs145119820	p.Val113Ile	c.337G>A
CYP2C19	10	96602710	rs144036596	p.Asp360Asn	c.1078G>A
CYP2C19	10	96535204	rs150152656	p.Thr130Met	c.389C>T
CYP2C19	10	96602639	rs143833145	p.Ser336Ile	c.1007G>T
CYP2C19	10	96602668	rs763125128	p.Pro346Ser	c.1036C>T
CYP2C19	10	96522626	rs572853437	p.Thr55Ser	c.164C>G
CYP2C19	10	96602759	rs778043881	p.Phe376Tyr	c.1127T>A
CYP2C19	10	96580269	rs61526399	p.Gln279Pro	c.836A>C
CYP2C19	10	96580252	rs59705169	intronic	c.820-1G>C
CYP2C19	10	96612489	rs746548445	intronic	c.1292-1G>C
CYP2C19	10	96580252	rs59705169	intronic	c.820-1G>A
CYP2C19	10	96534814		intronic	c.169-1G>T
CYP2C19	10	96602593	rs1480786901	intronic	c.962-1G>C
CYP2C19	10	96540255	rs1194178489	intronic	c.482-1G>T
CYP2C19	10	96540255	rs1194178489	intronic	c.482-1G>C
CYP2C19	10	96541755	rs894663229	intronic	c.819+1G>T
CYP2C19	10	96580395	rs200506574	intronic	c.961+1G>A
CYP2C19	10	96609816	rs1241269738	intronic	c.1291+1G>T
CYP2C19	10	96535297	rs77576043	intronic	c.481+1G>T
CYP2C19	10	96602782	rs1210563088	intronic	c.1149+1G>C
CYP2C19	10	96534979	rs757977424	intronic	c.331+2T>C
CYP2C19	10	96540422	rs200936950	intronic	c.642+6G>A
CYP2C19	10	96534982	rs753021273	intronic	c.331+5G>A
CYP2C19	10	96534812	rs375151331	intronic	c.169-3T>G
CYP2C19	10	96580401	rs373898208	intronic	c.961+7T>C
CYP2C19	10	96534985	rs750969702	intronic	c.331+8T>G
CYP2C19	10	96534817	rs779668039	p.Leu57Leu	c.171C>G
CYP2C19	10	96609823	rs777490712	intronic	c.1291+8A>T
CYP2C19	10	96534817	rs779668039	p.Leu57Leu	c.171C>T
CYP2C19	10	96522637	rs769135473	intronic	c.168+7A>G
CYP2C19	10	96540250	rs776181631	intronic	c.482-6A>G
CYP2C19	10	96540416	rs751623882	p.Gln214Gln	c.642G>A
CYP2C19	10	96602595	rs1178582722	p.Ala321Ala	c.963T>G
CYP2C19	10	96540251	rs17882507	intronic	c.482-5T>G
CYP2C19	10	96541574	rs767505657	intronic	c.643-4T>A
CYP2C19	10	96609666	rs780346574	intronic	c.1150-8G>T
CYP2C19	10	96580397	rs137862636	intronic	c.961+3A>G
CYP2C19	10	96602784	rs533047949	intronic	c.1149+3A>C
CYP2C19	10	96541754	rs1262244551	p.Lys273Lys	c.819G>A
CYP2C19	10	96522637	rs769135473	intronic	c.168+7A>T
CYP2C19	10	96612487	rs779364750	intronic	c.1292-3C>T
CYP2C19	10	96602789	rs768493491	intronic	c.1149+8T>A
CYP2C19	10	96602781	rs772082164	p.Lys383Lys	c.1149G>A
CYP2C19	10	96580399	rs369778631	intronic	c.961+5G>A
CYP2C19	10	96535141	rs776842113	intronic	c.332-6T>C
CYP2C19	10	96535140	rs769068984	intronic	c.332-7C>T
CYP2C19	10	96541572	rs1399729979	intronic	c.643-6T>C
CYP2C19	10	96541758	rs199556059	intronic	c.819+4A>T
CYP2C19	10	96609822	rs1215318388	intronic	c.1291+7T>A
CYP2C19	10	96609821	rs756104782	intronic	c.1291+6A>G
CYP2C19	10	96609820	rs1288826456	intronic	c.1291+5T>C
CYP2C19	10	96540423	rs191690054	intronic	c.642+7C>T
CYP2C19	10	96609814	rs781375063	p.Ala430Ala	c.1290A>T
CYP2C19	10	96522638	rs777031765	intronic	c.168+8T>C
CYP2C19	10	96602589	rs761128535	intronic	c.962-5G>C
CYP2C19	10	96602587	rs775646392	intronic	c.962-7T>C
CYP2C19	10	96522630	rs745382759	p.Asn56Asn	c.168T>C
CYP2C19	10	96602595	rs1178582722	p.Ala321Ala	c.963T>C
CYP2C19	10	96612491	rs773833388	p.Gly431Gly	c.1293A>C
CYP2C19	10	96535299	rs1259801750	intronic	c.481+3G>A
CYP2C19	10	96535301	rs766957203	intronic	c.481+5G>A
CYP2C19	10	96535301	rs766957203	intronic	c.481+5G>C
CYP2C19	10	96535298	rs763491011	intronic	c.481+5delG
CYP2C19	10	96580245	rs773967881	intronic	c.820-8A>T
CYP2C19	10	96580248	rs545765847	intronic	c.820-5C>T
CYP2C19	10	96535141	rs776842113	intronic	c.332-6T>G
CYP2C19	10	96602769	rs771120274	p.Tyr379Ter	c.1137C>A
CYP2C19	10	96534881	rs1410213756	p.Gly79Ter	c.235G>T
CYP2C19	10	96535278	rs374036992	p.Glu155Ter	c.463G>T
CYP2C19	10	96580265	rs751802597	p.Gln278Ter	c.832C>T
CYP2C19	10	96535282	rs1157042274	p.Leu156Ter	c.467T>A
CYP2C19	10	96541689	rs748628122	p.Gln252Ter	c.754C>T
CYP2C19	10	96541749	rs749564114	p.Glu272Ter	c.814G>T
CYP2C19	10	96540409	rs751429804	p.Trp212Ter	c.635G>A
CYP2C19	10	96522492	rs779922341	p.Cys10Ter	c.30T>A
CYP2C19	10	96612510	rs1187954902	p.Glu438Ter	c.1312G>T
CYP2C19	10	96522592	rs747911797	p.Gln44Ter	c.130C>T
CYP2C19	10	96612538	rs1361457533	p.Leu447Ter	c.1340T>A
CYP2C19	10	96540324	rs961342096	p.Gln184Ter	c.550C>T
CYP2C19	10	96580286	rs141533135	p.Glu285Ter	c.853G>T
CYP2C19	10	96534971	rs1429211377	p.Gly109Ter	c.325G>T
CYP2C8	10	96818171	rs769460274	p.Lys247Arg	c.740A>G
CYP2C8	10	96824642	rs543793530	p.Arg186Gln	c.557G>A
CYP2C8	10	96824642	rs543793530	p.Arg186Leu	c.557G>T
CYP2C8	10	96824643	rs72558195	p.Arg186Gly	c.556C>G
CYP2C8	10	96824643	rs72558195	p.Arg186Ter	c.556C>T
CYP2C8	10	96824688	rs142886225	p.Gly171Ser	c.511G>A
CYP2C8	10	96826970	rs72558196	p.Thr159ProfsTer19	c.475delA
CYP2C8	10	96818119	rs1058930	p.Ile264Met	c.792C>G
CYP2C8	10	96818119	rs1058930	p.Ile264Ile	c.792C>T
CYP2C8	10	96798749	rs10509681	p.Lys399Arg	c.1196A>G
CYP2C8	10	96827030	rs11572080	p.Arg139Lys	c.416G>A
CYP2C8	10	96818106	rs11572103	p.Ile269Phe	c.805A>T
CYP2C8	10	96802715	rs45438799	p.Leu361Phe	c.1081C>T
CYP2C8	10	96802715	rs45438799	p.Leu361Ile	c.1081C>A
CYP2C8	10	96818181	rs11572102	p.Ile244Val	c.730A>G
CYP2C8	10	96818181	rs11572102	p.Ile244Phe	c.730A>T
CYP2C8	10	96802804	rs146806199	p.Ile331Thr	c.992T>C
CYP2C8	10	96802804	rs146806199	p.Ile331Ser	c.992T>G
CYP2C8	10	96824658	rs41286886	p.Val181Ile	c.541G>A
CYP2C8	10	96818199	rs188934928	p.Ala238Pro	c.712G>C
CYP2C8	10	96818242	not assigned	p.Ile223Met	c.669T>G
CYP2C8	10	96796977	rs3832694	p.Val461dup	c.1382_1384dup
CYP2C8	10	96805708	rs78637571	p.Glu274Ter	c.820G>T
CYP2C8	10	96802647	not assigned	p.Lys383Asp	c.1149C>A
CYP2C8	10	96827178	rs2275622	p.Lys4Glu	c.10A>G
CYP2C8	10	96827150	rs11572076	p.Cys13Tyr	c.38G>A
CYP2C8	10	96827126	rs143096432	p.Leu21Pro	c.62T>C
CYP2C8	10	96798795	rs143386810	p.Gly384Ser	c.1150G>A
CYP2C8	10	96827016	rs540288649	p.Arg144Gly	c.430C>G
CYP2C8	10	96802703	rs77147096	p.Gly365Ser	c.1093G>A
CYP2C8	10	96800713	rs573187322	p.Phe299Tyr	c.896T>A
CYP2C8	10	96827052	rs145992929	p.Arg132Trp	c.394C>T
CYP2C8	10	96827354	rs201449274	p.Ile88Thr	c.263T>C
CYP2C8	10	96826974	rs201561213	p.Lys158Glu	c.472A>G
CYP2C8	10	96802768	rs148442781	p.Ser343Ile	c.1028G>T
CYP2C8	10	96802700	rs147133669	p.Val366Met	c.1096G>A
CYP2C8	10	96818158	rs781159467	p.His251Gln	c.753C>A
CYP2C8	10	96798695	rs141209951	p.Gly417Val	c.1250G>T
CYP2C8	10	96798669	rs148348784	p.Met426Val	c.1276A>G
CYP2C8	10	96829097	rs202131138	p.Arg21Ser	c.63A>T
CYP2C8	10	96827115	rs1053507227	intronic	c.332-1G>A
CYP2C8	10	96827449	rs769016577	intronic	c.169-1G>T
CYP2C8	10	96797068	rs769405632	intronic	c.1292-2A>C
CYP2C8	10	96798796	rs1162778112	intronic	c.1150-1G>C
CYP2C8	10	96800718	rs1269496402	intronic	c.892-1G>C
CYP2C8	10	96818270	rs1192116627	intronic	c.643-2A>C
CYP2C8	10	96805709	rs1238596821	intronic	c.820-1G>C
CYP2C8	10	96827284	rs199931273	intronic	c.331+1delG
CYP2C8	10	96827284	rs199931273	intronic	c.331+2T>C
CYP2C8	10	96828953	rs368939862	intronic	c.25+1G>A
CYP2C8	10	96826964	rs772754152	intronic	c.481+1G>T
CYP2C8	10	96805566	rs781632266	intronic	c.961+1G>A
CYP2C8	10	96826966	rs11572081	p.Lys160Lys	c.480G>A
CYP2C8	10	96827118	rs11572079	intronic	c.332-4T>C
CYP2C8	10	96797074	rs186730985	intronic	c.1292-8A>G
CYP2C8	10	96824553	rs368695340	intronic	c.642+4A>C
CYP2C8	10	96802642	rs542760737	intronic	c.1149+5G>A
CYP2C8	10	96805716	rs779062442	intronic	c.820-8A>G
CYP2C8	10	96824725	rs1352057373	intronic	c.482-8A>C
CYP2C8	10	96827452	rs1156763423	intronic	c.169-4C>T
CYP2C8	10	96826960	rs368299637	intronic	c.481+5G>T
CYP2C8	10	96802641	rs760009863	intronic	c.1149+6C>G
CYP2C8	10	96798649	rs755108098	intronic	c.1291+5T>C
CYP2C8	10	96798799	rs375830877	intronic	c.1150-4C>T
CYP2C8	10	96798793	rs749768223	p.Gly384Gly	c.1152C>T
CYP2C8	10	96828986	rs372227542	intronic	c.168+6T>C
CYP2C8	10	96828989	rs764983183	intronic	c.168+3A>G
CYP2C8	10	96826961	rs773255712	intronic	c.481+4G>T
CYP2C8	10	96827281	rs757297073	intronic	c.331+5G>T
CYP2C8	10	96802841	rs1243336731	intronic	c.962-12_962-8delGTGTC
CYP2C8	10	96818266	rs770367683	p.Val215Val	c.645C>T
CYP2C8	10	96828950	rs1353876818	intronic	c.25+4A>G
CYP2C8	10	96827446	rs747486167	p.Phe57Phe	c.171C>T
CYP2C8	10	96802643	rs371605006	intronic	c.1149+4A>T
CYP2C8	10	96824551	rs1026631336	intronic	c.642+6G>A
CYP2C8	10	96824552	rs748888030	intronic	c.642+5G>A
CYP2C8	10	96802644	rs761921124	intronic	c.1149+3A>G
CYP2C8	10	96828985	rs1406468663	intronic	c.168+7C>G
CYP2C8	10	96828987	rs1165949768	intronic	c.168+5G>A
CYP2C8	10	96828992	rs144284469	p.Asn56Asn	c.168T>C
CYP2C8	10	96805715	rs757215714	intronic	c.820-7C>T
CYP2C8	10	96827279	rs1362315712	intronic	c.331+7G>A
CYP2C8	10	96827280	rs754076612	intronic	c.331+6T>A
CYP2C8	10	96826959	rs1248245045	intronic	c.481+5dupG
CYP2C8	10	96826958	rs1221955267	intronic	c.481+7G>C
CYP2C8	10	96798802	rs759790473	intronic	c.1150-7C>T
CYP2C8	10	96826960	rs368299637	intronic	c.481+5G>A
CYP2C8	10	96829159	rs142470035	p.Met1?	c.1A>G
CYP2C8	10	96829159	rs142470035	p.Met1?	c.1A>C
CYP2C8	10	96827187	rs1280831429	p.Met1?	c.1A>T
CYP2C8	10	96827187	rs1280831429	p.Met1?	c.1A>C
CYP2C8	10	96829159	rs142470035	p.Met1?	c.1A>T
CYP2C8	10	96824564	rs146962089	p.Trp212Ter	c.635G>A
CYP2C8	10	96827120	rs770873636	p.Leu23Ter	c.68T>A
CYP2C8	10	96824674	rs141350682	p.Cys175Ter	c.525C>A
CYP2C8	10	96818190	rs536085663	p.Arg241Ter	c.721C>T
CYP2C8	10	96829008	rs776194558	p.Cys51Ter	c.151_152insA
CYP2C8	10	96829099	rs757789288	p.Arg21Ter	c.61A>T
CYP2C8	10	96829007	rs756493843	p.Cys51Ter	c.153C>A
CYP2C8	10	96798747	rs181982392	p.Glu400Ter	c.1198G>T
CYP2C8	10	96824683	rs553407481	p.Cys172Ter	c.516T>A
CYP2C8	10	96828969	rs763891380	p.Gln4Ter	c.10C>T
CYP2C8	10	96826986	rs767098538	p.Glu154Ter	c.460G>T
CYP2C8	10	96802755	rs1033533057	p.Tyr347Ter	c.1041C>G
CYP2C8	10	96805604	rs771704932	p.Tyr308Ter	c.924T>G
CYP2C8	10	96818268	rs148974310	p.Val215Ter	c.643-1_643insTAAAG
CYP2C8	10	96818229	rs747957327	p.Gly228Ter	c.682G>T
CYP2C8	10	96828954	rs774260908	p.Gly9Ter	c.25G>T
CYP2C8	10	96827444	rs780544107	p.Ser58Ter	c.173C>G
CYP2C8	10	96829101		p.Trp20Ter	c.59G>A
CYP2C8	10	96827434	rs1440057227	p.Tyr61Ter	c.183T>A
CYP2C8	10	96796908	rs764390443	p.Gln484Ter	c.1450C>T
CYP2C8	10	96796909	rs1364415240	p.Tyr483Ter	c.1449C>A
CYP2C8	10	96827382	rs776856979	p.Gly79Ter	c.235G>T
CYP2C8	10	96827307	rs1233586913	p.Gln104Ter	c.310C>T
CYP2C8	10	96827086	rs1354192764	p.Trp120Ter	c.360G>A
CYP2C9	10	96708974	rs2256871	p.His251Arg	c.752A>G
CYP2C9	10	96702066	rs7900194	p.Arg150His	c.449G>A
CYP2C9	10	96702066	rs7900194	p.Arg150Leu	c.449G>T
CYP2C9	10	96702066	rs7900194	p.Arg150Pro	c.449G>C
CYP2C9	10	96698494	rs67807361	p.Leu19Ile	c.55C>A
CYP2C9	10	96707626	not assigned	p.Asp191Gly	c.572A>G
CYP2C9	10	96741128	rs542577750	intronic	c.1149+1G>A
CYP2C9	10	96748609	rs776908257	p.Arg433Trp	c.1297C>T
CYP2C9	10	96748609	rs776908257	p.Arg433Arg	c.1297C>A
CYP2C9	10	96741062	rs578144976	p.Leu362Val	c.1084C>G
CYP2C9	10	96740956	rs147665916	p.Glu326Glu	c.978G>A
CYP2C9	10	96702048	rs141489852	p.Arg144His	c.431G>A
CYP2C9	10	96701990	rs375805362	p.Arg125Cys	c.373C>T
CYP2C9	10	96748682	rs202201137	p.Asn457Ser	c.1370A>G
CYP2C9	10	96748712	rs767284820	p.Leu467Pro	c.1400T>C
CYP2C9	10	96709038	rs9332131	p.Lys273ArgfsTer34	c.818delA
CYP2C9	10	96748612	not assigned	p.Ile434Phe	c.1300A>T
CYP2C9	10	96740987	rs1274535931	p.Pro337Thr	c.1009C>A
CYP2C9	10	96707665	rs1469057029	p.Asn204Ser	c.611A>G
CYP2C9	10	96707664	not assigned	p.Asn204His	c.610A>C
CYP2C9	10	96741059	rs1250577724	p.Leu361Ile	c.1081C>A
CYP2C9	10	96741059	rs1250577724	p.Leu361Phe	c.1081C>T
CYP2C9	10	96731937	rs988617574	p.Thr299Arg	c.896C>G
CYP2C9	10	96731937	rs988617574	p.Thr299Lys	c.896C>A
CYP2C9	10	96731891	not assigned	p.Ile284Val	c.850A>G
CYP2C9	10	96708902	rs772782449	p.Pro227Leu	c.680C>T
CYP2C9	10	96708902	rs772782449	p.Pro227Gln	c.680C>A
CYP2C9	10	96708903	rs772651628	p.Pro227Pro	c.681G>A
CYP2C9	10	96708901	not assigned	p.Pro227Ser	c.679C>T
CYP2C9	10	96741058	rs28371686	p.Asp360Glu	c.1080C>G
CYP2C9	10	96741058	rs28371686	p.Asp360Glu	c.1080C>A
CYP2C9	10	96707674	rs1326630788	p.Ile207Thr	c.620T>C
CYP2C9	10	96707542	rs774550549	p.Pro163Leu	c.488C>T
CYP2C9	10	96702011	rs199523631	p.Arg132Trp	c.394C>T
CYP2C9	10	96701987	rs767576260	p.Arg124Trp	c.370C>T
CYP2C9	10	96701973	rs774607211	p.Lys119Arg	c.356A>G
CYP2C9	10	96701973	rs774607211	p.Lys119Thr	c.356A>C
CYP2C9	10	96741054	rs56165452	p.Ile359Thr	c.1076T>C
CYP2C9	10	96701733	rs761033063	p.Gly96Val	c.287G>T
CYP2C9	10	96740982	rs367826293	p.Arg335Gln	c.1004G>A
CYP2C9	10	96702012	rs200183364	p.Arg132Gln	c.395G>A
CYP2C9	10	96748780	rs868182778	p.Val490Phe	c.1468G>T
CYP2C9	10	96740958	rs57505750	p.Ile327Thr	c.980T>C
CYP2C9	10	96741053	rs1057910	p.Ile359Leu	c.1075A>C
CYP2C9	10	96741053	rs1057910	p.Ile359Val	c.1075A>G
CYP2C9	10	96731876	rs182132442	p.Pro279Thr	c.835C>A
CYP2C9	10	96701672	rs1216169538	p.Val76Leu	c.226G>T
CYP2C9	10	96698528	rs142240658	p.Pro30Leu	c.89C>T
CYP2C9	10	96702047	rs1799853	p.Arg144Cys	c.430C>T
CYP2C9	10	96748674	rs769942899	p.Gln454His	c.1362G>C
CYP2C9	10	96731880	not assigned	p.Ser280Cys	c.839C>G
CYP2C9	10	96748637	not assigned	p.Gly442Val	c.1325G>T
CYP2C9	10	96731936	rs72558192	p.Thr299Ala	c.895A>G
CYP2C9	10	96707539	rs72558190	p.Ser162Leu	c.485C>T
CYP2C9	10	96701991	rs72558189	p.Arg125His	c.374G>A
CYP2C9	10	96701991	rs72558189	p.Arg125Leu	c.374G>T
CYP2C9	10	96701991	rs72558189	p.Arg125Pro	c.374G>C
CYP2C9	10	96701715	rs72558187	p.Leu90Pro	c.269T>C
CYP2C9	10	96748777	rs9332239	p.Pro489Ser	c.1465C>T
CYP2C9	10	96740981	rs28371685	p.Arg335Trp	c.1003C>T
CYP2C9	10	96708885	rs1176883016	p.Ile222SerfsTer19	c.664delA
CYP2C9	10	96748645	rs1221842495	p.Leu447IlefsTer10	c.1335_1336delGT
CYP2C9	10	96707624	rs777921891	p.Asp191IlefsTer7	c.571delG
CYP2C9	10	96740941	rs767003960	p.Val323SerfsTer6	c.966dupA
CYP2C9	10	96740989	rs760277731	p.Cys338MetfsTer23	c.1011_1012insA
CYP2C9	10	96708958	rs915366633	p.Val248Ter	c.741delA
CYP2C9	10	96740948	rs1454934321	p.Gln324ArgfsTer7	c.971delA
CYP2C9	10	96748734	rs752055497	p.Gly475AspfsTer45	c.1424delG
CYP2C9	10	96709034	rs766429303	p.Glu272ArgfsTer35	c.814delG
CYP2C9	10	96707649	rs562386989	p.Lys200SerfsTer2	c.599delA
CYP2C9	10	96707636	rs1281128973	p.Leu197GlyfsTer5	c.588_592delCTTAA
CYP2C9	10	96698602	rs1394457973	p.Asn56IlefsTer16	c.165delC
CYP2C9	10	96707615	rs1484019612	p.Asp188IlefsTer10	c.562delG
CYP2C9	10	96698519	rs762769573	p.Lys28AsnfsTer11	c.84delA
CYP2C9	10	96698511	rs1320227926	p.Arg26GlufsTer13	c.75delG
CYP2C9	10	96731946	rs768422675	p.Ser303AlafsTer4	c.907delA
CYP2C9	10	96701619	rs767093588	p.Val60LysfsTer23	c.174_177dupAAAG
CYP2C9	10	96701640	rs1414631924	p.Thr66SerfsTer15	c.196_197delAC
CYP2C9	10	96701718	rs750075832	p.Glu93AlafsTer32	c.278_293delAGTTTTCTGGAAGAGG
CYP2C9	10	96707628	rs747018687	p.Gln193_Glu199del	c.576_596delGCAATTTCTTAACTTAATGGA
CYP2C9	10	96731974	rs779081764	p.Leu314del	c.936_938delCCT
CYP2C9	10	96748636	rs368545396	p.Gly442Ser	c.1324G>A
CYP2C9	10	96707574	rs199539783	p.Pro174Ala	c.520C>G
CYP2C9	10	96698453	rs138957855	p.Val5Ala	c.14T>C
CYP2C9	10	96741073	rs139532088	p.Ser365Arg	c.1095C>A
CYP2C9	10	96745904	rs776769484	p.Ser422Gly	c.1264A>G
CYP2C9	10	96741012	rs201055266	p.Met345Thr	c.1034T>C
CYP2C9	10	96701736	rs374201833	p.Arg97Thr	c.290G>C
CYP2C9	10	96702006	rs200965026	p.Thr130Met	c.389C>T
CYP2C9	10	96701960	rs771237265	p.Ser115Arg	c.343A>C
CYP2C9	10	96741066	rs150663116	p.Pro363Leu	c.1088C>T
CYP2C9	10	96708864	rs759708513	intronic	c.643-1G>C
CYP2C9	10	96748603	rs768961868	intronic	c.1292-1G>A
CYP2C9	10	96701947	rs111553080	intronic	c.332-2A>G
CYP2C9	10	96701614	rs1272084617	intronic	c.169-1G>C
CYP2C9	10	96745932	rs750312180	intronic	c.1291+1G>T
CYP2C9	10	96698608	rs763692476	intronic	c.168+2dupT
CYP2C9	10	96702099	rs1253395922	intronic	c.481+1G>T
CYP2C9	10	96731854	rs199940588	intronic	c.820-7T>C
CYP2C9	10	96708859	rs1437869033	intronic	c.643-6T>C
CYP2C9	10	96740933	rs1014258369	intronic	c.962-7T>G
CYP2C9	10	96707696	rs370100007	p.Gln214Gln	c.642G>A
CYP2C9	10	96701783	rs1188683939	intronic	c.331+6T>C
CYP2C9	10	96709041	rs1490080890	p.Lys273Lys	c.819G>A
CYP2C9	10	96709044	rs1199744187	intronic	c.819+3A>G
CYP2C9	10	96745938	rs1308117422	intronic	c.1291+7T>C
CYP2C9	10	96701943	rs199560417	intronic	c.332-6T>A
CYP2C9	10	96745784	rs768049150	intronic	c.1150-6T>A
CYP2C9	10	96708860	rs779666293	intronic	c.643-5C>T
CYP2C9	10	96745935	rs140288914	intronic	c.1291+4A>G
CYP2C9	10	96707527	rs753274809	intronic	c.482-5_482-2delTTTA
CYP2C9	10	96731853	rs778521455	intronic	c.820-8A>G
CYP2C9	10	96708860	rs779666293	intronic	c.643-5C>A
CYP2C9	10	96707702	rs371267733	intronic	c.642+6G>A
CYP2C9	10	96745785	rs775851127	intronic	c.1150-5T>C
CYP2C9	10	96701946	rs559536673	intronic	c.332-3T>C
CYP2C9	10	96745782	rs376370397	intronic	c.1150-8G>A
CYP2C9	10	96701607	rs776477272	intronic	c.169-8T>A
CYP2C9	10	96709047	rs751492181	intronic	c.819+6A>G
CYP2C9	10	96709047	rs751492181	intronic	c.819+6A>C
CYP2C9	10	96709045	rs1205284692	intronic	c.819+4A>G
CYP2C9	10	96745936	rs1226562348	intronic	c.1291+5T>C
CYP2C9	10	96702102	rs1455377309	intronic	c.481+4G>T
CYP2C9	10	96702103	rs1191697976	intronic	c.481+5G>A
CYP2C9	10	96701941	rs779778121	intronic	c.332-8C>T
CYP2C9	10	96741135	rs1304755974	intronic	c.1149+8T>C
CYP2C9	10	96745792	rs1367734521	p.Gly384Gly	c.1152C>T
CYP2C9	10	96731853	rs778521455	intronic	c.820-8A>T
CYP2C9	10	96731858	rs1227634501	intronic	c.820-3T>C
CYP2C9	10	96731857	rs1326623780	intronic	c.820-4C>T
CYP2C9	10	96731855	rs1289885159	intronic	c.820-4delC
CYP2C9	10	96748600	rs747358021	intronic	c.1292-4T>G
CYP2C9	10	96701781	rs1206932324	intronic	c.331+4G>A
CYP2C9	10	96701782	rs1282471396	intronic	c.331+5G>T
CYP2C9	10	96698440	rs114071557	p.Met1?	c.1A>G
CYP2C9	10	96698442	rs150891702	p.Met1?	c.3G>T
CYP2C9	10	96740948	rs1454934321	p.Gln324Ter	c.970C>T
CYP2C9	10	96740996	rs767109263	p.Gln340Ter	c.1018C>T
CYP2C9	10	96701756	rs755877934	p.Glu104Ter	c.310G>T
CYP2C9	10	96698503	rs777845146	p.Gln22Ter	c.64C>T
CYP2C9	10	96708970	rs945414728	p.Glu250Ter	c.748G>T
CYP2C9	10	96708976	rs1253597502	p.Gln252Ter	c.754C>T
CYP2C9	10	96708979	rs777095173	p.Glu253Ter	c.757G>T
CYP2C9	10	96741038	rs749060448	p.Glu354Ter	c.1060G>T
CYP2C9	10	96701686	rs200794294	p.Tyr80Ter	c.240T>A
CYP2C9	10	96748672	rs781427480	p.Gln454Ter	c.1360C>T
CYP2D6	22	42525829	rs267608276	p.Arg88Pro	c.263G>C
CYP2D6	22	42525829	rs267608276	p.Arg88His	c.263G>A
CYP2D6	22	42522898	rs763964554	p.Gln424Ter	c.1270C>T
CYP2D6	22	42524175	rs5030656	p.Lys281del	c.841_843delAAG
CYP2D6	22	42524175	rs5030656	intronic	c.843+1G>A
CYP2D6	22	42524219	rs148769737	p.Pro267His	c.800C>A
CYP2D6	22	42524219	rs148769737	p.Pro267Leu	c.800C>T
CYP2D6	22	42524219	rs148769737	p.Pro267Arg	c.800C>G
CYP2D6	22	42526776	rs148382141	p.Leu6Leu	c.18G>A
CYP2D6	22	42523475	rs75386357	p.Glu383Lys	c.1147G>A
CYP2D6	22	42523475	rs75386357	p.Glu383Gln	c.1147G>C
CYP2D6	22	42523858	rs5030867	p.His324Pro	c.971A>C
CYP2D6	22	42525733	rs267608289	intronic	c.352+7A>G
CYP2D6	22	42522749	rs730882251	p.Arg441Cys	c.1321C>T
CYP2D6	22	42525085	rs5030655	p.Trp152GlyfsTer2	c.454delT
CYP2D6	22	42523854	rs79292917	p.Pro325Pro	c.975G>A
CYP2D6	22	42523592	rs72549347	p.Arg344Ter	c.1030C>T
CYP2D6	22	42524237	rs267608297	p.Thr261Ile	c.782C>T
CYP2D6	22	42525073	rs267608302	p.Glu156Ala	c.467A>C
CYP2D6	22	42525182	rs1135822	p.Phe120Ile	c.358T>A
CYP2D6	22	42525182	rs1135822	p.Phe120Leu	c.358T>C
CYP2D6	22	42526721	rs267608313	p.Arg25Trp	c.73C>T
CYP2D6	22	42523843	rs72549349	intronic	c.985+1G>A
CYP2D6	22	42523805	rs28371725	intronic	c.985+39G>A
CYP2D6	22	42524947	rs3892097	intronic	c.506-1G>A
CYP2D6	22	42523943	rs16947	p.Cys296Arg	c.886T>C
CYP2D6	22	42523943	rs16947	p.Cys296Ser	c.886T>A
CYP2D6	22	42522751	rs267608319	p.Arg440His	c.1319G>A
CYP2D6	22	42524243	rs35742686	p.Arg259GlyfsTer2	c.775delA
CYP2D6	22	42525134	rs61736512	p.Val136Ile	c.406G>A
CYP2D6	22	42523610	rs59421388	p.Val338Met	c.1012G>A
CYP2D6	22	42522613	rs1135840	p.Thr486Ser	c.1457C>G
CYP2D6	22	42525132	rs1058164	p.Val136Val	c.408C>G
CYP2D6	22	42525772	rs28371706	p.Thr107Ile	c.320C>T
CYP2D6	22	42525772	rs28371706	p.Thr107Asn	c.320C>A
CYP2D6	22	42526656	rs774671100	p.Leu47AlafsTer207	c.137dupT
CYP2D6	22	42526670	rs5030862	p.Gly42Arg	c.124G>A
CYP2D6	22	42525912	rs201377835	intronic	c.181-1G>C
CYP2D6	22	42523847	rs730882170	p.Met321IlefsTer12	c.963_981delGATCCTACATCCGGATGTG
CYP2D6	22	42526694	rs1065852	p.Pro34Ser	c.100C>T
CYP2D6	22	42524213	rs72549352	p.Arg269ProfsTer5	c.805dupC
CYP2D6	22	42523532	rs747955910	p.Gln364CysfsTer12	c.1088_1089dupTG
CYP2D6	22	42524250	rs558523758	p.Thr256SerfsTer4	c.765_768delAACT
CYP2D6	22	42522982	rs757396767	p.Leu395HisfsTer11	c.1184_1185delTC
CYP2D6	22	42522916	rs28371733	p.Glu418AsnfsTer11	c.1251delC
CYP2D6	22	42525861	rs781012191	p.Pro77ArgfsTer16	c.230delC
CYP2D6	22	42524202	rs767565288	p.Glu273Ter	c.816dupT
CYP2D6	22	42523547	rs79489631	p.Val359ProfsTer4	c.1067_1074dupCCACTGCC
CYP2D6	22	42524217	rs778851115	p.Pro268SerfsTer6	c.797_801dupAGCCC
CYP2D6	22	42523609	rs771811053	p.Val338GlyfsTer12	c.1012dupG
CYP2D6	22	42526743		p.Phe16CysfsTer6	c.47_50delTCCT
CYP2D6	22	42526701		p.Ala31AspfsTer62	c.92delC
CYP2D6	22	42526681	rs1287287392	p.Leu38AlafsTer215	c.111_112delCC
CYP2D6	22	42524929		p.Asn175PhefsTer86	c.522_523insTTTCGCCCCTTTCGCCCCTTTC
CYP2D6	22	42526784	rs1240247139	p.Glu4ArgfsTer250	c.9dupA
CYP2D6	22	42525892	rs745568317	p.Asp67ThrfsTer26	c.199delG
CYP2D6	22	42524884	rs773148044	p.Leu189ProfsTer19	c.564_567delCCTC
CYP2D6	22	42526768		p.Leu9TrpfsTer3	c.25delC
CYP2D6	22	42522920	rs764444172	p.His416ProfsTer13	c.1247delA
CYP2D6	22	42525790	rs756966320	p.Arg101ProfsTer160	c.280_301dupCACGGCGAGGACACCGCCGACC
CYP2D6	22	42525783	rs1376617960	p.Val104AlafsTer151	c.305_308dupCGCC
CYP2D6	22	42525088	rs770277909	p.Gln151SerfsTer3	c.451delC
CYP2D6	22	42522657	rs1243903452	p.Gln472ThrfsTer20	c.1412dupG
CYP2D6	22	42524808	rs1256122634	p.Glu215ArgfsTer10	c.643delG
CYP2D6	22	42522647	rs973966643	p.Ser476AlafsTer10	c.1422delG
CYP2D6	22	42524334	rs993385303	p.Pro228ArgfsTer25	c.683_684delCC
CYP2D6	22	42523451	rs745931147	p.Lys391Ter	c.1170dupT
CYP2D6	22	42526780	rs773790593	p.Glu4del	c.11_13delAAG
CYP2D6	22	42524809	rs567606867	p.Lys214del	c.640_642delAAG
CYP2D6	22	42524178	rs762158210	p.Glu280del	c.838_840delGAG
CYP2D6	22	42524903	rs774778807	p.Ser183del	c.546_548delGAG
CYP2D6	22	42523619		p.Glu334del	c.1000_1002delGAG
CYP2D6	22	42523609	rs771811053	p.Asp337del	c.1010_1012delACG
CYP2D6	22	42522698	rs375715419	p.Phe457del	c.1369_1371delTTC
CYP2D6	22	42522681	rs749471493	p.Gln462del	c.1386_1388delGCA
CYP2D6	22	42525091	rs1214476453	p.Gly145_Leu149del	c.434_448delGCAAGAAGTCGCTGG
CYP2D6	22	42524254	rs767062512	p.Leu255del	c.762_764delGCT
CYP2D6	22	42524326	rs867154253	p.Leu231del	c.690_692delCCT
CYP2D6	22	42523898	rs1252148623	p.Thr310del	c.928_930delACC
CYP2D6	22	42524929		p.Pro174_Asn175insPheArgProPheArgPro	c.522_523insTTTCGCCCCTTTCGCCCC
CYP2D6	22	42524929		p.Phe172_Pro174dup	c.514_522dupTTTCGCCCC
CYP2D6	22	42524929		p.Pro174_Asn175insPheArgProPheArgProPheArgPro	c.522_523insTTTCGCCCCTTTCGCCCCTTTCGCCCC
CYP2D6	22	42525180	rs61736507	p.Val119_Phe120insLeu	c.357_359dupGTT
CYP2D6	22	42522658	rs765776661	p.Val468_Thr470dup	c.1403_1411dupTGCCCACTG
CYP2D6	22	42524254	rs767062512	p.Leu255dup	c.762_764dupGCT
CYP2D6	22	42526549	rs1081000	intronic	c.180+65G>A
CYP2D6	22	42526580	rs1080995	intronic	c.180+34C>G
CYP2D6	22	42526567		intronic	c.180+47C>T
CYP2D6	22	42526571		intronic	c.180+43G>C
CYP2D6	22	42526573	rs1080996	intronic	c.180+41A>C
CYP2D6	22	42526561	rs28695233	intronic	c.180+53C>A
CYP2D6	22	42526562		intronic	c.180+52C>G
CYP2D6	22	42525952	rs28371702	intronic	c.181-41G>T
CYP2D6	22	42523003	rs4987144	intronic	c.1174-9T>C
CYP2D6	22	42524130	rs28371722	intronic	c.843+46G>A
CYP2D6	22	42524003	rs768552222	intronic	c.844-18G>C
CYP2D6	22	42524368	rs370484811	intronic	c.667-16G>A
CYP2D6	22	42523406	rs1261231971	intronic	c.1173+43T>C
CYP2D6	22	42525432	rs1368936023	intronic	c.353-245T>C
CYP2D6	22	42524971	rs573146761	intronic	c.506-25C>T
CYP2D6	22	42526574	rs1449226165	intronic	c.180+40G>A
CYP2D6	22	42525008	rs762326029	intronic	c.505+27G>A
CYP2D6	22	42524014	rs773065145	intronic	c.844-29G>A
CYP2D6	22	42525811	rs28371704	p.His94Arg	c.281A>G
CYP2D6	22	42525821	rs28371703	p.Leu91Met	c.271C>A
CYP2D6	22	42523528	rs1058172	p.Arg365His	c.1094G>A
CYP2D6	22	42526763	rs769258	p.Val11Met	c.31G>A
CYP2D6	22	42524310	rs28371717	p.Ala237Ser	c.709G>T
CYP2D6	22	42524817	rs5030866	p.Gly212Glu	c.635G>A
CYP2D6	22	42522724	rs79392742	p.Ala449Asp	c.1346C>A
CYP2D6	22	42523613	rs78209835	p.Asp337Asn	c.1009G>A
CYP2D6	22	42525077	rs28371710	p.Glu155Lys	c.463G>A
CYP2D6	22	42526717	rs28371696	p.Arg26His	c.77G>A
CYP2D6	22	42526712	rs138100349	p.Arg28Cys	c.82C>T
CYP2D6	22	42526775	rs72549358	p.Val7Met	c.19G>A
CYP2D6	22	42525089	rs78482768	p.Gln151Glu	c.451C>G
CYP2D6	22	42523558	rs202102799	p.Tyr355Cys	c.1064A>G
CYP2D6	22	42524183	rs1135828	p.Met279Lys	c.836T>A
CYP2D6	22	42524878	rs538707090	p.Gly192Arg	c.574G>A
CYP2D6	22	42523567	rs61736517	p.His352Arg	c.1055A>G
CYP2D6	22	42522916	rs28371733	p.Glu418Lys	c.1252G>A
CYP2D6	22	42525035	rs5030865	p.Gly169Arg	c.505G>A
CYP2D6	22	42524187	rs77913725	p.Glu278Lys	c.832G>A
CYP2D6	22	42525176	rs1135823	p.Ala122Ser	c.364G>T
CYP2D6	22	42522754	rs569439709	p.Gly439Asp	c.1316G>A
CYP2D6	22	42522940	rs769157652	p.Glu410Lys	c.1228G>A
CYP2D6	22	42522607	rs568495591	p.Ser488Phe	c.1463C>T
CYP2D6	22	42522601	rs199722016	p.Tyr490Cys	c.1469A>G
CYP2D6	22	42522946	rs78762568	p.Val408Ile	c.1222G>A
CYP2D6	22	42523633	rs141009491	p.Arg330Pro	c.989G>C
CYP2D6	22	42525767	rs78459009	p.Ile109Val	c.325A>G
CYP2D6	22	42524331	rs373813287	p.Leu230Phe	c.688C>T
CYP2D6	22	42525761	rs535642512	p.Gly111Ser	c.331G>A
CYP2D6	22	42525801	rs76802407	p.Asp97Glu	c.291C>G
CYP2D6	22	42523855	rs140513104	p.Pro325Leu	c.974C>T
CYP2D6	22	42526669	rs118203758	p.Gly42Glu	c.125G>A
CYP2D6	22	42523975	rs1135829	p.Asn285Ser	c.854A>G
CYP2D6	22	42524343	rs746803316	p.Ala226Thr	c.676G>A
CYP2D6	22	42524327	rs17002853	p.Leu231Pro	c.692T>C
CYP2D6	22	42522721	rs369177208	p.Arg450His	c.1349G>A
CYP2D6	22	42523483	rs61731586	p.Arg380His	c.1139G>A
CYP2D6	22	42525773	rs74802369	p.Thr107Ser	c.319A>T
CYP2D6	22	42525781	rs76187628	p.Val104Ala	c.311T>C
CYP2D6	22	42523844	rs150216909	p.Arg329Cys	c.985C>T
CYP2D6	22	42522928	rs747089665	p.Arg414Cys	c.1240C>T
CYP2D6	22	42523459	rs77312092	p.Arg388His	c.1163G>A
CYP2D6	22	42522879		p.Pro430Leu	c.1289C>T
CYP2D6	22	42523598	rs750996195	p.Val342Leu	c.1024G>T
CYP2D6	22	42524871	rs369772253	p.Arg194His	c.581G>A
CYP2D6	22	42525044	rs1135824	p.Asn166Asp	c.496A>G
CYP2D6	22	42522684	rs138229048	p.Gln462His	c.1386G>C
CYP2D6	22	42522649	rs141756339	p.Arg474Gln	c.1421G>A
CYP2D6	22	42525136	rs781457579	p.Ser135Phe	c.404C>T
CYP2D6	22	42522586	rs566833518	p.Val495Ala	c.1484T>C
CYP2D6	22	42525115	rs375135093	p.Leu142Ser	c.425T>C
CYP2D6	22	42524917	rs764477422	p.Asp179Asn	c.535G>A
CYP2D6	22	42525172	rs373000587	p.Arg123Leu	c.368G>T
CYP2D6	22	42524884	rs773148044	p.Thr190Ala	c.568A>G
CYP2D6	22	42526649	rs1040704095	p.Val49Met	c.145G>A
CYP2D6	22	42525860	rs775131598	p.Val78Met	c.232G>A
CYP2D6	22	42525118	rs1240215026	p.Asn141Ser	c.422A>G
CYP2D6	22	42524225	rs1341003897	p.Ala265Val	c.794C>T
CYP2D6	22	42524265		p.Asp252Asn	c.754G>A
CYP2D6	22	42524333	rs1252557189	p.Val229Ala	c.686T>C
CYP2D6	22	42522756	rs1232498258	intronic	c.1316-2A>C
CYP2D6	22	42523986		intronic	c.844-1G>A
CYP2D6	22	42525188	rs753911276	intronic	c.353-20_353-2dupCGTGCCCGTCCCACCCCCA
CYP2D6	22	42524353	rs377725912	intronic	c.667-1G>A
CYP2D6	22	42522995	rs1307208772	intronic	c.1174-1G>T
CYP2D6	22	42525738		intronic	c.352+2C>A
CYP2D6	22	42525748	rs765866702	intronic	c.343+1G>A
CYP2D6	22	42523448	rs752858790	intronic	c.1173+1G>T
CYP2D6	22	42525382	rs1174898951	intronic	c.352+1G>A
CYP2D6	22	42524777	rs1270351827	intronic	c.646_666+8delGAGTCGGGCTTTCTGCGCGAGGTGCGGAG
CYP2D6	22	42524784	rs1031847205	intronic	c.666+2T>G
CYP2D6	22	42523447	rs781356352	intronic	c.1173+2T>G
CYP2D6	22	42523448	rs752858790	intronic	c.1173+1G>A
CYP2D6	22	42525390	rs557722765	p.Arg115Arg	c.345C>T
CYP2D6	22	42522760	rs377590656	intronic	c.1316-6C>T
CYP2D6	22	42522759	rs898345711	intronic	c.1316-5C>G
CYP2D6	22	42525036	rs199849357	p.Ser168Ser	c.504C>T
CYP2D6	22	42525193	rs562633069	intronic	c.353-6C>G
CYP2D6	22	42523988	rs373721277	intronic	c.844-3C>G
CYP2D6	22	42524172	rs773264889	intronic	c.843+4A>G
CYP2D6	22	42525918	rs1387103272	intronic	c.181-7T>C
CYP2D6	22	42525195	rs267608305	intronic	c.353-8A>G
CYP2D6	22	42525750	rs370032051	p.Pro114Pro	c.342G>A
CYP2D6	22	42525732		intronic	c.352+8G>T
CYP2D6	22	42524358	rs774751991	intronic	c.667-6G>A
CYP2D6	22	42523000	rs764952493	intronic	c.1174-6G>T
CYP2D6	22	42525190	rs759470167	intronic	c.353-3C>A
CYP2D6	22	42525399	rs1282507444	intronic	c.344-8G>A
CYP2D6	22	42525396	rs773399888	intronic	c.344-5C>G
CYP2D6	22	42525030	rs562436480	intronic	c.505+5G>C
CYP2D6	22	42525379		intronic	c.352+4G>A
CYP2D6	22	42525186	rs267608304	p.Gly118Gly	c.354G>C
CYP2D6	22	42524170	rs769586054	intronic	c.843+6A>C
CYP2D6	22	42524782	rs760526942	intronic	c.666+4C>T
CYP2D6	22	42523446		intronic	c.1173+3A>G
CYP2D6	22	42525390	rs557722765	p.Arg115Arg	c.345C>A
CYP2D6	22	42525384	rs886607240	p.Gln117Gln	c.351G>A
CYP2D6	22	42525375		intronic	c.352+8G>A
CYP2D6	22	42522999		intronic	c.1174-5C>T
CYP2D6	22	42524781	rs752453767	intronic	c.666+5G>A
CYP2D6	22	42523639	rs372154477	intronic	c.986-7_986-4dupTGTC
CYP2D6	22	42525916		intronic	c.181-9_181-6delCCTC
CYP2D6	22	42522758	rs771097393	intronic	c.1316-9_1316-5dupTCCCC
CYP2D6	22	42524949	rs759690407	intronic	c.506-3C>G
CYP2D6	22	42523444		intronic	c.1173+5G>T
CYP2D6	22	42525186	rs267608304	p.Gly118Gly	c.354G>A
CYP2D6	22	42524176	rs749023275	p.Lys281Lys	c.843G>A
CYP2D6	22	42526610	rs766125937	intronic	c.180+4A>G
CYP2D6	22	42523644	rs1373455407	intronic	c.986-8C>T
CYP2D6	22	42524356	rs771359849	intronic	c.667-4G>A
CYP2D6	22	42523639	rs372154477	intronic	c.986-3C>A
CYP2D6	22	42523839	rs767401994	intronic	c.985+5G>A
CYP2D6	22	42523839	rs767401994	intronic	c.985+5G>C
CYP2D6	22	42525395	rs1389437583	intronic	c.344-4A>G
CYP2D6	22	42525377	rs1362845282	intronic	c.352+6C>T
CYP2D6	22	42524358	rs774751991	intronic	c.667-6G>C
CYP2D6	22	42522762	rs1440646871	intronic	c.1316-8C>T
CYP2D6	22	42522758	rs771097393	intronic	c.1316-9_1316-5delTCCCC
CYP2D6	22	42522758	rs771097393	intronic	c.1316-4A>G
CYP2D6	22	42522757	rs1463095582	intronic	c.1316-3C>G
CYP2D6	22	42525919	rs748567457	intronic	c.181-8C>G
CYP2D6	22	42525917	rs1488216942	intronic	c.181-6C>G
CYP2D6	22	42522998	rs1318331960	intronic	c.1174-4C>T
CYP2D6	22	42524952	rs1201123282	intronic	c.506-6C>A
CYP2D6	22	42524168	rs569926140	intronic	c.843+8T>G
CYP2D6	22	42524171	rs1472238599	intronic	c.843+5G>A
CYP2D6	22	42525028	rs755739815	intronic	c.505+7G>A
CYP2D6	22	42524173	rs991361857	intronic	c.843+3G>C
CYP2D6	22	42525030	rs562436480	intronic	c.505+5G>T
CYP2D6	22	42525195	rs267608305	intronic	c.353-8A>T
CYP2D6	22	42525191		intronic	c.353-4C>T
CYP2D6	22	42525031	rs531170800	intronic	c.505+4G>C
CYP2D6	22	42525192	rs767255540	intronic	c.353-5C>A
CYP2D6	22	42525193	rs562633069	intronic	c.353-6C>A
CYP2D6	22	42526607	rs1432475955	intronic	c.180+7G>A
CYP2D6	22	42525734		intronic	c.352+6C>T
CYP2D6	22	42523643	rs549957019	intronic	c.986-7T>C
CYP2D6	22	42523642	rs751897116	intronic	c.986-6G>T
CYP2D6	22	42524360	rs763794858	intronic	c.667-8T>C
CYP2D6	22	42523639	rs372154477	intronic	c.986-3C>T
CYP2D6	22	42524355	rs1368203397	intronic	c.667-3T>G
CYP2D6	22	42523443	rs755243565	intronic	c.1173+6C>G
CYP2D6	22	42523837	rs754895235	intronic	c.985+7C>A
CYP2D6	22	42523845	rs1226011487	p.Gln328Gln	c.984G>A
CYP2D6	22	42523992	rs770112850	intronic	c.844-7T>C
CYP2D6	22	42524214	rs367543000	p.Arg269Ter	c.805C>T
CYP2D6	22	42525035	rs5030865	p.Gly169Ter	c.505G>T
CYP2D6	22	42523941	rs764044673	p.Cys296Ter	c.888C>A
CYP2D6	22	42526640	rs536109057	p.Gln52Ter	c.154C>T
CYP2D6	22	42525168	rs766391487	p.Tyr124Ter	c.372T>G
CYP2D6	22	42525152	rs771490928	p.Glu130Ter	c.388G>T
CYP2D6	22	42524788	rs753945541	p.Glu222Ter	c.664G>T
CYP2D6	22	42523574	rs748850584	p.Gln350Ter	c.1048C>T
CYP2D6	22	42523897	rs1135830	p.Ser311Ter	c.932C>A
CYP2D6	22	42524861	rs375487723	p.Tyr197Ter	c.591C>G
CYP2D6	22	42526616	rs774157537	p.Gln60Ter	c.178C>T
CYP2D6	22	42525149	rs1395910637	p.Gln131Ter	c.391C>T
CYP2D6	22	42526626	rs1386751325	p.Tyr56Ter	c.168C>A
CYP2D6	22	42524818	rs761895610	p.Gly212Ter	c.634G>T
CYP2D6	22	42524802	rs201501394	p.Ser217Ter	c.650C>A
CYP2D6	22	42524806	rs199609589	p.Glu216Ter	c.646G>T
CYP2D6	22	42524233	rs1209113909	p.Trp262Ter	c.786G>A
CYP2D6	22	42523628	rs746824661	p.Gln332Ter	c.994C>T
CYP2D6	22	42523625	rs779957833	p.Gln333Ter	c.997C>T
CYP2D6	22	42523601	rs1230699203	p.Gln341Ter	c.1021C>T
CYP2D6	22	42522576	rs765528990	p.Ter498TyrextTer11	c.1494G>C
CYP2D6	22	42524924	rs111606937	p.Gly176Gly	c.528T>C
CYP2D6	22	42524218	rs28371718	p.Pro267Pro	c.801C>A
CYP2D6	22	42524323	rs17002852	p.His232His	c.696T>C
CYP2D6	22	42522669	rs1135832	p.Ser467Ser	c.1401G>A
CYP2D6	22	42525764	rs527753359	p.Leu110Leu	c.328C>T
CYP2D6	22	42526764	rs561496509	p.Ala10Ala	c.30C>T
CYP2D6	22	42523464	rs771263462	p.Gly386Gly	c.1158C>T
CYP2D6	22	42523956	rs372708040	p.Asn291Asn	c.873T>C
CYP2D6	22	42523887		p.Leu314Leu	c.942G>A
CYP2D6	22	42523923	rs759070183	p.Leu302Leu	c.906G>A
CYP3A4	7	99358459	rs4986913	p.Pro467Ser	c.1399C>T
CYP3A4	7	99358459	rs4986913	p.Pro467Ala	c.1399C>G
CYP3A4	7	99367788	rs72552799	p.Arg130Gln	c.389G>A
CYP3A4	7	99375702	rs56324128	p.Gly56Asp	c.167G>A
CYP3A4	7	99364034	rs4646438	p.Asp277GlufsTer9	c.830dupA
CYP3A4	7	99365994	rs55901263	p.Pro218Arg	c.653C>G
CYP3A4	7	99367825	rs55951658	p.Ile118Val	c.352A>G
CYP3A4	7	99358524	rs4986910	p.Met445Thr	c.1334T>C
CYP3A4	7	99355806	rs67666821	p.Pro488ThrfsTer7	c.1461dupA
CYP3A4	7	99355806	rs67666821	p.Lys487AsnfsTer5	c.1461delA
CYP3A4	7	99365983	rs55785340	p.Ser222Pro	c.664T>C
CYP3A4	7	99367427	rs4986907	p.Arg162Gln	c.485G>A
CYP3A4	7	99359670	rs4986909	p.Pro416Leu	c.1247C>T
CYP3A4	7	99359670	rs4986909	p.Pro416Arg	c.1247C>G
CYP3A4	7	99359800	rs12721629	p.Leu373Phe	c.1117C>T
CYP3A4	7	99359829	rs67784355	p.Thr363Met	c.1088C>T
CYP3A4	7	99359829	rs67784355	p.Thr363Lys	c.1088C>A
CYP3A4	7	99361466	rs2242480	intronic	c.1026+12G>A
CYP3A4	7	99366070	rs3208361	p.Ile193Val	c.577A>G
CYP3A4	7	99367392	rs4986908	p.Asp174His	c.520G>C
CYP3A4	7	99358498	rs750735864	p.Leu454ThrfsTer16	c.1359dupA
CYP3A4	7	99366058	rs773797937	p.Leu196GlnfsTer18	c.587_588delTC
CYP3A4	7	99365999	rs768320602	p.Leu216PhefsTer19	c.647dupT
CYP3A4	7	99361602	rs1199476472	p.Ile300SerfsTer60	c.898_901delATTA
CYP3A4	7	99364832	rs769456534	p.Phe241ValfsTer37	c.719dupT
CYP3A4	7	99381671	rs771463827	p.Trp12GlyfsTer78	c.33delC
CYP3A4	7	99359824	rs1373912108	p.Leu364AspfsTer7	c.1089_1092delGCTC
CYP3A4	7	99364023	rs748261208	p.Ser281GlnfsTer80	c.841delT
CYP3A4	7	99364830	rs945611939	p.Phe241SerfsTer36	c.720_721delGT
CYP3A4	7	99355768	rs760083898	p.Ser501LysfsTer20	c.1499dupT
CYP3A4	7	99355844	rs1281816635	p.Leu475ProfsTer20	c.1423dupC
CYP3A4	7	99364805	rs745882851	p.Leu249PhefsTer29	c.746dupT
CYP3A4	7	99375674	rs756109868	p.His65GlnfsTer2	c.194dupA
CYP3A4	7	99364056	rs1184863262	p.Asp270IlefsTer5	c.808delG
CYP3A4	7	99370249	rs752756881	p.Leu94GlnfsTer2	c.281delT
CYP3A4	7	99370241	rs765569223	p.Glu97AsnfsTer18	c.289delG
CYP3A4	7	99377639	rs1036437989	p.Leu47TrpfsTer43	c.140delT
CYP3A4	7	99377626	rs1263184572	p.Leu51PhefsTer39	c.153delG
CYP3A4	7	99367834	rs1258290270	p.Met114IlefsTer7	c.342delG
CYP3A4	7	99355774	rs761796809	p.Thr499HisfsTer22	c.1493dupG
CYP3A4	7	99355785	rs1476196651	p.Glu494ValfsTer26	c.1481_1482delAG
CYP3A4	7	99367400	rs761291784	p.Thr171AsnfsTer16	c.511dupA
CYP3A4	7	99366033	rs1418257910	p.Glu205LysfsTer7	c.613delG
CYP3A4	7	99367444	rs766618281	p.Leu156PhefsTer31	c.467_468insC
CYP3A4	7	99359734	rs142425279	p.Val394del	c.1180_1182delGTG
CYP3A4	7	99358464	rs767927619	p.Ser464del	c.1391_1393delCCT
CYP3A4	7	99361537	rs1449176667	p.Ala322del	c.964_966delGCC
CYP3A4	7	99358589	rs1475210798	p.Lys422del	c.1266_1268delGAA
CYP3A4	7	99359836	rs1421686291	p.Val360del	c.1078_1080delGTG
CYP3A4	7	99381660	rs1433651188	p.Leu15del	c.42_44delCCT
CYP3A4	7	99361626	rs28371759	p.Leu293Pro	c.878T>C
CYP3A4	7	99367428	rs57409622	p.Arg162Trp	c.484C>T
CYP3A4	7	99367408	rs568779023	p.Lys168Asn	c.504G>C
CYP3A4	7	99359710	rs143966082	p.Arg403Cys	c.1207C>T
CYP3A4	7	99367771	rs748236460	p.Thr136Ala	c.406A>G
CYP3A4	7	99358581	rs139109027	p.Asn426Ser	c.1277A>G
CYP3A4	7	99359845	rs754968125	p.Met358Val	c.1072A>G
CYP3A4	7	99358548	rs1393894592	p.Ser437Thr	c.1310G>C
CYP3A4	7	99364768	rs199908125	p.Glu262Lys	c.784G>A
CYP3A4	7	99377722	rs55671128	p.Val33Leu	c.97G>C
CYP3A4	7	99377740	rs545432563	p.Arg27Cys	c.79C>T
CYP3A4	7	99377748	rs56187094	intronic	c.72-2delA
CYP3A4	7	99375704	rs779398469	intronic	c.166-1G>A
CYP3A4	7	99355852	rs141749477	intronic	c.1417-1G>C
CYP3A4	7	99355852	rs141749477	intronic	c.1417-1G>T
CYP3A4	7	99361477	rs777436556	intronic	c.1026+1G>A
CYP3A4	7	99364752	rs1247641673	intronic	c.798+2T>C
CYP3A4	7	99370212	rs773624444	intronic	c.318+1G>T
CYP3A4	7	99377614	rs758723616	intronic	c.165+1G>A
CYP3A4	7	99370212	rs773624444	intronic	c.318+1G>C
CYP3A4	7	99365976	rs1192964856	intronic	c.670+1G>A
CYP3A4	7	99363998	rs1327039307	intronic	c.865+2T>C
CYP3A4	7	99367744	rs779102038	intronic	c.432+1G>T
CYP3A4	7	99367388	rs773580659	intronic	c.520_521+2delGAGT
CYP3A4	7	99365969	rs55808838	intronic	c.670+8G>A
CYP3A4	7	99358438	rs376112268	intronic	c.1416+4A>G
CYP3A4	7	99367386	rs188546489	intronic	c.521+5G>A
CYP3A4	7	99365969	rs55808838	intronic	c.670+8G>C
CYP3A4	7	99370319	rs1201350386	intronic	c.219-8dupT
CYP3A4	7	99355855	rs1275500416	intronic	c.1417-5delT
CYP3A4	7	99377610	rs200901089	intronic	c.165+5G>A
CYP3A4	7	99366125	rs149271930	p.Asp174Asp	c.522C>T
CYP3A4	7	99359893	rs987337642	intronic	c.1027-3C>G
CYP3A4	7	99359659	rs763248134	intronic	c.1253+5A>G
CYP3A4	7	99359660	rs766366164	intronic	c.1253+4C>G
CYP3A4	7	99375710	rs745489865	intronic	c.166-7C>T
CYP3A4	7	99370320	rs756662615	intronic	c.219-8T>C
CYP3A4	7	99375706	rs1447731963	intronic	c.166-3C>G
CYP3A4	7	99367741	rs771106108	intronic	c.432+4T>C
CYP3A4	7	99375643	rs760671125	intronic	c.218+8T>G
CYP3A4	7	99358437	rs778973808	intronic	c.1416+5G>A
CYP3A4	7	99364884	rs746684076	intronic	c.671-3C>T
CYP3A4	7	99364746	rs760060230	intronic	c.798+8G>A
CYP3A4	7	99375644	rs768757929	intronic	c.218+3_218+6delGAGT
CYP3A4	7	99355858	rs775833385	intronic	c.1417-7G>A
CYP3A4	7	99367482	rs773434059	intronic	c.433-3T>C
CYP3A4	7	99367485	rs771039384	intronic	c.433-6G>A
CYP3A4	7	99377753	rs1355260440	intronic	c.72-6A>G
CYP3A4	7	99377750	rs765248633	intronic	c.72-3A>G
CYP3A4	7	99358610	rs750470018	intronic	c.1254-6A>C
CYP3A4	7	99370316	rs748640574	intronic	c.219-4A>G
CYP3A4	7	99358607	rs79432356	intronic	c.1254-3C>G
CYP3A4	7	99370312	rs781336542	p.Gly73Gly	c.219C>G
CYP3A4	7	99375708	rs1333632870	intronic	c.166-5C>T
CYP3A4	7	99359893	rs987337642	intronic	c.1027-3C>T
CYP3A4	7	99370205	rs1322465944	intronic	c.318+8T>A
CYP3A4	7	99370209	rs770077656	intronic	c.318+4A>G
CYP3A4	7	99367862	rs1352762328	intronic	c.319-4C>G
CYP3A4	7	99377611	rs1220296454	intronic	c.165+4A>G
CYP3A4	7	99361470	rs1210229169	intronic	c.1026+8G>A
CYP3A4	7	99361473	rs1258372443	intronic	c.1026+5G>A
CYP3A4	7	99359888	rs774586724	p.Ala343Ala	c.1029A>T
CYP3A4	7	99377615	rs1355253989	p.Lys55Lys	c.165G>A
CYP3A4	7	99363993	rs752211414	intronic	c.865+7A>T
CYP3A4	7	99363995	rs779091708	intronic	c.865+4delA
CYP3A4	7	99375645	rs1311503222	intronic	c.218+6T>C
CYP3A4	7	99375644	rs768757929	intronic	c.218+7A>C
CYP3A4	7	99367385	rs775328500	intronic	c.521+6T>G
CYP3A4	7	99366133	rs1478194349	intronic	c.522-8C>T
CYP3A4	7	99367487	rs1380578959	intronic	c.433-8G>C
CYP3A4	7	99381626	rs745626198	intronic	c.71+8A>C
CYP3A4	7	99359657	rs768470122	intronic	c.1253+7G>A
CYP3A4	7	99367483	rs749476253	intronic	c.433-4T>G
CYP3A4	7	99359658	rs773371203	intronic	c.1253+6A>T
CYP3A4	7	99364063	rs138105638	p.Arg268Ter	c.802C>T
CYP3A4	7	99364806	rs369797181	p.Leu249Ter	c.746T>A
CYP3A4	7	99358444	rs780449767	p.Gln472Ter	c.1414C>T
CYP3A4	7	99359694	rs368005287	p.Trp408Ter	c.1223G>A
CYP3A4	7	99367789	rs778013004	p.Arg130Ter	c.388C>T
CYP3A4	7	99359696	rs1348112778	p.Tyr407Ter	c.1221C>A
CYP3A4	7	99364845	rs1251636473	p.Leu236Ter	c.707T>G
CYP3A4	7	99375682	rs752813465	p.Glu63Ter	c.187G>T
CYP3A4	7	99359863	rs1431067941	p.Gln352Ter	c.1054C>T
CYP3A4	7	99355832	rs1350383629	p.Leu479Ter	c.1436T>A
CYP3A4	7	99366049	rs1425657444	p.Gln200Ter	c.598C>T
CYP3A4	7	99355757	rs772460209	p.Ter504Ter	c.1511G>A
CYP3A5	7	99270222	rs41279857	p.Ser100Tyr	c.299C>A
CYP3A5	7	99273815	rs28383468	p.His30Tyr	c.88C>T
CYP3A5	7	99258139	rs28383479	p.Ala337Thr	c.1009G>A
CYP3A5	7	99273821	rs55817950	p.Arg28Cys	c.82C>T
CYP3A5	7	99250393	rs41303343	p.Thr346TyrfsTer3	c.1035dupT
CYP3A5	7	99250393	rs41303343	p.Thr346Ala	c.1036A>G
CYP3A5	7	99262835	rs10264272	p.Lys208Lys	c.624G>A
CYP3A5	7	99262860	rs56411402	p.Gln200Arg	c.599A>G
CYP3A5	7	99245974	rs28365085	p.Ile488Thr	c.1463T>C
CYP3A5	7	99270276	rs56244447	p.Leu82Arg	c.245T>G
CYP3A5	7	99250236	rs28365083	p.Thr398Asn	c.1193C>A
CYP3A5	7	99273745	rs72552791	p.Tyr53Cys	c.158A>G
CYP3A5	7	99247772	rs41279854	p.Phe446Ser	c.1337T>C
CYP3A5	7	99273810	rs200579169	p.Leu32ThrfsTer3	c.92dupG
CYP3A5	7	99247736	rs547253411	p.Val458SerfsTer17	c.1372delG
CYP3A5	7	99270273	rs753330469	p.Ala83GlyfsTer40	c.247dupG
CYP3A5	7	99264598	rs767317522	p.Phe137SerfsTer21	c.408delC
CYP3A5	7	99269464	rs1487538886	p.His119IlefsTer73	c.355delC
CYP3A5	7	99245941	rs1278901170	p.Leu499Ter	c.1495delC
CYP3A5	7	99277505	rs753526701	p.Pro5GlnfsTer85	c.14delC
CYP3A5	7	99264253	rs994632442	p.Ala164GlufsTer9	c.491delC
CYP3A5	7	99250371	rs748831637	p.Pro345TrpfsTer8	c.1033_1057delCCTACCTATGATGCCGTGGTACAGA
CYP3A5	7	99262870	rs1234678621	p.Leu196GlnfsTer12	c.587_588delTC
CYP3A5	7	99250340	rs755871374	p.Thr363AsnfsTer33	c.1088delC
CYP3A5	7	99247736	rs547253411	p.Arg457SerfsTer11	c.1371_1372delAG
CYP3A5	7	99269410	rs1313225219	p.Cys137ValfsTer55	c.409delT
CYP3A5	7	99277497	rs369170873	p.Ala8ArgfsTer82	c.22delG
CYP3A5	7	99261695	rs772471367	p.Pro231ArgfsTer3	c.692_693delCA
CYP3A5	7	99261612	rs1355546883	p.Lys257SerfsTer18	c.769_776delAAGAAAAG
CYP3A5	7	99261669	rs748669600	p.Leu240ValfsTer11	c.718_719delCT
CYP3A5	7	99245940	rs760945831	p.Ser500LysfsTer30	c.1496dupT
CYP3A5	7	99272187	rs1442558737	p.Thr62ArgfsTer4	c.185_186delCA
CYP3A5	7	99250368	rs779671620	p.Glu354GlyfsTer4	c.1060dupG
CYP3A5	7	99273824	rs1252656182	p.Thr27ProfsTer63	c.78delG
CYP3A5	7	99270206	rs766144904	p.Arg105GlnfsTer4	c.314delG
CYP3A5	7	99273803	rs1407515047	p.Phe33Ter	c.98_99delTT
CYP3A5	7	99247696	rs762834225	p.Gln471AspfsTer58	c.1411_1412delCA
CYP3A5	7	99258246	rs1218326641	p.Ile301SerfsTer60	c.901delA
CYP3A5	7	99250289	rs1387559334	p.Asp380ValfsTer16	c.1139delA
CYP3A5	7	99264234	rs765303708	p.Thr171TyrfsTer38	c.510_511insT
CYP3A5	7	99262923	rs771170846	p.Tyr179ThrfsTer5	c.535delT
CYP3A5	7	99247721	rs1170602456	p.Phe462del	c.1385_1387delTCT
CYP3A5	7	99247839	rs1420059633	p.Lys423del	c.1267_1269delAAG
CYP3A5	7	99261602	rs761366357	p.Asn262del	c.784_786delAAC
CYP3A5	7	99250357	rs369060445	p.Leu356_Asp357del	c.1066_1071delCTTGAC
CYP3A5	7	99264635	rs750274831	p.Asp123dup	c.369_371dupTGA
CYP3A5	7	99269401	rs6957030	p.Leu140Arg	c.419T>G
CYP3A5	7	99258153	rs188366390	p.Gln332Pro	c.995A>C
CYP3A5	7	99247802	rs188908808	p.Thr436Ser	c.1307C>G
CYP3A5	7	99250318	rs28365092	p.Ile371Val	c.1111A>G
CYP3A5	7	99260477	rs145774441	p.Ile276Thr	c.827T>C
CYP3A5	7	99250365	rs149888520	p.Tyr355Cys	c.1064A>G
CYP3A5	7	99262897	rs202148429	p.Ser188Thr	c.562T>A
CYP3A5	7	99262851	rs147489136	p.Phe203Cys	c.608T>G
CYP3A5	7	99273743	rs566629410	p.Arg54Gly	c.160C>G
CYP3A5	7	99264299	rs142823108	p.Ile149Thr	c.446T>C
CYP3A5	7	99264313	rs373134805	intronic	c.433-1G>C
CYP3A5	7	99250403	rs767785315	intronic	c.1027-2delA
CYP3A5	7	99264690	rs1431404870	intronic	c.319-2A>G
CYP3A5	7	99274201	rs1324019660	intronic	c.72-2A>G
CYP3A5	7	99250403	rs767785315	intronic	c.1027-1G>C
CYP3A5	7	99247856	rs767277330	intronic	c.1254-4_1254-2delCTA
CYP3A5	7	99262939	rs1355793763	intronic	c.522-2A>G
CYP3A5	7	99264573	rs55965422	intronic	c.432+2T>C
CYP3A5	7	99272154	rs1404335864	intronic	c.218+2T>A
CYP3A5	7	99264574	rs759812876	intronic	c.432+1G>T
CYP3A5	7	99258120	rs746690864	intronic	c.1026+2T>C
CYP3A5	7	99272155	rs1382315230	intronic	c.218+1G>T
CYP3A5	7	99270202	rs747171434	intronic	c.318+1G>A
CYP3A5	7	99264574	rs759812876	intronic	c.432+1G>A
CYP3A5	7	99277448	rs1278659430	intronic	c.71+1G>A
CYP3A5	7	99246026	rs28365069	intronic	c.1414-3T>C
CYP3A5	7	99273942	rs115267978	p.Ile13Ile	c.39C>T
CYP3A5	7	99277444	rs28371766	intronic	c.71+5G>A
CYP3A5	7	99262785	rs377523033	intronic	c.670+4T>C
CYP3A5	7	99277436	rs760552678	intronic	c.71+3_71+12delGAGTAACTGT
CYP3A5	7	99269506	rs188854304	intronic	c.319-5C>T
CYP3A5	7	99264218	rs112257049	intronic	c.521+6T>G
CYP3A5	7	99260511	rs188843385	intronic	c.799-6T>C
CYP3A5	7	99264315	rs1397463176	intronic	c.433-3T>C
CYP3A5	7	99264219	rs759623461	intronic	c.521+4delA
CYP3A5	7	99262944	rs199704389	intronic	c.522-7C>T
CYP3A5	7	99273734	rs756323485	intronic	c.165+4A>C
CYP3A5	7	99269506	rs188854304	intronic	c.319-5C>G
CYP3A5	7	99269508	rs904198743	intronic	c.319-7T>C
CYP3A5	7	99270196	rs780286142	intronic	c.318+7A>G
CYP3A5	7	99272212	rs372154369	intronic	c.166-4C>T
CYP3A5	7	99269505	rs1234857112	intronic	c.319-4G>A
CYP3A5	7	99270305	rs551764170	intronic	c.219-3C>A
CYP3A5	7	99264571	rs1046659243	intronic	c.432+4T>G
CYP3A5	7	99277442	rs1029727643	intronic	c.71+3_71+6delGAGT
CYP3A5	7	99258119	rs779631885	intronic	c.1026+3G>A
CYP3A5	7	99247858	rs770461230	intronic	c.1254-3T>A
CYP3A5	7	99258289	rs1172280150	intronic	c.866-7C>T
CYP3A5	7	99273831	rs762933090	p.Leu24Leu	c.72A>C
CYP3A5	7	99264318	rs1196779314	intronic	c.433-6G>A
CYP3A5	7	99264218	rs112257049	intronic	c.521+6T>C
CYP3A5	7	99269508	rs904198743	intronic	c.319-7T>G
CYP3A5	7	99269499	rs184167131	p.Ile107Ile	c.321T>C
CYP3A5	7	99262784	rs1272366132	intronic	c.670+5G>A
CYP3A5	7	99260511	rs188843385	intronic	c.799-6T>G
CYP3A5	7	99260509	rs1396475781	intronic	c.799-5delT
CYP3A5	7	99261721	rs1235155545	intronic	c.671-3C>T
CYP3A5	7	99261587	rs1455060067	intronic	c.798+4A>C
CYP3A5	7	99273936		intronic	c.41+4C>T
CYP3A5	7	99261586	rs761169488	intronic	c.798+5A>C
CYP3A5	7	99261583	rs775705711	intronic	c.798+8C>A
CYP3A5	7	99274204	rs773892364	intronic	c.72-5C>T
CYP3A5	7	99250173	rs1304051811	intronic	c.1253+3A>G
CYP3A5	7	99246031	rs750111243	intronic	c.1414-8T>A
CYP3A5	7	99246030	rs1273255499	intronic	c.1414-7A>G
CYP3A5	7	99262781	rs745871984	intronic	c.670+8G>A
CYP3A5	7	99262781	rs745871984	intronic	c.670+8G>T
CYP3A5	7	99262783	rs780382113	intronic	c.670+6T>C
CYP3A5	7	99258122	rs754660849	p.Lys342Lys	c.1026G>A
CYP3A5	7	99264692	rs769904092	intronic	c.319-4C>G
CYP3A5	7	99270326	rs771525579	intronic	c.*599-5A>G
CYP3A5	7	99270321	rs1159139302	intronic	c.*599C>T
CYP3A5	7	99272211	rs1221265115	intronic	c.166-3C>T
CYP3A5	7	99273730	rs1260265514	intronic	c.165+8G>C
CYP3A5	7	99273831	rs762933090	p.Leu24Leu	c.72A>G
CYP3A5	7	99264571	rs1046659243	intronic	c.432+4T>C
CYP3A5	7	99277446	rs756457940	intronic	c.71+3G>C
CYP3A5	7	99260435	rs773263948	intronic	c.865+4A>G
CYP3A5	7	99262945	rs1286030745	intronic	c.522-8C>T
CYP3A5	7	99277517	rs745452294	p.Met1?	c.3G>C
CYP3A5	7	99261643	rs201260783	p.Leu249Ter	c.746T>A
CYP3A5	7	99269425	rs559561358	p.Ser132Ter	c.395C>G
CYP3A5	7	99247731	rs149664815	p.Gln460Ter	c.1378C>T
CYP3A5	7	99260502	rs148176345	p.Arg268Ter	c.802C>T
CYP3A5	7	99272158	rs895150745	p.Trp72Ter	c.216G>A
CYP3A5	7	99264289	rs561555514	p.Tyr152Ter	c.456T>G
CYP3A5	7	99260451	rs774855888	p.Glu285Ter	c.853G>T
CYP3A5	7	99270208	rs776567184	p.Arg105Ter	c.313C>T
CYP3A5	7	99272159	rs1172946988	p.Trp72Ter	c.215G>A
CYP3A5	7	99250345	rs1448299493	p.Glu362Ter	c.1084G>T
CYP3A5	7	99250332	rs757522026	p.Leu366Ter	c.1097T>A
CYP3A5	7	99269409	rs1257468120	p.Cys137Ter	c.411T>A
CYP3A5	7	99261596	rs763775338	p.Gln265Ter	c.793C>T
CYP3A5	7	99272157	rs781230602	p.Gly73Ter	c.217G>T
CYP3A5	7	99273740	rs1213117276	p.Gln55Ter	c.163C>T
CYP3A5	7	99270232	rs763263522	p.Glu97Ter	c.289G>T
CYP3A5	7	99277451	rs777442533	p.Tyr23Ter	c.69T>A
CYP3A5	7	99260462	rs111371159	p.Ser281Ter	c.842C>A
CYP3A5	7	99277485	rs1288443539	p.Trp12Ter	c.35G>A
CYP3A5	7	99269397	rs6977165	p.Ter141TrpextTer12	c.423A>G
CYP3A5	7	99274153	rs755590217	p.Ter40GlnextTer31	c.118T>C
FMO3	1	171077323	rs780358952	p.Cys197Ter	c.591_592delTG
FMO3	1	171083456	rs775064534	p.Pro380HisfsTer3	c.1139_1140delCC
FMO3	1	171072947	rs72549321	p.Ala52ValfsTer23	c.155_162delCTAGCATT
FMO3	1	171072964	rs1203286290	p.Val58GlnfsTer17	c.172_179delGTCTTTTC
FMO3	1	171072982	rs1331111339	p.Lys64ArgfsTer13	c.191_192delAA
FMO3	1	171072976	rs1267874007	p.Ser62LeufsTer5	c.184delT
FMO3	1	171072958	rs1307554902	p.Lys56IlefsTer21	c.167_168delAA
FMO3	1	171072974	rs1223166427	p.Asn61ArgfsTer7	c.181_182insGG
FMO3	1	171083309	rs575739239	p.Tyr331Ter	c.993_994delTA
FMO3	1	171083500	rs754992629	intronic	c.1183+1delG
FMO3	1	171076882	rs866756635	p.Arg131GlyfsTer13	c.391delA
FMO3	1	171077332	rs1411667661	p.Ala200ProfsTer12	c.598delG
FMO3	1	171086580	rs542162053	p.Ter533Tyr	c.1599delA
FMO3	1	171086503	rs781468590	p.Lys507AsnfsTer9	c.1521delG
FMO3	1	171085397	rs763690279	p.Met413AsnfsTer24	c.1237dupA
FMO3	1	171083327	rs763369659	p.Leu338ProfsTer2	c.1010dupT
FMO3	1	171073026	rs777700920	p.Asn80ThrfsTer22	c.237delC
FMO3	1	171073015	rs755554412	p.Asp76ArgfsTer2	c.225dupC
FMO3	1	171077293	rs1270775688	p.Val187SerfsTer25	c.559delG
FMO3	1	171077313	rs116425430	p.Asn194PhefsTer4	c.579_580delGA
FMO3	1	171083492	rs1285545984	p.Val392Ter	c.1174delG
FMO3	1	171083436	rs767079173	p.Ser374ValfsTer10	c.1118dupA
FMO3	1	171085406	rs769188556	p.Lys416AlafsTer20	c.1246_1247delAA
FMO3	1	171076825	rs757188439	p.Ser112GlnfsTer4	c.333dupC
FMO3	1	171086404	rs747184293	p.Gly475AlafsTer13	c.1424delG
FMO3	1	171086420	rs755216654	p.Pro480GlnfsTer8	c.1439delC
FMO3	1	171083253	rs770309578	p.Glu314Ter	c.939dupT
FMO3	1	171086273		p.Ile431AsnfsTer6	c.1291dupA
FMO3	1	171080036	rs1331483649	p.Phe242LeufsTer15	c.726_729delCCTC
FMO3	1	171077286	rs745663963	p.Lys185SerfsTer27	c.554delA
FMO3	1	171083207	rs775563975	p.Ser298_Val299del	c.891_896delGTCCGT
FMO3	1	171079983	rs768811406	p.Asn227_Asp232del	c.680_697delATGGTTATCCTTGGGACA
FMO3	1	171076966	rs2266782	p.Glu158Lys	c.472G>A
FMO3	1	171083242	rs2266780	p.Glu308Gly	c.923A>G
FMO3	1	171080080	rs1736557	p.Val257Met	c.769G>A
FMO3	1	171077274	rs75904274	p.Gly180Val	c.539G>T
FMO3	1	171083149	rs2066530	p.Val277Ala	c.830T>C
FMO3	1	171076888	rs12072582	p.Asp132His	c.394G>C
FMO3	1	171083403	rs2066532	p.Glu362Gln	c.1084G>C
FMO3	1	171076952	rs72549326	p.Pro153Leu	c.458C>T
FMO3	1	171086488	rs60306057	p.Val502Gly	c.1505T>G
FMO3	1	171080017	rs201271626	p.Val236Ile	c.706G>A
FMO3	1	171076835	rs186763441	p.Asn114Ser	c.341A>G
FMO3	1	171083398	rs28363581	p.Leu360Pro	c.1079T>C
FMO3	1	171070298	rs151271991	p.Gln8Arg	c.23A>G
FMO3	1	171085386	rs141280604	p.Asp408Tyr	c.1222G>T
FMO3	1	171072965	rs144935285	p.Val58Ile	c.172G>A
FMO3	1	171085413	rs149551557	p.Arg417Cys	c.1249C>T
FMO3	1	171086461	rs61008738	p.Ser493Leu	c.1478C>T
FMO3	1	171076823	rs376881697	p.Val110Ala	c.329T>C
FMO3	1	171085368	rs141117096	p.Met402Val	c.1204A>G
FMO3	1	171077347	rs753360820	p.Ser204Arg	c.612C>A
FMO3	1	171077348	rs28363549	p.Arg205Cys	c.613C>T
FMO3	1	171077329	rs529940450	p.Asp198Glu	c.594T>A
FMO3	1	171085414	rs200985584	p.Arg417His	c.1250G>A
FMO3	1	171073021	rs199766869	p.Asp76Glu	c.228T>A
FMO3	1	171083479	rs72549331	p.Arg387His	c.1160G>A
FMO3	1	171086457	rs72549334	p.Arg492Trp	c.1474C>T
FMO3	1	171083248	rs572292275	p.Ser310Leu	c.929C>T
FMO3	1	171077292	rs143406401	p.Arg186His	c.557G>A
FMO3	1	171086482	rs183949390	p.Arg500Gln	c.1499G>A
FMO3	1	171079978	rs762600525	p.Arg223Trp	c.667C>T
FMO3	1	171076936	rs72549325	p.Gly148Arg	c.442G>A
FMO3	1	171072975	rs72549322	p.Asn61Ser	c.182A>G
FMO3	1	171072991	rs72549323	p.Met66Ile	c.198G>T
FMO3	1	171072947	rs72549321	p.Ala52Thr	c.154G>A
FMO3	1	171077218	rs1316124316	intronic	c.485-2A>G
FMO3	1	171072924	rs760256450	intronic	c.133-2A>G
FMO3	1	171077256	rs778745952	intronic	c.468-1G>C
FMO3	1	171083145		intronic	c.828-2A>T
FMO3	1	171077255	rs201459120	intronic	c.468-2A>G
FMO3	1	171079938	rs779056077	intronic	c.628-1G>A
FMO3	1	171061793		intronic	c.-6-1G>C
FMO3	1	171070348	rs1306918384	intronic	c.72+1G>A
FMO3	1	171076963	rs1346177329	intronic	c.467+2A>G
FMO3	1	171077363	rs758201900	intronic	c.627+1G>A
FMO3	1	171073115	rs756477043	intronic	c.321+1G>T
FMO3	1	171080140	rs1388265038	intronic	c.827+2T>A
FMO3	1	171077363	rs758201900	intronic	c.627+1G>T
FMO3	1	171076979	rs778868969	intronic	c.484+1G>A
FMO3	1	171061932	rs886038576	intronic	c.132+1G>A
FMO3	1	171079933	rs369396459	intronic	c.628-6T>C
FMO3	1	171070213	rs1460473866	intronic	c.-55-8A>G
FMO3	1	171072922	rs1345311094	intronic	c.133-4A>T
FMO3	1	171061934	rs1023133285	intronic	c.132+3G>C
FMO3	1	171085425	rs1365259746	intronic	c.1256+5G>C
FMO3	1	171076813	rs996915425	intronic	c.322-3T>G
FMO3	1	171061794	rs932053102	intronic	c.-6G>C
FMO3	1	171076809	rs762847643	intronic	c.322-7C>A
FMO3	1	171070352	rs771437682	intronic	c.72+5T>C
FMO3	1	171070217	rs904926158	intronic	c.-55-4C>A
FMO3	1	171079934	rs755166406	intronic	c.628-5T>C
FMO3	1	171061789	rs1279445358	intronic	c.-6-5C>A
FMO3	1	171061790	rs1485989862	intronic	c.-6-4A>G
FMO3	1	171086234	rs367722591	intronic	c.1257-6C>T
FMO3	1	171080144	rs752917120	intronic	c.827+6G>T
FMO3	1	171076983	rs748055020	intronic	c.484+5G>A
FMO3	1	171070217	rs904926158	intronic	c.-55-4C>T
FMO3	1	171070215	rs1335797605	intronic	c.-55-6G>A
FMO3	1	171070353	rs758416447	intronic	c.72+6G>A
FMO3	1	171070354	rs1393187212	intronic	c.72+7T>A
FMO3	1	171083507	rs753668825	intronic	c.1183+5G>A
FMO3	1	171083505	rs1184684149	intronic	c.1183+3A>C
FMO3	1	171085423	rs759297147	intronic	c.1256+3A>T
FMO3	1	171076808	rs1320359252	intronic	c.322-8T>C
FMO3	1	171085336	rs753324854	intronic	c.1184-9_1184-6delCTTT
FMO3	1	171077368	rs777725103	intronic	c.627+6A>C
FMO3	1	171086233	rs1205377073	intronic	c.1257-7T>C
FMO3	1	171072921	rs772849886	intronic	c.133-5C>G
FMO3	1	171076968	rs1209929066	intronic	c.467+7G>A
FMO3	1	171077251	rs1426963043	intronic	c.468-6C>T
FMO3	1	171061938	rs751323865	intronic	c.132+7A>C
FMO3	1	171083232	rs61753344	p.Glu305Ter	c.913G>T
FMO3	1	171077357	rs559643079	p.Glu208Ter	c.622G>T
FMO3	1	171086390	rs1409513434	p.Tyr469Ter	c.1407C>A
FMO3	1	171086481	rs765373503	p.Arg500Ter	c.1498C>T
FMO3	1	171080023	rs893223321	p.Arg238Ter	c.712C>T
FMO3	1	171076864	rs1384237868	p.Gln124Ter	c.370C>T
FMO3	1	171076900	rs780703747	p.Glu136Ter	c.406G>T
FMO3	1	171079968	rs747533670	p.Trp219Ter	c.657G>A
FMO3	1	171080003	rs1198164523	p.Trp231Ter	c.692G>A
FMO3	1	171083490	rs1215716992	p.Gln391Ter	c.1171C>T
FMO3	1	171083483	rs750002345	p.Trp388Ter	c.1164G>A
FMO3	1	171086525	rs772961889	p.Trp514Ter	c.1542G>A
FMO3	1	171076936	rs72549325	p.Gly148Ter	c.442G>T
FMO3	1	171083259	rs72549330	p.Glu314Ter	c.940G>T
FMO3	1	171073049	rs1331640570	p.Lys86Ter	c.256A>T
FMO3	1	171086253	rs751472166	p.Glu424Ter	c.1270G>T
FMO3	1	171086580	rs542162053	p.Ter533GlnextTer24	c.1597T>C
FMO3	1	171086513	rs28363595	p.Phe510Phe	c.1530C>T
UGT1A1	2	234638282	rs544798573	p.Leu173PhefsTer7	c.518dupT
UGT1A1	2	234622331	rs1039930586	p.Ala232GlufsTer31	c.695delC
UGT1A1	2	234638205	rs758464251	p.Phe146LeufsTer2	c.435dupC
UGT1A1	2	234621776	rs768171102	p.Leu48CysfsTer18	c.141delC
UGT1A1	2	234637898	rs761976281	p.Ser43AlafsTer23	c.127delA
UGT1A1	2	234638434	rs377448970	p.Tyr222Ter	c.665_708delACATTTGCCATGCTTTTTCTGCTCCTTATGCAAGCCTTGCCTCT
UGT1A1	2	234581434	rs753335727	intronic	c.855+1delG
UGT1A1	2	234581259	rs1302383027	p.Leu228Ter	c.682delC
UGT1A1	2	234621763	rs1395487296	p.Ser43AlafsTer23	c.127delA
UGT1A1	2	234676999	rs1224310774	p.Lys408ArgfsTer5	c.1223delA
UGT1A1	2	234581153	rs1491389239	p.Arg192AsnfsTer41	c.575_576delGA
UGT1A1	2	234581158	rs1020018946	p.Ile193MetfsTer4	c.578_579insGA
UGT1A1	2	234638483	rs774024248	p.Leu238PhefsTer25	c.712delC
UGT1A1	2	234638227	rs200733466	p.Val154ArgfsTer2	c.459dupC
UGT1A1	2	234580937	rs773627969	p.Ser122PhefsTer8	c.364dupT
UGT1A1	2	234669010	rs775463336	p.Ala27ValfsTer25	c.80_93delCTGGGAAGATACTG
UGT1A1	2	234622143	rs773152054	p.Phe172LeufsTer7	c.510_511delGT
UGT1A1	2	234638529	rs1220265805	p.Ser253LeufsTer10	c.758delC
UGT1A1	2	234676539	rs745655794	p.Asn349ThrfsTer18	c.1046delA
UGT1A1	2	234580936	rs1457099532	p.Leu119PhefsTer15	c.357delA
UGT1A1	2	234669554	rs766536479	p.Arg209ProfsTer50	c.622_625dupCAGC
UGT1A1	2	234677001	rs758192306	p.Ala410SerfsTer13	c.1226dupG
UGT1A1	2	234526356	rs1237258590	p.Arg3GlnfsTer15	c.6_9delTCGC
UGT1A1	2	234680955	rs114982090	p.Val453SerfsTer13	c.1357_1360delGTGG
UGT1A1	2	234526926	rs773647810	p.Arg192AsnfsTer41	c.575_576delGA
UGT1A1	2	234526931	rs762517651	p.Ile193MetfsTer4	c.578_579insGA
UGT1A1	2	234526380	rs766968241	p.Ile10AsnfsTer15	c.28dupA
UGT1A1	2	234638461	rs749084156	p.Tyr231LeufsTer7	c.691dupT
UGT1A1	2	234580892	rs747225870	p.Asn114IlefsTer4	c.316_338dupTATTCTCTATTAATGGGTTCATA
UGT1A1	2	234580855	rs781611946	p.Phe93LeufsTer17	c.279delT
UGT1A1	2	234526710	rs1413542776	p.Ser122ArgfsTer12	c.364delT
UGT1A1	2	234669280	rs140867457	p.Asp119GlyfsTer28	c.353dupA
UGT1A1	2	234638217	rs780418650	p.Leu150PhefsTer6	c.449dupT
UGT1A1	2	234637841	rs1363625359	p.Trp25GlyfsTer41	c.72delC
UGT1A1	2	234677080	rs754549295	p.Ser436LysfsTer74	c.1306dupA
UGT1A1	2	234676499	rs757687307	p.Trp336GlyfsTer31	c.1005delG
UGT1A1	2	234580603	rs747116611	p.Leu10ProfsTer45	c.28dupC
UGT1A1	2	234681119	rs749363673	p.Phe508LeufsTer38	c.1521delC
UGT1A1	2	234581410	rs759091845	p.His278ProfsTer86	c.831_832dupCC
UGT1A1	2	234622486	rs149208140	p.Gly284LeufsTer84	c.849_850insTT
UGT1A1	2	234680992	rs115944950	p.Phe465LeufsTer2	c.1395delT
UGT1A1	2	234580918	rs1198724150	p.Asn114GlnfsTer2	c.339dupC
UGT1A1	2	234638282	rs544798573	p.Leu173Ter	c.518delT
UGT1A1	2	234638080	rs750763641	p.Glu104ThrfsTer22	c.310_311delGA
UGT1A1	2	234526957	rs1486595302	p.Thr202IlefsTer27	c.605delC
UGT1A1	2	234638078	rs1456497728	p.Thr103ArgfsTer23	c.308_309delCA
UGT1A1	2	234527025	rs1199387223	p.Asn226MetfsTer3	c.677delA
UGT1A1	2	234527023	rs768137865	p.Asn226MetfsTer3	c.672delC
UGT1A1	2	234527023	rs768137865	p.Ser224Ter	c.671_672delCC
UGT1A1	2	234527096	rs1234498308	p.Ser249GlnfsTer17	c.744delA
UGT1A1	2	234527017	rs1372746314	p.Tyr222PhefsTer7	c.665delA
UGT1A1	2	234527018	rs762421458	p.Phe223LeufsTer7	c.666_667insCT
UGT1A1	2	234527015	rs139633511	p.Gln221HisfsTer9	c.662_663insCC
UGT1A1	2	234527072	rs1282094999	p.Ala241GlufsTer26	c.719_720insGG
UGT1A1	2	234526992	rs765736564	p.Leu214PhefsTer20	c.641dupT
UGT1A1	2	234527074	rs745519607	p.Ala241ValfsTer2	c.722_723delCA
UGT1A1	2	234638313	rs1318078089	p.Asp181GlufsTer3	c.542dupA
UGT1A1	2	234638318	rs769537040	p.Lys183AlafsTer15	c.547_550delAAGG
UGT1A1	2	234622260	rs1421583366	p.Gln209LysfsTer54	c.624delG
UGT1A1	2	234638368	rs1402842661	p.Thr200ProfsTer6	c.598delA
UGT1A1	2	234622219	rs766446503	p.Arg196Ter	c.584dupC
UGT1A1	2	234622230	rs1250921836	p.Thr200AsnfsTer39	c.595_598dupACGA
UGT1A1	2	234669648	rs1245371684	p.Glu241GlyfsTer16	c.722_723delAG
UGT1A1	2	234638518	rs1341681741	p.His251CysfsTer41	c.747_748delCA
UGT1A1	2	234580842	rs143487779	p.Glu89SerfsTer21	c.265delG
UGT1A1	2	234580854	rs1276219034	p.Ala92ValfsTer18	c.275delC
UGT1A1	2	234622162	rs762930324	p.Ile176SerfsTer3	c.526_527delAT
UGT1A1	2	234638251	rs755451015	p.Ala162SerfsTer11	c.483_486delGGCT
UGT1A1	2	234669449	rs748734877	p.His173MetfsTer32	c.517delC
UGT1A1	2	234638566	rs776734052	p.Arg267GlyfsTer100	c.798delC
UGT1A1	2	234669025	rs1308601724	p.Leu32Ter	c.93delG
UGT1A1	2	234638584	rs1215898603	p.Asn272ThrfsTer96	c.812_813insGA
UGT1A1	2	234526406	rs752937950	p.Thr19LeufsTer5	c.54_55delGA
UGT1A1	2	234526503	rs747161771	p.Arg51GlyfsTer3	c.151_152delAG
UGT1A1	2	234526573	rs777095493	p.Lys75GlufsTer44	c.221dupT
UGT1A1	2	234637941	rs765475484	p.Ala58GlyfsTer31	c.170dupA
UGT1A1	2	234621808	rs776401165	p.Val60TrpfsTer8	c.173_177dupTGGTG
UGT1A1	2	234637858	rs1402796728	p.Lys30ArgfsTer36	c.89delA
UGT1A1	2	234669504	rs1196570380	p.Tyr192ThrfsTer13	c.573delC
UGT1A1	2	234669532	rs1453639780	p.Ser201GlnfsTer4	c.601delT
UGT1A1	2	234622034	rs1287964776	p.His133GlnfsTer2	c.398dupA
UGT1A1	2	234622043	rs765055353	p.Leu137ProfsTer11	c.409dupC
UGT1A1	2	234637840	rs1295015456	p.Pro24_Val31del	c.70_93delCCCTGGGCTGAGAGTGGAAAGGTG
UGT1A1	2	234669440	rs1236416699	p.Phe171del	c.513_515delCTT
UGT1A1	2	234676520	rs780354743	p.Pro343del	c.1027_1029delCCA
UGT1A1	2	234681127	rs771159962	p.Cys511del	c.1531_1533delTGT
UGT1A1	2	234669657	rs759864220	p.Val242_Thr243delinsAla	c.725_727delTGA
UGT1A1	2	234580897	rs748508772	p.Ser107del	c.319_321delTCT
UGT1A1	2	234638503	rs759461021	p.Val246del	c.732_734delAGT
UGT1A1	2	234580853	rs1210122752	p.Phe93_Ala94del	c.276_281delTTTTGC
UGT1A1	2	234526851	rs770311347	p.Val167_Phe168del	c.500_505delTCTTCG
UGT1A1	2	234621951	rs761507156	p.Leu107del	c.318_320delTCT
UGT1A1	2	234668879	rs34983651	intronic	c.868-6787_868-6786dupTA
UGT1A1	2	234668879	rs34983651	intronic	c.868-6787_868-6786delTA
UGT1A1	2	234675826	rs4148327	intronic	c.999+15T>C
UGT1A1	2	234669144	rs4148323	p.Gly71Arg	c.211G>A
UGT1A1	2	234675779	rs200903749	p.Ile323Val	c.967A>G
UGT1A1	2	234669619	rs35350960	p.Pro229Gln	c.686C>A
UGT1A1	2	234676872	rs34946978	p.Pro365Leu	c.1094C>T
UGT1A1	2	234669607	rs35003977	p.Val225Gly	c.674T>G
UGT1A1	2	234680955	rs114982090	p.Pro452Leu	c.1355C>T
UGT1A1	2	234669259	rs144217005	p.Val109Ala	c.326T>C
UGT1A1	2	234681059	rs34993780	p.Tyr487Asp	c.1459T>G
UGT1A1	2	234676582	rs755218546	p.Gly363Ser	c.1087G>A
UGT1A1	2	234669558	rs72551343	p.Arg209Trp	c.625C>T
UGT1A1	2	234669768	rs397978903	p.Asn279Tyr	c.835A>T
UGT1A1	2	234675738	rs62625011	p.Gly309Glu	c.926G>A
UGT1A1	2	234675807	rs72551348	p.Gln332Arg	c.995A>G
UGT1A1	2	234676504	rs139607673	p.Arg337Trp	c.1009C>T
UGT1A1	2	234676568	rs72551351	p.Gln358Arg	c.1073A>G
UGT1A1	2	234680907	rs1212085876	intronic	c.1308-1G>A
UGT1A1	2	234678176	rs764863268	intronic	c.1305-1G>A
UGT1A1	2	234678176	rs764863268	intronic	c.1305-1G>C
UGT1A1	2	234675679	rs1285708223	intronic	c.868-1G>A
UGT1A1	2	234669798	rs587776764	intronic	c.864+1G>C
UGT1A1	2	234638641	rs776522612	intronic	c.867+2T>A
UGT1A1	2	234676584	rs779261748	intronic	c.1087+2T>G
UGT1A1	2	234669799	rs772088902	intronic	c.864+2T>C
UGT1A1	2	234581437	rs754609024	intronic	c.855+2T>G
UGT1A1	2	234527210	rs749931479	intronic	c.855+2T>C
UGT1A1	2	234622511	rs752542051	intronic	c.867+7A>G
UGT1A1	2	234622508	rs765022217	intronic	c.867+4T>G
UGT1A1	2	234676589	rs752420110	intronic	c.1087+7T>C
UGT1A1	2	234669802	rs777807265	intronic	c.864+5G>T
UGT1A1	2	234638646	rs569428615	intronic	c.867+7A>T
UGT1A1	2	234677089	rs778641747	intronic	c.1307+4A>G
UGT1A1	2	234638646	rs569428615	intronic	c.867+7A>G
UGT1A1	2	234581443	rs763730748	intronic	c.855+8A>G
UGT1A1	2	234581439	rs752408465	intronic	c.855+4_855+5insCG
UGT1A1	2	234638644	rs559233241	intronic	c.867+5G>A
UGT1A1	2	234581441	rs760517039	intronic	c.855+6_855+7insGCC
UGT1A1	2	234581440	rs778738161	intronic	c.855+5G>A
UGT1A1	2	234675818	rs375883067	intronic	c.999+7A>C
UGT1A1	2	234675818	rs375883067	intronic	c.999+7A>G
UGT1A1	2	234669797	rs748685520	p.Gln288Gln	c.864G>A
UGT1A1	2	234622506	rs764212086	intronic	c.867+2_867+3insACAGA
UGT1A1	2	234677092	rs752429515	intronic	c.1307+7A>G
UGT1A1	2	234677087	rs779826535	intronic	c.1307+8_1307+11delAGAA
UGT1A1	2	234675673	rs753107729	intronic	c.868-7G>A
UGT1A1	2	234676860	rs1298347226	intronic	c.1088-6T>C
UGT1A1	2	234676497	rs752058783	p.Val334Val	c.1002C>G
UGT1A1	2	234676586	rs1471267179	intronic	c.1087+4T>C
UGT1A1	2	234669800	rs1374366123	intronic	c.864+3G>A
UGT1A1	2	234527213		intronic	c.855+5G>A
UGT1A1	2	234622512	rs1467749451	intronic	c.867+8T>C
UGT1A1	2	234621848	rs181492639	p.Glu71Ter	c.211G>T
UGT1A1	2	234581306	rs66915469	p.Tyr242Ter	c.726T>G
UGT1A1	2	234527113	rs771007460	p.Arg254Ter	c.760C>T
UGT1A1	2	234581340	rs772500318	p.Arg254Ter	c.760C>T
UGT1A1	2	234622368	rs549143838	p.Ser244Ter	c.731C>G
UGT1A1	2	234676519	rs72551349	p.Arg342Ter	c.1024C>T
UGT1A1	2	234526481	rs372427845	p.Ser43Ter	c.128C>A
UGT1A1	2	234676567	rs72551350	p.Gln358Ter	c.1072C>T
UGT1A1	2	234638544	rs756538337	p.Arg258Ter	c.772C>T
UGT1A1	2	234622409	rs145951104	p.Arg258Ter	c.772C>T
UGT1A1	2	234637804	rs771653845	p.Trp11Ter	c.32G>A
UGT1A1	2	234681170	rs770564267	p.Arg524Ter	c.1570C>T
UGT1A1	2	234526596	rs754587908	p.Tyr81Ter	c.243C>A
UGT1A1	2	234526897	rs1003369239	p.Gln182Ter	c.544C>T
UGT1A1	2	234676504	rs139607673	p.Tyr338Ter	c.1010_1013dupGGTA
UGT1A1	2	234669780	rs1160983011	p.Gln283Ter	c.847C>T
UGT1A1	2	234637805	rs781569400	p.Trp11Ter	c.33G>A
UGT1A1	2	234621668	rs759060249	p.Gln11Ter	c.31C>T
UGT1A1	2	234526477	rs775618915	p.Gln42Ter	c.124C>T
UGT1A1	2	234526647	rs776735190	p.Trp98Ter	c.294G>A
UGT1A1	2	234621889	rs1157083757	p.Trp84Ter	c.252G>A
UGT1A1	2	234526744	rs755504518	p.Arg131Ter	c.391C>T
UGT1A1	2	234526709	rs776641742	p.Leu119Ter	c.356T>A
UGT1A1	2	234675792	rs372326047	p.Leu327Ter	c.980T>A
UGT1A1	2	234676526	rs144978321	p.Ser344Ter	c.1031C>A
UGT1A1	2	234669695	rs1344319848	p.Trp254Ter	c.762G>A
UGT1A1	2	234527184	rs147191221	p.Cys277Ter	c.831C>A
UGT1A1	2	234669666	rs1412278754	p.Gln245Ter	c.733C>T
UGT1A1	2	234581102	rs1470145388	p.Cys174Ter	c.522C>A
UGT1A1	2	234581108	rs772087611	p.Tyr176Ter	c.528T>A
UGT1A1	2	234580919	rs760653064	p.Tyr113Ter	c.339C>A
UGT1A1	2	234580906	rs775577335	p.Leu109Ter	c.326T>A
UGT1A1	2	234638024	rs1327617845	p.Trp84Ter	c.252G>A
UGT1A1	2	234527105	rs1269683639	p.Trp251Ter	c.752G>A
UGT1A1	2	234527106	rs112944855	p.Trp251Ter	c.753G>A
UGT1A1	2	234638028	rs775388472	p.Gln86Ter	c.256C>T
UGT1A1	2	234580803	rs768968619	p.Thr76_Tyr77insTer	c.225_230dupGACTTA
UGT1A1	2	234622285	rs772966162	p.Tyr216Ter	c.648C>A
UGT1A1	2	234580887	rs772152340	p.Arg103Ter	c.307C>T
UGT1A1	2	234638637	rs770784414	p.Gln289Ter	c.865C>T
UGT1A1	2	234637788	rs757429126	p.Gln6Ter	c.16C>T
UGT1A1	2	234637815	rs770212311	p.Gly15Ter	c.43G>T
UGT1A1	2	234638567	rs772406095	p.Tyr265Ter	c.795C>A
UGT1A1	2	234580693	rs767288021	p.Trp38Ter	c.113G>A
UGT1A1	2	234669053	rs749423657	p.Trp40Ter	c.120G>A
UGT1A1	2	234622134	rs758374226	p.Ser166Ter	c.497C>A
UGT1A1	2	234526545	rs775534837	p.Trp64Ter	c.192G>A
UGT1A1	2	234526765	rs369740303	p.Lys138Ter	c.412A>T
UGT1A1	2	234526761	rs1377457095	p.Tyr136Ter	c.408C>G
UGT1A1	2	234526763	rs375263871	p.Leu137Ter	c.410T>G
UGT1A1	2	234621760	rs1276880544	p.Trp41Ter	c.123G>A
UGT1A1	2	234526584	rs760677046	p.Tyr77Ter	c.231C>G
UGT1A1	2	234526586	rs200115254	p.Ser78Ter	c.233C>G
UGT1A1	2	234669155	rs72551340	p.Tyr74Ter	c.222C>A
UGT1A1	2	234526660	rs763801706	p.Arg103Ter	c.307C>T
UGT1A1	2	234621929	rs764319623	p.Gln98Ter	c.292C>T
UGT1A1	2	234678206	rs542225006	p.Ter445Ter	c.1334G>A
UGT1A1	2	234669074	rs34526305	p.Ile47Ile	c.141C>T
UGT1A1	2	234669473	rs148755655	p.Glu180Glu	c.540A>G
UGT1A1	2	234681031	rs28900406	p.Pro477Pro	c.1431C>T
UGT1A1	2	234676903	rs139698110	p.Gly375Gly	c.1125T>C
UGT1A1	2	234669122	rs191471887	p.Asp63Asp	c.189C>T
UGT1A1	2	234669410	rs199766420	p.Ile159Ile	c.477C>T
UGT1A1	2	234669092	rs149071335	p.Arg53Arg	c.159G>A
UGT1A1	2	234676942	rs763217521	p.Pro388Pro	c.1164C>T
UGT1A1	2	234637803	rs3821242	p.Trp11Arg	c.31T>C
UGT1A1	2	234637912	rs6431625	p.Val47Ala	c.140T>C
UGT1A1	2	234526871	rs1042597	p.Ala173Gly	c.518C>G
UGT1A1	2	234622110	rs12475068	p.Ala158Gly	c.473C>G
UGT1A1	2	234621787	rs3755322	p.Asp50Glu	c.150C>G
UGT1A1	2	234621825	rs3755321	p.Leu63Pro	c.188T>C
UGT1A1	2	234621780	rs3755323	p.Leu48Ser	c.143T>C
UGT1A1	2	234622061	rs3755320	p.His142Asn	c.424C>A
UGT1A1	2	234622310	rs17862867	p.His225Tyr	c.673C>T
UGT1A1	2	234622379	rs2012736	p.Leu248Ile	c.742C>A
UGT1A1	2	234622382	rs17862868	p.Val249Leu	c.745G>C
UGT1A1	2	234622412	rs3892170	p.Gly259Arg	c.775G>C
UGT1A1	2	234638580	rs45449995	p.Met270Val	c.808A>G
UGT1A1	2	234527183	rs17863762	p.Cys277Tyr	c.830G>A
UGT1A1	2	234580678	rs72551330	p.Met33Thr	c.98T>C
UGT1A1	2	234637905	rs45625338	p.Arg45Trp	c.133C>T
UGT1A1	2	234526784	rs17862841	p.Ala144Val	c.431C>T
UGT1A1	2	234638114	rs28898619	p.Met114Ile	c.342G>A
UGT1A1	2	234637789	rs28898617	p.Gln6Arg	c.17A>G
UGT1A1	2	234638203	rs13406898	p.Thr144Ile	c.431C>T
UGT1A1	2	234638245	rs61764030	p.Ala158Val	c.473C>T
UGT1A1	2	234637917	rs45595237	p.Arg49Trp	c.145C>T
UGT1A1	2	234638005	rs28898618	p.Thr78Ile	c.233C>T
UGT1A1	2	234621764	rs45441297	p.Ser43Gly	c.127A>G
UGT1A1	2	234621782	rs41270755	p.Arg49Trp	c.145C>T
UGT1A1	2	234581080	rs151238339	p.Val167Ala	c.500T>C
UGT1A1	2	234580588	rs145084767	p.Cys3Tyr	c.8G>A
UGT1A1	2	234638599	rs371021401	p.Ile276Thr	c.827T>C
UGT1A1	2	234526510	rs45504099	p.His53Asn	c.157C>A
UGT1A1	2	234621645	rs150697955	p.Thr3Arg	c.8C>G
UGT1A1	2	234638508	rs146461519	p.Val246Leu	c.736G>T
UGT1A1	2	234638394	rs140766748	p.Met208Leu	c.622A>C
UGT1A1	2	234638277	rs141036072	p.Thr169Ala	c.505A>G
UGT1A1	2	234526369	rs773705727	p.Trp6Gly	c.16T>G
UGT1A1	2	234526519	rs200702683	p.Val56Ile	c.166G>A
UGT1A1	2	234638547	rs149324549	p.Gly259Arg	c.775G>C
UGT1A1	2	234638451	rs61740163	p.Phe227Val	c.679T>G
UGT1A1	2	234638295	rs61764031	p.Asn175Tyr	c.523A>T
UGT1A1	2	234621839	rs143484996	p.Tyr68His	c.202T>C
UGT1A1	2	234621810	rs577803036	p.Val58Ala	c.173T>C
UGT1A1	2	234622289	rs149715967	p.Leu218Val	c.652C>G
UGT1A1	2	234621777	rs550853040	p.Ala47Val	c.140C>T
UGT1A1	2	234580705	rs756014976	p.Arg42Lys	c.125G>A
UGT1A1	2	234638232	rs138085546	p.Val154Ile	c.460G>A
UGT1A1	2	234526778	rs745712581	p.Phe142Ser	c.425T>C
UGT1A1	2	234638422	rs139850391	p.Pro217Leu	c.650C>T
UGT1A1	2	234669681	rs57307513	p.Ser250Pro	c.748T>C
UGT1A1	2	234677012	rs36076514	p.Val412Leu	c.1234G>T
UGT1A1	2	234581200	rs201242485	p.Val207Glu	c.620T>A
UGT1A1	2	234581360	rs200148096	p.Asp260Glu	c.780C>G
UGT1A1	2	234526807	rs1042593	p.Ala152Thr	c.454G>A
UGT1A1	2	234638110	rs146268573	p.Ser113Thr	c.338G>C
UGT1A1	2	234638044	rs540607993	p.Arg91His	c.272G>A
UGT1A1	2	234621909	rs146477190	p.Arg91His	c.272G>A
UGT1A1	2	234637944	rs140541315	p.Ala58Thr	c.172G>A
UGT1A1	2	234581127	rs145038612	p.Cys183Gly	c.547T>G
UGT1A1	2	234526975	rs150087070	p.Arg208Trp	c.622C>T
UGT1A1	2	234676866	rs752968297	p.Gly363Val	c.1088G>T
UGT1A1	2	234622316	rs200994534	p.Val227Phe	c.679G>T
UGT1A1	2	234676535	rs201372184	p.Ala347Val	c.1040C>T
UGT1A1	2	234581395	rs570410415	p.Ile272Thr	c.815T>C
UGT1A1	2	234581083	rs145923738	p.Phe168Ser	c.503T>C
UGT1A1	2	234637991	rs200579886	p.Asn73Lys	c.219C>A
UGT1A1	2	234581106	rs141811044	p.Tyr176His	c.526T>C
UGT1A1	2	234581202	rs111861762	p.Arg208Trp	c.622C>T
UGT1A1	2	234581299	rs561534651	p.Thr240Met	c.719C>T
UGT1A1	2	234622464	rs147668404	p.Ile276Thr	c.827T>C
UGT1A1	2	234638269	rs142986334	p.Ser166Leu	c.497C>T
UGT1A1	2	234580755	rs199829358	p.Met59Leu	c.175A>C
UGT1A1	2	234581353	rs765305963	p.Val258Ala	c.773T>C
UGT1A1	2	234621669	rs576086579	p.Gln11Arg	c.32A>G
UGT1A1	2	234527174	rs183687751	p.Gly274Ala	c.821G>C
UGT1A1	2	234621770	rs746932812	p.Arg45Trp	c.133C>T
UGT1A1	2	234526694	rs145367192	p.Asn114Ser	c.341A>G
UGT1A1	2	234638622	rs201645683	p.Arg284Gly	c.850A>G
UGT1A1	2	234580749	rs767768121	p.Val57Ile	c.169G>A
UGT1A1	2	234680925	rs202172337	p.Met442Thr	c.1325T>C
UGT1A1	2	234581382	rs376424202	p.Asn268Asp	c.802A>G
UGT1A1	2	234637825	rs200023814	p.Leu18Pro	c.53T>C
UGT1A1	2	234621995	rs774630378	p.Met120Val	c.358A>G
ABCC6	16	16244031	rs1219230462	p.Leu1487_Lys1490del	c.4459_4470delCTGGCCCAGAAG
ABCC6	16	16244049	rs566671584	p.Gln1485Ter	c.4453C>T
ABCC6	16	16244100	rs1368211583		c.4404-2A>C
ABCC6	16	16244434	rs1006994885		c.4403+1G>T
ABCC6	16	16244525	rs74315109	p.Thr1436CysfsTer26	c.4306_4312delACGGAGC
ABCC6	16	16244583	rs1448934731	p.Lys1419ArgfsTer45	c.4254delG
ABCC6	16	16244616	rs1287240953	p.Gln1408Ter	c.4222C>T
ABCC6	16	16244620	rs149510465	p.Lys1407GlufsTer121	c.4217dupA
ABCC6	16	16244629	rs63750700	p.Ser1403Arg	c.4209C>A
ABCC6	16	16248495	rs63751241	p.Glu1400Lys	c.4198G>A
ABCC6	16	16248501	rs66913554	p.Arg1398Ter	c.4192C>T
ABCC6	16	16248510	rs67791546	p.Lys1394AsnfsTer9	c.4182delG
ABCC6	16	16248521	rs747334212	p.Leu1391ProfsTer11	c.4159_4171dupCTGCCCGGCCAGC
ABCC6	16	16248567	rs748103958	p.Glu1376Ter	c.4126G>T
ABCC6	16	16248588	rs60285147	p.Asp1368GlufsTer35	c.4104delC
ABCC6	16	16248612	rs58695352	p.Asp1361Asn	c.4081G>A
ABCC6	16	16248623	rs201275608	p.Arg1357Gln	c.4070G>A
ABCC6	16	16248624	rs63750428	p.Arg1357Trp	c.4069C>T
ABCC6	16	16248746	rs63750608	p.Ile1342Thr	c.4025T>C
ABCC6	16	16248755	rs63750622	p.Arg1339His	c.4016G>A
ABCC6	16	16248756	rs28939702	p.Arg1339Cys	c.4015C>T
ABCC6	16	16248758	rs1436397251	p.Leu1338AlafsTer59	c.4011_4012delAC
ABCC6	16	16248783	rs58902671	p.Ile1330HisfsTer68	c.3987dupC
ABCC6	16	16248818	rs780887287	p.Ala1318Gly	c.3953C>G
ABCC6	16	16248830	rs63751086	p.Arg1314Gln	c.3941G>A
ABCC6	16	16248831	rs63750759	p.Arg1314Trp	c.3940C>T
ABCC6	16	16248864	rs63750410	p.Ala1303Pro	c.3907G>C
ABCC6	16	16248867	rs63749856	p.Gly1302Arg	c.3904G>A
ABCC6	16	16248869	rs63750494	p.Thr1301Ile	c.3902C>T
ABCC6	16	16248876	rs63750446	p.Gly1299Ser	c.3895G>A
ABCC6	16	16248879	rs63751325	p.Val1298Ile	c.3892G>A
ABCC6	16	16248884	rs374086268	p.Gly1296Asp	c.3887G>A
ABCC6	16	16251519	rs72664235	p.Lys1294del	c.3880_3882delAAG
ABCC6	16	16251579	rs72653749	p.Arg1275Ter	c.3823C>T
ABCC6	16	16251580	rs769820268	p.Tyr1274Ter	c.3822C>A
ABCC6	16	16251612	rs1311228469	p.Gln1264Ter	c.3790C>T
ABCC6	16	16251626	rs72664233	p.Trp1259GlyfsTer14	c.3775delT
ABCC6	16	16251627	rs72664221	p.Trp1259LeufsTer19	c.3774dupC
ABCC6	16	16251635	rs1364050032	p.Pro1247_Ala1255dup	c.3740_3766dupCCTGGAGGCTGCCCACATGTGCAGCTC
ABCC6	16	16251644	rs764868012	p.Cys1253ValfsTer20	c.3757delT
ABCC6	16	16251651	rs148326870	p.Pro1251Ser	c.3751C>T
ABCC6	16	16251667	rs63750273		c.3736-1G>A
ABCC6	16	16253339	rs281865557	p.Glu1245Asp	c.3735G>T
ABCC6	16	16253352	rs72653748	p.Trp1241Ter	c.3722G>A
ABCC6	16	16253365	rs72653746	p.Gln1237Ter	c.3709C>T
ABCC6	16	16253370	rs138700741	p.Arg1235Gln	c.3704G>A
ABCC6	16	16253371	rs63750402	p.Arg1235Trp	c.3703C>T
ABCC6	16	16253381	rs779018991	p.Ser1232ValfsTer46	c.3692dupT
ABCC6	16	16253412	rs63751001	p.Arg1221His	c.3662G>A
ABCC6	16	16253413	rs63751215	p.Arg1221Cys	c.3661C>T
ABCC6	16	16255312	rs375983928	p.Ser1205CysfsTer72	c.3614_3615delCT
ABCC6	16	16255410	rs758506257	p.Ala1173ProfsTer20	c.3517delG
ABCC6	16	16255418	rs1249984461	p.Trp1170Ter	c.3510G>A
ABCC6	16	16255422	rs72664210		c.3507-1G>A
ABCC6	16	16255423	rs1423674851		c.3507-2A>C
ABCC6	16	16255423	rs1423674851		c.3507-2A>G
ABCC6	16	16256844	rs747496143		c.3506+2_3506+5delTAGG
ABCC6	16	16256849	rs1359856569		c.3506+1G>A
ABCC6	16	16256865	rs63750457	p.Arg1164Gln	c.3491G>A
ABCC6	16	16256866	rs72653744	p.Arg1164Ter	c.3490C>T
ABCC6	16	16256867	rs202000035	p.Pro1163ArgfsTer30	c.3488delC
ABCC6	16	16256898	rs553479685	p.Arg1153His	c.3458G>A
ABCC6	16	16256920	rs769105086	p.Phe1146LeufsTer9	c.3435delC
ABCC6	16	16256922		p.Gln1143_Ala1144del	c.3428_3433delAGGCCC
ABCC6	16	16256935	rs72653706	p.Arg1141Ter	c.3421C>T
ABCC6	16	16256941	rs63750146	p.Ala1139Thr	c.3415G>A
ABCC6	16	16256943	rs60791294	p.Arg1138Gln	c.3413G>A
ABCC6	16	16256944	rs28939701	p.Arg1138Trp	c.3412C>T
ABCC6	16	16256959	rs63749807	p.Gly1133Cys	c.3397G>T
ABCC6	16	16256967	rs63750459	p.Thr1130Met	c.3389C>T
ABCC6	16	16256994	rs63750987	p.Ser1121Leu	c.3362C>T
ABCC6	16	16257009	rs1321818108	p.Leu1115_Glu1116insVal	c.3344_3346dupTGG
ABCC6	16	16257015	rs63750427	p.Arg1114Pro	c.3341G>C
ABCC6	16	16257015	rs63750427	p.Arg1114His	c.3341G>A
ABCC6	16	16257016	rs63749794	p.Arg1114Cys	c.3340C>T
ABCC6	16	16257030	rs758648156	p.Ser1109Ter	c.3326C>A
ABCC6	16	16257050	rs200386727		c.3307-1G>C
ABCC6	16	16257051	rs1167145612		c.3307-2A>C
ABCC6	16	16259579	rs60975032	p.Tyr1069Ter	c.3207C>A
ABCC6	16	16259598	rs72657695	p.Leu1063Arg	c.3188T>G
ABCC6	16	16259602	rs377653646	p.Lys1062Ter	c.3184A>T
ABCC6	16	16259640	rs769437554	p.Phe1048del	c.3143_3145delTCT
ABCC6	16	16259677	rs754074990	p.Phe1036del	c.3106_3108delTTT
ABCC6	16	16259698	rs72653705	p.Arg1030Ter	c.3088C>T
ABCC6	16	16259709	rs1254748860	p.Trp1026Ter	c.3077G>A
ABCC6	16	16263506	rs777193567	p.Gln998Ter	c.2992C>T
ABCC6	16	16263518		p.Val977SerfsTer18	c.2928_2979delAGTAGGTGGGCAGCAGACGCAGGCAGCCCTGCGTGGCGGGATCTTCGGGCTC
ABCC6	16	16263524	rs72657692	p.Gly992Arg	c.2974G>A
ABCC6	16	16263613	rs759512269	p.Ser961del	c.2882_2884delCCT
ABCC6	16	16263631	rs767359198	p.Phe954_Leu955del	c.2861_2866delTCCTCT
ABCC6	16	16263650	rs72657689	p.Ala950Thr	c.2848G>A
ABCC6	16	16263651	rs553008971	p.Tyr949Ter	c.2847C>G
ABCC6	16	16263683	rs752477633	p.His935ProfsTer99	c.2804_2814delACCTGGCCTAC
ABCC6	16	16267139	rs769762685		c.2787+2T>A
ABCC6	16	16267140	rs72664209		c.2787+1G>T
ABCC6	16	16267140	rs72664209	p.Arg929Ter	c.2784_2787delCAGG
ABCC6	16	16267147	rs61731973	p.Tyr927Ter	c.2781C>A
ABCC6	16	16267174		p.Trp918Ter	c.2754G>A
ABCC6	16	16267250	rs1481200467	p.Ser893Ter	c.2678C>A
ABCC6	16	16267262	rs1337387527		c.2667-1G>A
ABCC6	16	16269766	rs1254815036		c.2666+2T>G
ABCC6	16	16269844	rs1131691865		c.2591-1G>T
ABCC6	16	16271311	rs1328509959	p.Glu862del	c.2585_2587delAAG
ABCC6	16	16271356	rs67867306	p.Met848CysfsTer83	c.2542delA
ABCC6	16	16271387	rs756709738	p.Gln838Ter	c.2512C>T
ABCC6	16	16271388	rs72650702	p.Tyr837Ter	c.2511C>A
ABCC6	16	16271441	rs72653797	p.Ala820Pro	c.2458G>C
ABCC6	16	16271467	rs72653796	p.Thr811Met	c.2432C>T
ABCC6	16	16271467	rs72653796	p.Thr811Arg	c.2432C>G
ABCC6	16	16271479	rs72653794	p.Arg807Gln	c.2420G>A
ABCC6	16	16271480	rs72653793	p.Arg807Trp	c.2419C>T
ABCC6	16	16272680	rs768570780	p.Gly797Glu	c.2390G>A
ABCC6	16	16272711	rs72653792	p.Val787Ile	c.2359G>A
ABCC6	16	16272746	rs1249927722	p.Leu775CysfsTer36	c.2323delC
ABCC6	16	16272776	rs67561842	p.Arg765Gln	c.2294G>A
ABCC6	16	16272777	rs776513864	p.Arg765Trp	c.2293C>T
ABCC6	16	16272791	rs769405586	p.Arg760Gln	c.2279G>A
ABCC6	16	16272792	rs72653788	p.Arg760Trp	c.2278C>T
ABCC6	16	16272807	rs72653787	p.Gly755Arg	c.2263G>A
ABCC6	16	16272818	rs72653786	p.Met751Lys	c.2252T>A
ABCC6	16	16276268	rs781190396		c.2247+1G>A
ABCC6	16	16276273	rs748503108	p.Glu748GlyfsTer31	c.2242dupG
ABCC6	16	16276279	rs769753486	p.Ile746ArgfsTer36	c.2236_2237insGGTGCAGAGT
ABCC6	16	16276363	rs1357894483	p.Asp718Gly	c.2153A>G
ABCC6	16	16276364	rs1427385368	p.Leu717dup	c.2149_2151dupCTG
ABCC6	16	16276373	rs1158709263	p.Gln715ArgfsTer12	c.2142delG
ABCC6	16	16276419	rs72653784	p.Glu699Asp	c.2097G>T
ABCC6	16	16276423	rs72653783	p.Gln698ArgfsTer11	c.2092delC
ABCC6	16	16276431	rs199913903	p.Tyr695Ter	c.2085C>A
ABCC6	16	16276681	rs1279963123	p.Glu684Ter	c.2050G>T
ABCC6	16	16276683	rs758980634	p.Val683TrpfsTer5	c.2047delG
ABCC6	16	16276713	rs67470842	p.Leu673Pro	c.2018T>C
ABCC6	16	16276731	rs775321637	p.Ala667GlnfsTer21	c.1999delG
ABCC6	16	16276731	rs775321637	p.Ala667GlyfsTer74	c.1999dupG
ABCC6	16	16276744	rs72653780	p.Gly663Cys	c.1987G>T
ABCC6	16	16276746	rs760707891	p.Ala660SerfsTer26	c.1978_1984delGCTGTTG
ABCC6	16	16276765	rs74315158	p.Ile649AlafsTer32	c.1944_1965delAATAAACCTCACGGTGCCCCAG
ABCC6	16	16276768	rs369410139	p.Gln655Ter	c.1963C>T
ABCC6	16	16276788	rs1438851867		c.1944-1G>A
ABCC6	16	16278815	rs748132404		c.1943+1G>C
ABCC6	16	16281023	rs749334729	p.Glu609Ter	c.1825G>T
ABCC6	16	16281042	rs781369291	p.Thr603SerfsTer11	c.1799_1805dupGTCTGGT
ABCC6	16	16281049	rs761433545	p.Arg600His	c.1799G>A
ABCC6	16	16281050	rs72653777	p.Arg600Cys	c.1798C>T
ABCC6	16	16281056	rs1253009659	p.Phe598LeufsTer18	c.1791delC
ABCC6	16	16281061	rs1180478434	p.Val596CysfsTer20	c.1786delG
ABCC6	16	16281067	rs72653776	p.Ala594Val	c.1781C>T
ABCC6	16	16282687	rs768037422		c.1779+1G>C
ABCC6	16	16282690	rs1249307722	p.Gln593Ter	c.1777C>T
ABCC6	16	16282698	rs537233133	p.Ser590Phe	c.1769C>T
ABCC6	16	16282750	rs1410903760	p.Thr572SerfsTer27	c.1715_1716delCA
ABCC6	16	16282764	rs66864704	p.Phe568Ser	c.1703T>C
ABCC6	16	16282828	rs56877937	p.Ala547Thr	c.1639G>A
ABCC6	16	16284056	rs199634124	p.Phe532CysfsTer66	c.1595_1599delTCTCT
ABCC6	16	16284095	rs1218099786	p.Glu521Ter	c.1561G>T
ABCC6	16	16284103	rs72653772	p.Arg518Gln	c.1553G>A
ABCC6	16	16284104	rs72650700	p.Arg518Ter	c.1552C>T
ABCC6	16	16284116	rs59157279	p.Val514Ile	c.1540G>A
ABCC6	16	16284130	rs779408186	p.Ala509Gly	c.1526C>G
ABCC6	16	16284137	rs368017088	p.Glu507Ter	c.1519G>T
ABCC6	16	16284172	rs72653769	p.Leu495His	c.1484T>A
ABCC6	16	16284196	rs72653768	p.Arg487Gln	c.1460G>A
ABCC6	16	16286694	rs151187637	p.His475Leu	c.1424A>T
ABCC6	16	16286780	rs1335726550		c.1339-1G>C
ABCC6	16	16291877	rs866852986		c.1338+1G>T
ABCC6	16	16291882	rs1294485971	p.Trp445Ter	c.1334G>A
ABCC6	16	16291952	rs770532500	p.Glu422ArgfsTer39	c.1263delC
ABCC6	16	16291960	rs772434460	p.Arg419Gln	c.1256G>A
ABCC6	16	16291961	rs775853778	p.Arg419Trp	c.1255C>T
ABCC6	16	16295863	rs72653762	p.Arg391Gly	c.1171A>G
ABCC6	16	16295876	rs1336214807	p.Thr386MetfsTer35	c.1157delC
ABCC6	16	16295902	rs72650699	p.Gln378Ter	c.1132C>T
ABCC6	16	16295926	rs72653760	p.Asn370Asp	c.1108A>G
ABCC6	16	16295934	rs1400346389	p.Phe366SerfsTer13	c.1096_1099delTTTG
ABCC6	16	16295943	rs72653759	p.Thr364Arg	c.1091C>G
ABCC6	16	16295970	rs72653758	p.Leu355Arg	c.1064T>G
ABCC6	16	16295974	rs372132926	p.Val354Met	c.1060G>A
ABCC6	16	16295976	rs369074083	p.Ala353Val	c.1058C>T
ABCC6	16	16296036	rs1193846383		c.999-1G>A
ABCC6	16	16297263	rs72664204		c.998+2_998+3delTG
ABCC6	16	16297266	rs1005584828		c.998+1G>A
ABCC6	16	16297277	rs1345727670	p.Lys330SerfsTer26	c.987delC
ABCC6	16	16297314	rs78678589	p.Ser317Arg	c.951C>A
ABCC6	16	16297352	rs746625905	p.Gln305Ter	c.913C>T
ABCC6	16	16297424	rs879274205	p.Lys281Glu	c.841A>G
ABCC6	16	16302584	rs775749515		c.794+1G>A
ABCC6	16	16302585	rs760880587	p.Arg265GlyfsTer6	c.793delA
ABCC6	16	16302637	rs72653756	p.Leu248Phe	c.742C>T
ABCC6	16	16302637	rs72653756	p.Glu247del	c.739_741delGAA
ABCC6	16	16302644	rs1389789583	p.Ser245del	c.732_734delCTC
ABCC6	16	16302645	rs761731967	p.Ser245Ter	c.734C>A
ABCC6	16	16302669	rs1343632105	p.Trp237SerfsTer22	c.708_709dupCT
ABCC6	16	16306041	rs758932406		c.662+1G>T
ABCC6	16	16306081	rs1326299196	p.Ala208GlyfsTer46	c.622dupG
ABCC6	16	16308180	rs1187315015		c.600+1G>A
ABCC6	16	16308184	rs756622503	p.Gln199HisfsTer3	c.596_597insT
ABCC6	16	16308186	rs1474063386	p.Gln199Ter	c.595C>T
ABCC6	16	16308207	rs1172142986	p.Phe192SerfsTer40	c.573delC
ABCC6	16	16308223	rs1342109356	p.Leu186ArgfsTer46	c.557delT
ABCC6	16	16308234	rs764710238	p.Leu183AlafsTer13	c.546dupG
ABCC6	16	16308285	rs201766106	p.Arg166Cys	c.496C>T
ABCC6	16	16313493	rs369280729	p.Gln131Arg	c.392A>G
ABCC6	16	16313499	rs72653753	p.Gly129Glu	c.386G>A
ABCC6	16	16313512	rs879956688	p.Glu125Ter	c.373G>T
ABCC6	16	16313806	rs1260772144		c.220-2A>G
ABCC6	16	16315444	rs770579364	p.Trp94Ter	c.281G>A
ABCC6	16	16315501	rs758129215	p.Ala75LeufsTer23	c.223delG
ABCC6	16	16315546	rs183648123	p.Arg60Gln	c.179G>A
ABCC6	16	16315600	rs779429021	p.Met42ThrfsTer59	c.124_125insC
ABCC6	16	16315601	rs746531177	p.Met42CysfsTer39	c.123delC
ABCC6	16	16315601	rs746531177	p.Met42HisfsTer59	c.123dupC
ABCC6	16	16315619	rs72664223	p.Val37SerfsTer44	c.105delA
ABCC6	16	16315670	rs776575086	p.Glu18AlafsTer2	c.53_54delAG
ABCC6	16	16315689	rs72657702		c.37-1G>A
ABCC6	16	16317268	rs1412967184	p.Cys8Ter	c.24C>A
SLC22A1	6	160542970	rs754315162	p.Met1?	c.3G>A
SLC22A1	6	160543068	rs776450090	p.Ile35GlyfsTer10	c.102_109delCATCTGTG
SLC22A1	6	160543111	rs1477152702	p.His49ProfsTer7	c.146_150delACTGC
SLC22A1	6	160543147	rs1341056789	p.Arg61ProfsTer32	c.181dupC
SLC22A1	6	160543180	rs1369274021	p.Thr73SerfsTer19	c.216_217delTA
SLC22A1	6	160543214	rs759376914	p.Phe84LeufsTer19	c.252_255delCCTT
SLC22A1	6	160543256	rs772259161	p.Gln97Ter	c.289C>T
SLC22A1	6	160543330	rs200865946	p.Cys121Ter	c.363C>A
SLC22A1	6	160543331	rs1400802949	p.Gln122Ter	c.364C>T
SLC22A1	6	160543341	rs769565823	p.Trp125Ter	c.374G>A
SLC22A1	6	160543342	rs1290210327	p.Trp125Ter	c.375G>A
SLC22A1	6	160543373	rs765176277	p.Thr136MetfsTer20	c.407delC
SLC22A1	6	160551134	rs759733993		c.412-2A>G
SLC22A1	6	160551135	rs765396308		c.412-1G>T
SLC22A1	6	160551159	rs1245668483	p.Trp146GlyfsTer10	c.436delT
SLC22A1	6	160551178	rs1415929945	p.Gln152Ter	c.454C>T
SLC22A1	6	160551201	rs763052165	p.Leu160del	c.480_482delGTT
SLC22A1	6	160551228	rs763594416	p.Phe169LeufsTer55	c.507delT
SLC22A1	6	160551230	rs751047188		c.509_515+5delCAGACAGGTATG
SLC22A1	6	160551240	rs1156659487		c.515+1G>T
SLC22A1	6	160551240	rs1156659487		c.515+1G>C
SLC22A1	6	160553263	rs769562245		c.516-1G>T
SLC22A1	6	160553272	rs144273196	p.Lys176Ter	c.525dupT
SLC22A1	6	160553325	rs370895155	p.Met1?	c.1A>G
SLC22A1	6	160553399	rs1282222356	p.Trp217Ter	c.651G>A
SLC22A1	6	160554986	rs371854539		c.671-2delA
SLC22A1	6	160554987	rs1202234972		c.671-2A>C
SLC22A1	6	160555015	rs1377834408	p.Arg233Ter	c.697A>T
SLC22A1	6	160555038	rs567722552	p.Tyr240Ter	c.720C>A
SLC22A1	6	160555039	rs780737592	p.Gln241ArgfsTer59	c.722delA
SLC22A1	6	160555107	rs1204306128	p.Trp263Ter	c.789G>A
SLC22A1	6	160555152	rs765282373	p.Tyr278Ter	c.834C>G
SLC22A1	6	160555155	rs201662351	p.Tyr279Ter	c.837C>G
SLC22A1	6	160555157	rs377669979	p.Trp280Ter	c.839G>A
SLC22A1	6	160555158	rs751852957		c.839+2dupT
SLC22A1	6	160555158	rs751852957		c.839+1G>T
SLC22A1	6	160555158	rs751852957		c.839+1G>A
SLC22A1	6	160555159	rs757388620		c.839+2T>C
SLC22A1	6	160555159	rs757388620		c.839+2T>A
SLC22A1	6	160557250	rs781019292		c.840-2A>T
SLC22A1	6	160557286	rs778990930	p.Arg294LysfsTer4	c.880dupA
SLC22A1	6	160557298	rs1213367085	p.Thr296MetfsTer4	c.887delC
SLC22A1	6	160557320	rs181861830	p.His304ThrfsTer14	c.910delC
SLC22A1	6	160557367	rs148035306		c.954+1G>C
SLC22A1	6	160557639	rs1317621385	p.Pro341dup	c.1022_1024dupCGC
SLC22A1	6	160557646	rs34205214	p.Arg344_Leu350del	c.1031_1051delGGAAGCGCACCTTCATCCTGA
SLC22A1	6	160560696	rs763656391	p.Val359AlafsTer39	c.1076_1077delTG
SLC22A1	6	160560783	rs1028510253	p.Ala390GlyfsTer9	c.1163dupC
SLC22A1	6	160560827	rs369672295	p.Arg402ProfsTer19	c.1205delG
SLC22A1	6	160560849	rs1212347725	p.Asn410PhefsTer16	c.1228_1229delAA
SLC22A1	6	160560880	rs35167514	p.Met420del	c.1260_1262delGAT
SLC22A1	6	160560882	rs1339749834	p.Met420IlefsTer6	c.1260_1261delGA
SLC22A1	6	160560894	rs1186925771	p.Ser424Ter	c.1271C>G
SLC22A1	6	160564573	rs777976920	p.Leu427CysfsTer4	c.1279delC
SLC22A1	6	160564582	rs1379400132	p.Trp429Ter	c.1286G>A
SLC22A1	6	160564598	rs747163555	p.Met435ValfsTer20	c.1303_1304delAT
SLC22A1	6	160564611	rs144231190	p.Arg439Ter	c.1315C>T
SLC22A1	6	160564682	rs755349045		c.1385+1G>C
SLC22A1	6	160564682	rs755349045		c.1385+1G>A
SLC22A1	6	160575829	rs749250141		c.1386-1G>T
SLC22A1	6	160575851	rs1337675540	p.Ser471dup	c.1411_1413dupTCC
SLC22A1	6	160575882	rs1196083959	p.Phe482SerfsTer2	c.1443delC
SLC22A1	6	160577034	rs1226135972	p.Thr510ArgfsTer16	c.1527delG
SLC22A1	6	160577058	rs768534532	p.Val519ArgfsTer26	c.1554_1555delGG
SLC22A1	6	160577092	rs562629011	p.Arg491Ter	c.1471C>T
SLC22A1	6	160579547	rs771232417		c.1599-1G>T
SLC22A1	6	160579559	rs1172317436	p.Glu539GlnfsTer8	c.1611_1614dupCAAA
SLC22A1	6	160579560	rs748864081	p.Gln500Ter	c.1498C>T
SLC22A1	6	160579582	rs533721314	p.Lys545Ter	c.1633A>T
SLC22A1	6	160579583	rs766245985	p.Val546SerfsTer?	c.1636delG
SLC22A1	6	160579612	rs766927825		c.*5_*6delGA
SLCO1A2	12	21422526	rs777152983	p.Lys657Ter	c.1969A>T
SLCO1A2	12	21422534	rs199614868	p.Ile654ArgfsTer12	c.1957_1960dupGATA
SLCO1A2	12	21422544	rs142017745	p.Glu650dup	c.1948_1950dupGAG
SLCO1A2	12	21422580	rs746215590	p.Glu638AlafsTer12	c.1913_1914delAG
SLCO1A2	12	21422591	rs753630199	p.Ser635ArgfsTer15	c.1902_1903delTT
SLCO1A2	12	21422620	rs1276325435	p.Lys625SerfsTer16	c.1874delA
SLCO1A2	12	21422684	rs766330899	p.Leu604Ter	c.1811T>A
SLCO1A2	12	21422688	rs369215769	p.Leu602ArgfsTer29	c.1805_1806delTC
SLCO1A2	12	21427415	rs748973573	p.Ser594Ter	c.1777_1780dupGATT
SLCO1A2	12	21427459	rs3830207	p.Thr580AsnfsTer6	c.1736dupG
SLCO1A2	12	21428225	rs1276890677	p.Ter580GlnextTer?	c.1738T>C
SLCO1A2	12	21428234	rs764101200	p.Phe576LeufsTer?	c.1728delT
SLCO1A2	12	21428238	rs766927298	p.Leu573_Leu574del	c.1719_1724delATTATT
SLCO1A2	12	21428238	rs766927298	p.Leu574dup	c.1722_1724dupATT
SLCO1A2	12	21428302	rs1487428339	p.Arg556GlufsTer15	c.1666delA
SLCO1A2	12	21428318	rs138287536	p.His551ThrfsTer35	c.1650dupA
SLCO1A2	12	21445097	rs747975230		c.1610+1G>A
SLCO1A2	12	21445177	rs751942663	p.Gln511Ter	c.1531C>T
SLCO1A2	12	21445189	rs777190986	p.Cys506PhefsTer36	c.1517_1518delGT
SLCO1A2	12	21445213	rs748771645	p.Val496AlafsTer7	c.1487_1494delTTCTTGGG
SLCO1A2	12	21445230	rs746068777	p.Ser493Ter	c.1478C>A
SLCO1A2	12	21445258	rs1223563284	p.Cys484MetfsTer22	c.1449_1450insA
SLCO1A2	12	21445271	rs1452964168		c.1438-1G>C
SLCO1A2	12	21446930	rs778532014	p.Leu462ValfsTer8	c.1384_1385delCT
SLCO1A2	12	21446962	rs749389235	p.Pro452LeufsTer26	c.1353delT
SLCO1A2	12	21446977	rs1210056542	p.Pro446del	c.1336_1338delCCA
SLCO1A2	12	21447008	rs745457396	p.Ile435CysfsTer2	c.1303_1307delATCTT
SLCO1A2	12	21448530	rs748117966		c.1271+1G>T
SLCO1A2	12	21448530	rs748117966		c.1271+1G>C
SLCO1A2	12	21448539	rs760557358	p.Val415_Ser421del	c.1242_1262delAGTTGTTGGAATAAATACCTC
SLCO1A2	12	21448559	rs1365727875	p.Ser414CysfsTer9	c.1241_1242delCA
SLCO1A2	12	21448590		p.Leu404IlefsTer7	c.1210_1211delTT
SLCO1A2	12	21448626	rs368319612	p.Cys392PhefsTer17	c.1175delG
SLCO1A2	12	21448683	rs768389180	p.Gly374TrpfsTer25	c.1118dupG
SLCO1A2	12	21448710	rs1387892963	p.Pro364LeufsTer8	c.1091delC
SLCO1A2	12	21448728	rs776489569		c.1076-2A>G
SLCO1A2	12	21450337	rs1173676264		c.1075+1G>T
SLCO1A2	12	21450385	rs1324476681	p.Leu343ProfsTer2	c.1026_1027dupCC
SLCO1A2	12	21450435	rs1453069655	p.Ser325_Val326del	c.972_977delAAGTGT
SLCO1A2	12	21450471	rs534910521	p.Cys314Ter	c.942C>A
SLCO1A2	12	21453280	rs750687714		c.900_910+1delAATCACTAAAGG
SLCO1A2	12	21453320	rs1461445706	p.Gln290LysfsTer19	c.868_871delCAAA
SLCO1A2	12	21453333	rs1220058538	p.Glu287Ter	c.859G>T
SLCO1A2	12	21453339	rs761378365	p.Glu285Ter	c.853G>T
SLCO1A2	12	21453386	rs1419986195	p.Thr269HisfsTer7	c.805delA
SLCO1A2	12	21453390	rs763186437	p.Asn268ThrfsTer8	c.801delC
SLCO1A2	12	21453400	rs751924085	p.Phe264SerfsTer12	c.791delT
SLCO1A2	12	21453469	rs754967522	p.Trp241Ter	c.723G>A
SLCO1A2	12	21453502	rs1224003162		c.689-1_689delGA
SLCO1A2	12	21454130	rs746070880	p.Tyr221Ter	c.663T>A
SLCO1A2	12	21454158	rs770726998	p.Leu212Ter	c.635T>A
SLCO1A2	12	21454204	rs41275204		c.590-1G>A
SLCO1A2	12	21457401	rs753412676	p.Ile183LysfsTer17	c.548delT
SLCO1A2	12	21457458	rs756764472	p.Asn165ArgfsTer9	c.488_491dupTAGG
SLCO1A2	12	21457470	rs766171074	p.Tyr160Ter	c.480C>A
SLCO1A2	12	21457476	rs759797766	p.Trp158Ter	c.474G>A
SLCO1A2	12	21457477	rs1179420309	p.Trp158delinsTyrThrArg	c.472_473insACACTA
SLCO1A2	12	21457486	rs1180103765	p.Ser155Ter	c.464C>A
SLCO1A2	12	21457508	rs752085370		c.443-1G>A
SLCO1A2	12	21459814	rs1216749671		c.442_442+1dupGG
SLCO1A2	12	21459861	rs764329847	p.Met1?	c.1A>G
SLCO1A2	12	21459872	rs774635728	p.Asn128ValfsTer12	c.382_385delAACA
SLCO1A2	12	21459912	rs758031550	p.Glu116Ter	c.346G>T
SLCO1A2	12	21467482	rs79044647		c.335+1G>A
SLCO1A2	12	21467484		p.Gln112Ter	c.334C>T
SLCO1A2	12	21467539	rs756832201	p.Cys93Ter	c.279T>A
SLCO1A2	12	21467571	rs773387618	p.Lys82AsnfsTer6	c.246delA
SLCO1A2	12	21467616	rs267603420		c.203-1G>A
SLCO1A2	12	21467617	rs200944946		c.203-2A>G
SLCO1A2	12	21471714	rs777836270		c.202+2T>A
SLCO1A2	12	21471725	rs373967231	p.Phe65LeufsTer8	c.192delC
SLCO1A2	12	21471770	rs541134154	p.Glu48AspfsTer9	c.144_147delGAGA
SLCO1A2	12	21471807	rs767802917	p.Gly37AspfsTer4	c.110delG
SLCO1A2	12	21471835	rs979216737	p.Ala29IlefsTer33	c.81_82delAT
SLCO1A2	12	21471859	rs1158189135		c.61-2A>C
SLCO1A2	12	21472305	rs1320482326		c.54+1G>A
SLCO1A2	12	21472308	rs770643301	p.Lys18ArgfsTer7	c.51delC
SLCO1A2	12	21472317	rs1255172615	p.Val14GlyfsTer46	c.41_42delTG
SLCO1A2	12	21487521	rs748746711		c.60+1G>T
SLCO1A2	12	21487563	rs1328144946	p.Arg7Ter	c.19A>T
SLCO1A2	12	21487581	rs758931217	p.Met1?	c.1A>G

## References

[1] Tian X, Zhang H, Heimbach T, He H, Buchbinder A, Aghoghovbia M, Hourcade-Potelleret F (2018). Clinical pharmacokinetic and pharmacodynamic overview of nilotinib, a selective tyrosine kinase inhibitor. J Clin Pharmacol.

[2] Kumar V, Singh P, Gupta SK, Ali V, Verma M (2022). Transport and metabolism of tyrosine kinase inhibitors associated with chronic myeloid leukemia therapy: a review. Mol Cell Biochem.

[3] Mauro MJ (2006). Defining and managing imatinib resistance. Hematology Am Soc Hematol Educ Program.

[4] (2024). Chronic myeloid leukemia. https://emedicine.medscape.com/article/199425-overview.

[5] (2024). Cancer Stat Facts: Leukemia — chronic myeloid leukemia (CML). https://seer.cancer.gov/statfacts/html/cmyl.html.

[6] Yang Y, Li S, Wang Y, Zhao Y, Li Q (2022). Protein tyrosine kinase inhibitor resistance in malignant tumors: molecular mechanisms and future perspective. Signal Transduct Target Ther.

[7] Syed YY, McCormack PL, Plosker GL (2014). Bosutinib: a review of its use in patients with Philadelphia chromosome-positive chronic myelogenous leukemia. BioDrugs.

[8] Hantschel O, Grebien F, Superti-Furga G (2012). The growing arsenal of ATP-competitive and allosteric inhibitors of BCR-ABL. Cancer Res.

[9] Steegmann JL, Cervantes F, le Coutre P, Porkka K, Saglio G (2012). Off-target effects of BCR-ABL1 inhibitors and their potential long-term implications in patients with chronic myeloid leukemia. Leuk Lymphoma.

[10] Jabbour E, Cortes JE, Kantarjian HM (2009). Suboptimal response to or failure of imatinib treatment for chronic myeloid leukemia: what is the optimal strategy?. Mayo Clin Proc.

[11] Efficace F, Baccarani M, Breccia M (2011). Health-related quality of life in chronic myeloid leukemia patients receiving long-term therapy with imatinib compared with the general population. Blood.

[12] Laganà A, Scalzulli E, Carmosino I, Martelli M, Breccia M (2023). Asciminib as a third line option in chronic myeloid leukemia. Int J Hematol.

[13] Soverini S, Colarossi S, Gnani A (2006). Contribution of ABL kinase domain mutations to imatinib resistance in different subsets of Philadelphia-positive patients: by the GIMEMA Working Party on Chronic Myeloid Leukemia. Clin Cancer Res.

[14] O'Hare T, Eide CA, Deininger MW (2007). Bcr-Abl kinase domain mutations, drug resistance, and the road to a cure for chronic myeloid leukemia. Blood.

[15] Branford S, Melo JV, Hughes TP (2009). Selecting optimal second-line tyrosine kinase inhibitor therapy for chronic myeloid leukemia patients after imatinib failure: does the BCR-ABL mutation status really matter?. Blood.

[16] Ma W, Kantarjian H, Yeh CH, Zhang ZJ, Cortes J, Albitar M (2009). BCR-ABL truncation due to premature translation termination as a mechanism of resistance to kinase inhibitors. Acta Haematol.

[17] Soverini S, Branford S, Nicolini FE (2014). Implications of BCR-ABL1 kinase domain-mediated resistance in chronic myeloid leukemia. Leuk Res.

[18] Nath A, Wang J, Stephanie Huang R (2017). Pharmacogenetics and pharmacogenomics of targeted therapeutics in chronic myeloid leukemia. Mol Diagn Ther.

[19] Cargnin S, Ravegnini G, Soverini S, Angelini S, Terrazzino S (2018). Impact of SLC22A1 and CYP3A5 genotypes on imatinib response in chronic myeloid leukemia: a systematic review and meta-analysis. Pharmacol Res.

[20] Kaehler M, Cascorbi I (2023). Molecular mechanisms of tyrosine kinase inhibitor resistance in chronic myeloid leukemia. Handb Exp Pharmacol.

[21] Deininger MW, Druker BJ (2003). Specific targeted therapy of chronic myelogenous leukemia with imatinib. Pharmacol Rev.

[22] Vener C, Banzi R, Ambrogi F, Ferrero A, Saglio G, Pravettoni G, Sant M (2020). First-line imatinib vs second- and third-generation TKIs for chronic-phase CML: a systematic review and meta-analysis. Blood Adv.

[23] Barratt D, Somogyi A (2017). Role of pharmacogenetics in personalized imatinib dosing. Transl Cancer Res.

[24] Barratt DT, Cox HK, Menelaou A, Yeung DT, White DL, Hughes TP, Somogyi AA (2017). CYP2C8 genotype significantly alters imatinib metabolism in chronic myeloid leukaemia patients. Clin Pharmacokinet.

[25] Zhang N, Liu Y, Jeong H (2015). Drug-drug interaction potentials of tyrosine kinase inhibitors via inhibition of UDP-glucuronosyltransferases. Sci Rep.

[26] Filppula AM, Neuvonen M, Laitila J, Neuvonen PJ, Backman JT (2013). Autoinhibition of CYP3A4 leads to important role of CYP2C8 in imatinib metabolism: variability in CYP2C8 activity may alter plasma concentrations and response. Drug Metab Dispos.

[27] Quintás-Cardama A, Kantarjian HM, Cortes JE (2009). Mechanisms of primary and secondary resistance to imatinib in chronic myeloid leukemia. Cancer Control.

[28] Nies AT, Schaeffeler E, van der Kuip H (2014). Cellular uptake of imatinib into leukemic cells is independent of human organic cation transporter 1 (OCT1). Clin Cancer Res.

[29] Dessilly G, Panin N, Elens L, Haufroid V, Demoulin JB (2016). Impact of ABCB1 1236C > T-2677G > T-3435C > T polymorphisms on the anti-proliferative activity of imatinib, nilotinib, dasatinib and ponatinib. Sci Rep.

[30] Au A, Aziz Baba A, Goh AS, Wahid Fadilah SA, Teh A, Rosline H, Ankathil R (2014). Association of genotypes and haplotypes of multi-drug transporter genes ABCB1 and ABCG2 with clinical response to imatinib mesylate in chronic myeloid leukemia patients. Biomed Pharmacother.

[31] Bruckmueller H, Cascorbi I (2021). ABCB1, ABCG2, ABCC1, ABCC2, and ABCC3 drug transporter polymorphisms and their impact on drug bioavailability: what is our current understanding?. Expert Opin Drug Metab Toxicol.

[32] Kim DH, Sriharsha L, Xu W (2009). Clinical relevance of a pharmacogenetic approach using multiple candidate genes to predict response and resistance to imatinib therapy in chronic myeloid leukemia. Clin Cancer Res.

[33] Gandia P, Arellano C, Lafont T, Huguet F, Malard L, Chatelut E (2013). Should therapeutic drug monitoring of the unbound fraction of imatinib and its main active metabolite N-desmethyl-imatinib be developed?. Cancer Chemother Pharmacol.

[34] Petain A, Kattygnarath D, Azard J (2008). Population pharmacokinetics and pharmacogenetics of imatinib in children and adults. Clin Cancer Res.

[35] Taguchi K, Nishi K., Giam Chuang VT, Maruyam T, Otagiri M (2013). Molecular aspects of human alpha-1 acid glycoprotein — structure and function. Acute Phase Proteins.

[36] Cereja Pantoja KB, Azevedo TC, Carvalho DC (2022). Impact of variants in the ATIC and ARID5B genes on therapeutic failure with imatinib in patients with chronic myeloid leukemia. Genes (Basel).

[37] Zhao X, Qian M, Goodings C (2022). Molecular mechanisms of ARID5B-mediated genetic susceptibility to acute lymphoblastic leukemia. J Natl Cancer Inst.

[38] Prenggono MD, Yasmina A, Ariyah M, Wanahari TA, Hasrianti N (2021). The effect of imatinib and nilotinib on blood calcium and blood potassium levels in chronic myeloid leukemia patients: a literature review. Oncol Rev.

[39] Wenger TL, Bly RA, Wu N (2020). Activating variants in PDGFRB result in a spectrum of disorders responsive to imatinib monotherapy. Am J Med Genet A.

[40] Nakagawa H, Takiguchi T, Nakamura M (2006). Basis for dosing time-dependent change in the anti-tumor effect of imatinib in mice. Biochem Pharmacol.

[41] Bhatia R (2017). Novel approaches to therapy in CML. Hematology Am Soc Hematol Educ Program.

[42] Talati C, Pinilla-Ibarz J (2018). Resistance in chronic myeloid leukemia: definitions and novel therapeutic agents. Curr Opin Hematol.

[43] Arrigoni E, Del Re M, Galimberti S (2018). Concise Review: Chronic Myeloid Leukemia: Stem Cell Niche and Response to Pharmacologic Treatment. Stem Cells Transl Med.

[44] Braun TP, Eide CA, Druker BJ (2020). Response and Resistance to BCR-ABL1-Targeted Therapies. Cancer Cell.

[45] Blay JY, von Mehren M (2011). Nilotinib: a novel, selective tyrosine kinase inhibitor. Semin Oncol.

[46] Cui X, Sun J, Li C, Qiu S, Shi C, Ma J, Xu Y (2022). Downregulation of hERG channel expression by tyrosine kinase inhibitors nilotinib and vandetanib predominantly contributes to arrhythmogenesis. Toxicol Lett.

[47] Carracedo M, Pawelzik SC, Artiach G (2022). The tyrosine kinase inhibitor nilotinib targets the discoidin domain receptor DDR2 in calcific aortic valve stenosis. Br J Pharmacol.

[48] Li S, He J, Zhang X, Cai Y, Liu J, Nie X, Shi L (2022). Cardiovascular adverse events in chronic myeloid leukemia patients treated with nilotinib or imatinib: A systematic review, meta-analysis and integrative bioinformatics analysis. Front Cardiovasc Med.

[49] Abumiya M, Takahashi N, Niioka T (2014). Influence of UGT1A1 6, 27, and 28 polymorphisms on nilotinib-induced hyperbilirubinemia in Japanese patients with chronic myeloid leukemia. Drug Metab Pharmacokinet.

[50] Shibata T, Minami Y, Mitsuma A (2014). Association between severe toxicity of nilotinib and UGT1A1 polymorphisms in Japanese patients with chronic myelogenous leukemia. Int J Clin Oncol.

[51] Bykova A, Abdullayev AO, Gusarova G, Chelysheva E, Treglazova S, Smirnova S, Turkina A (2014). Polymorphism of UGT1A1 and frequency of hyperbilirubinemia in patients with chronic myeloid leukemia treated by nilotinib. Blood.

[52] Eadie LN, Hughes TP, White DL (2014). Interaction of the efflux transporters ABCB1 and ABCG2 with imatinib, nilotinib, and dasatinib. Clin Pharmacol Ther.

[53] Galimberti S, Bucelli C, Arrigoni E (2017). The hOCT1 and ABCB1 polymorphisms do not influence the pharmacodynamics of nilotinib in chronic myeloid leukemia. Oncotarget.

[54] Dessilly G, Elens L, Panin N, Karmani L, Demoulin JB, Haufroid V (2016). ABCB1 1199G>A polymorphism (rs2229109) affects the transport of imatinib, nilotinib and dasatinib. Pharmacogenomics.

[55] Hebron M, Peyton M, Liu X, Gao X, Wang R, Lonskaya I, Moussa CE (2017). Discoidin domain receptor inhibition reduces neuropathology and attenuates inflammation in neurodegeneration models. J Neuroimmunol.

[56] Pagan FL, Hebron ML, Wilmarth B (2019). Pharmacokinetics and pharmacodynamics of a single dose nilotinib in individuals with Parkinson's disease. Pharmacol Res Perspect.

[57] Nekoukar Z, Moghimi M, Salehifar E (2021). A narrative review on adverse effects of dasatinib with a focus on pharmacotherapy of dasatinib-induced pulmonary toxicities. Blood Res.

[58] Chen R, Chen B (2015). The role of dasatinib in the management of chronic myeloid leukemia. Drug Des Devel Ther.

[59] El-Dabh A, Acharya D (2019). Pulmonary hypertension with dasatinib and other tyrosine kinase inhibitors. Pulm Circ.

[60] Jabbour E, Fullmer A, Cortés JE, Kantarjian H (2010). Clinical algorithms for the treatment of patients with chronic myeloid leukemia: the 2010 perspective. Clin Lymphoma Myeloma Leuk.

[61] Christopher LJ, Cui D, Wu C (2008). Metabolism and disposition of dasatinib after oral administration to humans. Drug Metab Dispos.

[62] He S, Bian J, Shao Q (2021). Therapeutic drug monitoring and individualized medicine of dasatinib: focus on clinical pharmacokinetics and pharmacodynamics. Front Pharmacol.

[63] Xu Z, Cang S, Yang T, Liu D (2009). Cardiotoxicity of tyrosine kinase inhibitors in chronic myelogenous leukemia therapy. Hematol Rev.

[64] Guignabert C, Phan C, Seferian A (2016). Dasatinib induces lung vascular toxicity and predisposes to pulmonary hypertension. J Clin Invest.

[65] Gurion R, Gafter-Gvili A, Vidal L (2013). Has the time for first-line treatment with second generation tyrosine kinase inhibitors in patients with chronic myelogenous leukemia already come? Systematic review and meta-analysis. Haematologica.

[66] Steinbach A, Clark SM, Clemmons AB (2013). Bosutinib: a novel src/abl kinase inhibitor for chronic myelogenous leukemia. J Adv Pract Oncol.

[67] Cortes JE, Kantarjian HM, Brümmendorf TH (2011). Safety and efficacy of bosutinib (SKI-606) in chronic phase Philadelphia chromosome-positive chronic myeloid leukemia patients with resistance or intolerance to imatinib. Blood.

[68] Gambacorti-Passerini C, Cortes JE, Lipton JH (2014). Safety of bosutinib versus imatinib in the phase 3 BELA trial in newly diagnosed chronic phase chronic myeloid leukemia. Am J Hematol.

[69] Amsberg GK, Schafhausen P (2013). Bosutinib in the management of chronic myelogenous leukemia. Biologics.

[70] Mita A, Abumiya M, Takahashi S, Kameoka Y, Miura M, Takahashi N (2018). Pharmacokinetic and pharmacogenetic analysis of bosutinib in japanese patients with chronic-phase chronic myeloid leukemia. Blood.

[71] Gambacorti-Passerini C, le Coutre P, Piazza R (2020). The role of bosutinib in the treatment of chronic myeloid leukemia. Future Oncol.

[72] Cui Z, Li B, Zhang Y (2021). Inhibition of soluble epoxide hydrolase attenuates bosutinib-induced blood pressure elevation. Hypertension.

[73] Hicks JK, El Rouby N, Ong HH (2021). Opportunity for genotype-guided prescribing among adult patients in 11 US health systems. Clin Pharmacol Ther.

[74] Ogu CC, Maxa JL (2000). Drug interactions due to cytochrome P450. Proc (Bayl Univ Med Cent).

